# Supplementary material for: Assessing the Presence of Recent Adaptation in the Human Genome With Mixture Density Regression
Source: Genome Biol Evol. 2023 Sep 15;15(10):evad170. doi: 10.1093/gbe/evad170 (PMC10563788; doi:10.1093/gbe/evad170)
Supplement: evad170_Supplementary_Data [file evad170_supplementary_data.pdf]

Supplemental Results S1: Results obtained using classic linear models and partial correlations

Classical partial correlations

We have performed classic partial correlations (Spearman's rank correlations) between the whole iHS distribution and individual genomic factors while controlling for the rest of factors. See results for some functional factors expected to be associated with positive selection.

Covariate	Yoruba		Toscani		Utah residents		Han Chinese		Peruvians	
	ρ	p-value	ρ	p-value	ρ	p-value	ρ	p-value	ρ	p-value
Density of conserved elements	-0.033	3.88E-05	0.053	5.16E-11	0.046	8.93E-09	0.039	1.48E-06	0.056	2.63E-12
Distance to VIPs	-0.012	1.46E-01	-0.007	4.07E-01	-0.002	7.69E-01	0.005	4.98E-01	-0.027	8.47E-04
Gene expression in immune cells	0.026	9.57E-04	0.042	1.79E-07	0.040	5.58E-07	0.032	5.44E-05	0.022	5.28E-03
Regulatory density (ChIP-seq)	-0.029	2.77E-04	0.036	7.21E-06	0.007	3.60E-01	-0.005	5.70E-01	0.002	8.50E-01
Coding density	0.036	6.67E-06	0.045	2.13E-08	0.050	5.57E-10	0.052	5.49E-11	0.041	2.55E-07

Table S1: Correlation coefficients (Spearman's ρ) and p-values of the association between iHS and genomic factors for the five studied populations in 1,000 kb windows: Yoruba, Toscani, Utah residents, Han Chinese and Peruvians.

Classical linear models

We have run classic linear models considering the whole iHS distribution as response and genomic factors as predictors. See results for some functional factors expected to be associated with recent positive selection.

Covariate	Yoruba		Toscani		Utah residents		Han Chinese		Peruvians	
	slope	p-value	slope	p-value	slope	p-value	slope	p-value	slope	p-value
Density of conserved elements	0.005	6.25E-01	0.085	2.28E-16	0.086	5.89E-17	0.085	6.61E-16	0.062	4.61E-09
Distance to VIPs	-0.024	2.87E-03	-0.042	3.12E-07	-0.042	2.53E-07	-0.028	9.80E-04	-0.034	5.82E-05
Gene expression in immune cells	0.062	1.13E-05	0.090	1.22E-10	0.097	3.86E-12	0.073	3.51E-07	0.060	3.19E-05
Regulatory density (ChIP-seq)	-0.036	1.10E-01	0.136	9.71E-10	0.088	7.88E-05	0.036	1.13E-01	-0.043	5.98E-02
Coding density	0.037	6.68E-02	0.092	4.89E-06	0.089	8.49E-06	0.104	4.16E-07	0.068	1.02E-03

Table S2: Slopes and p-values of the association between iHS and genomic factors for the five studied populations in 1,000 kb windows: Yoruba, Toscani, Utah residents, Han Chinese and Peruvians.

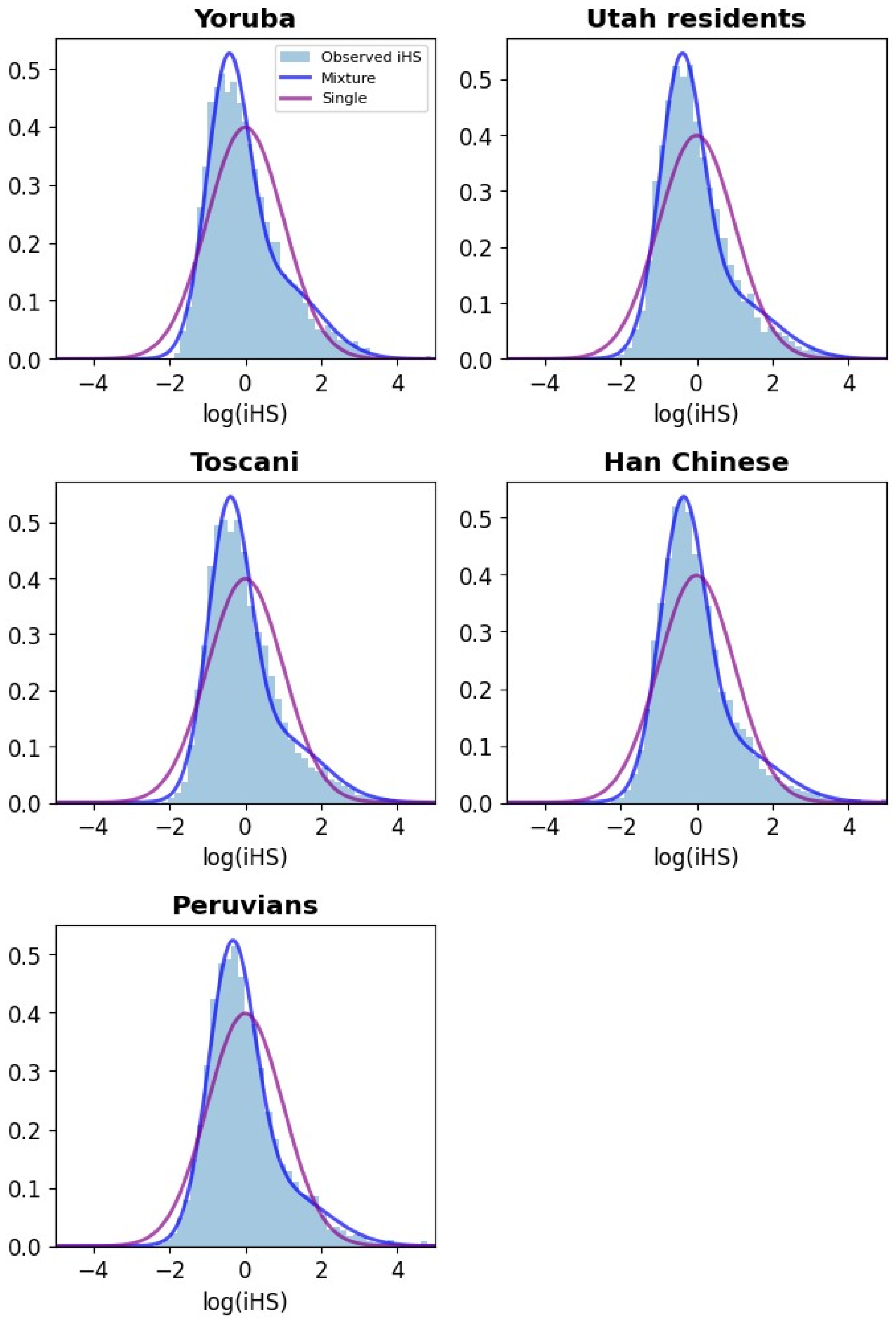

Figure S1: Mixture of two Gaussian distributions and a single Gaussian distribution fitting observed iHS (1,000 kb windows) for the five studied populations: A) Africa – Yoruba; B) Europe – Utah residents with Northern and Western European ancestry; C) Europe – Toscani; D) East Asia – Han Chinese; E) America – Peruvians. For each population, the figure shows an histogram of the observed iHS (1,000 kb). It also shows a single Gaussian distribution (magenta solid line) with the mean

and standard deviation of the observed iHS, along with a mixture of two Gaussian distributions (blue solid line) based on the MDR approach (see Methods for details).

Supplemental Results S2: Associations between genomic factors and iHS across populations and window sizes

Yoruba

Yoruba 50kb

Figure S1: Mixture of Gaussian distributions fitting observed iHS (50kb windows) for Yoruba. The figure shows the two Gaussian distributions, component 1 and 2 of iHS, being the latter enriched in positive selection. In that component, iHS linearly depends on the genomic factors considered. The figure shows iHS after log transformation and scaling (see Methods). Legend: Light blue = Observed iHS; Dark blue = Mixture model; Full red curve = Component 1 of the mixture model; Dashed red curve = Component 2 of the mixture model enriched in positive selection.

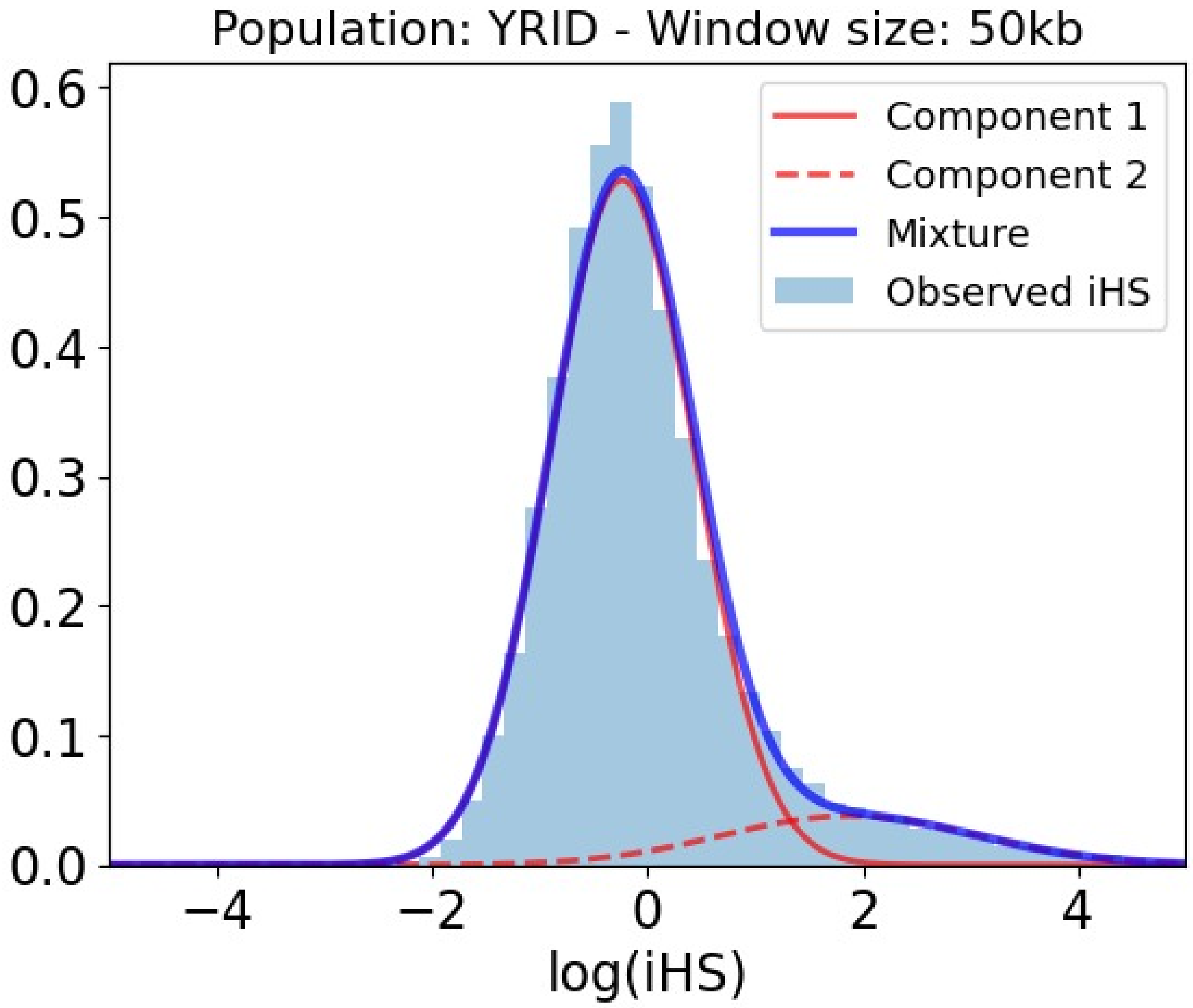

Table S1: Slopes and p-values of the association between iHS and genomic factors for the Yoruba population in 50kb within the selection-enriched component.

**Covariate**	**Slope**	**P-value**
Intercept	-115.603	0.000E+00
Number iHS data points	0.804	0.000E+00
Density of conserved elements	-0.069	5.897E-01
Recombination rate	-180.136	0.000E+00
Number PPIs	0.116	9.713E-02
Regulatory density (ChIP-seq)	0.211	3.791E-01
Distance to VIPs	-0.025	7.000E-01
Gene number	-0.012	9.123E-01
Coding density	0.054	6.526E-01
Gene length	-0.077	4.490E-01
Regulatory density in immune cells (ChIP-seq)	-0.447	5.618E-02

Covariate	Slope	P-value
Gene expression	0.220	2.447E-01
Gene expression in testis	0.223	4.685E-02
Gene expression in immune cells	0.007	9.561E-01
Regulatory density in testis (ChIP-seq)	-0.130	2.602E-01
Regulatory density (DNaseI)	0.188	4.407E-01
GC-content	-0.323	6.891E-02

Yoruba 100kb

Figure S2: Mixture of Gaussian distributions fitting observed iHS (100kb windows) for Yoruba. The figure shows the two Gaussian distributions, component 1 and 2 of iHS, being the latter enriched in positive selection. In that component, iHS linearly depends on the genomic factors considered. The figure shows iHS after log transformation and scaling (see Methods). Legend: Light blue = Observed iHS; Dark blue = Mixture model; Full red curve = Component 1 of the mixture model; Dashed red curve = Component 2 of the mixture model enriched in positive selection.

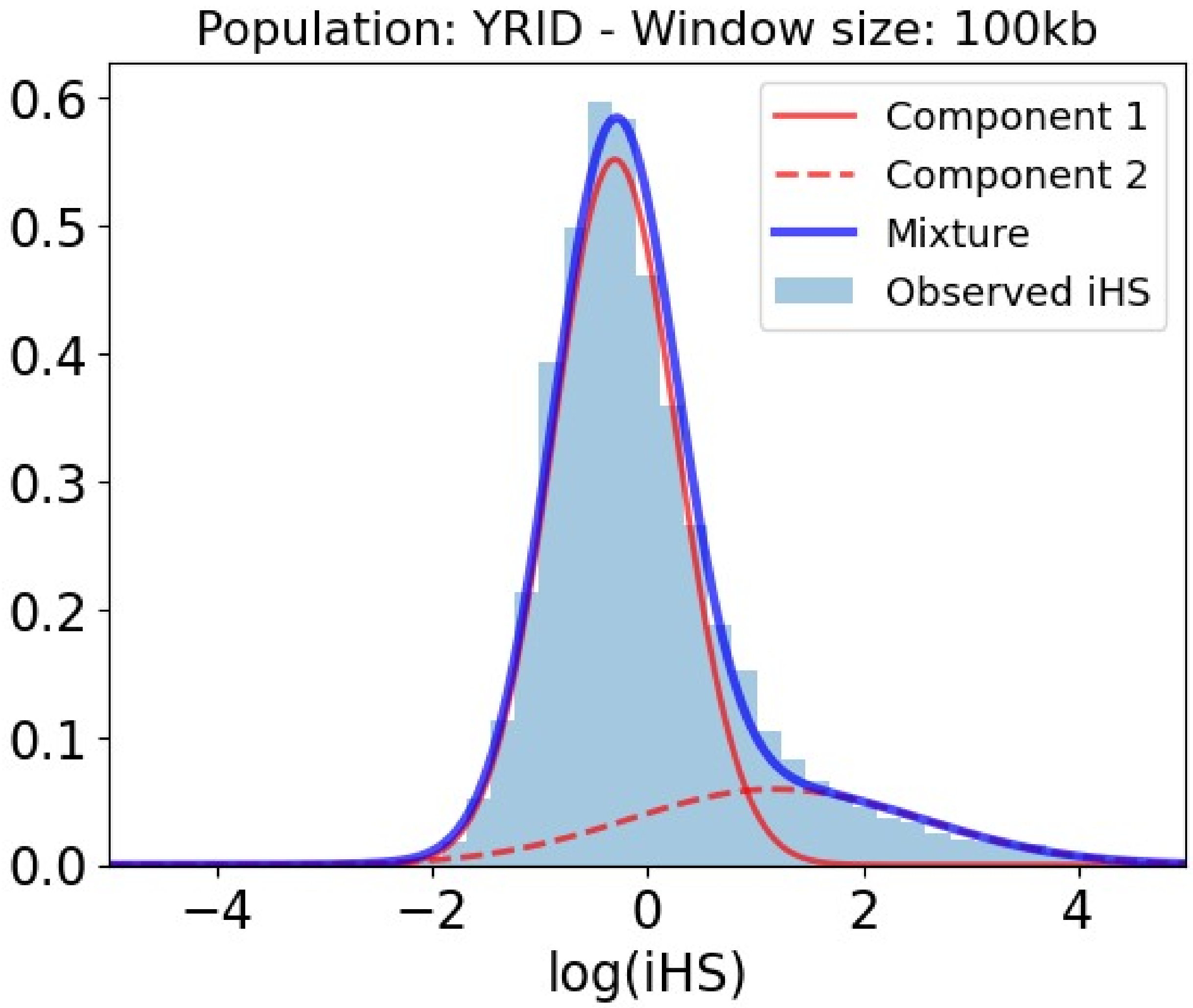

Table S2: Slopes and p-values of the association between iHS and genomic factors for the Yoruba population in 100kb within the selection-enriched component.

**Covariate**	**Slope**	**P-value**
Intercept	-2.740	0.000E+00
Number iHS data points	-0.143	1.919E-04
Density of conserved elements	0.097	9.301E-02
Recombination rate	-2.680	0.000E+00
Number PPIs	0.040	2.492E-01
Regulatory density (ChIP-seq)	-0.186	4.779E-02
Distance to VIPs	-0.024	5.115E-01
Gene number	-0.095	1.096E-01
Coding density	0.140	2.953E-02
Gene length	-0.040	3.348E-01
Regulatory density in immune cells (ChIP-seq)	-0.326	1.181E-03

Covariate	Slope	P-value
Gene expression	-0.082	3.189E-01
Gene expression in testis	0.200	3.742E-04
Gene expression in immune cells	0.434	2.118E-08
Regulatory density in testis (ChIP-seq)	-0.038	4.838E-01
Regulatory density (DNaseI)	-0.102	3.672E-01
GC-content	-0.253	5.460E-03

Yoruba 200kb

Figure S3: Mixture of Gaussian distributions fitting observed iHS (200kb windows) for Yoruba. The figure shows the two Gaussian distributions, component 1 and 2 of iHS, being the latter enriched in positive selection. In that component, iHS linearly depends on the genomic factors considered. The figure shows iHS after log transformation and scaling (see Methods). Legend: Light blue = Observed iHS; Dark blue = Mixture model; Full red curve = Component 1 of the mixture model; Dashed red curve = Component 2 of the mixture model enriched in positive selection.

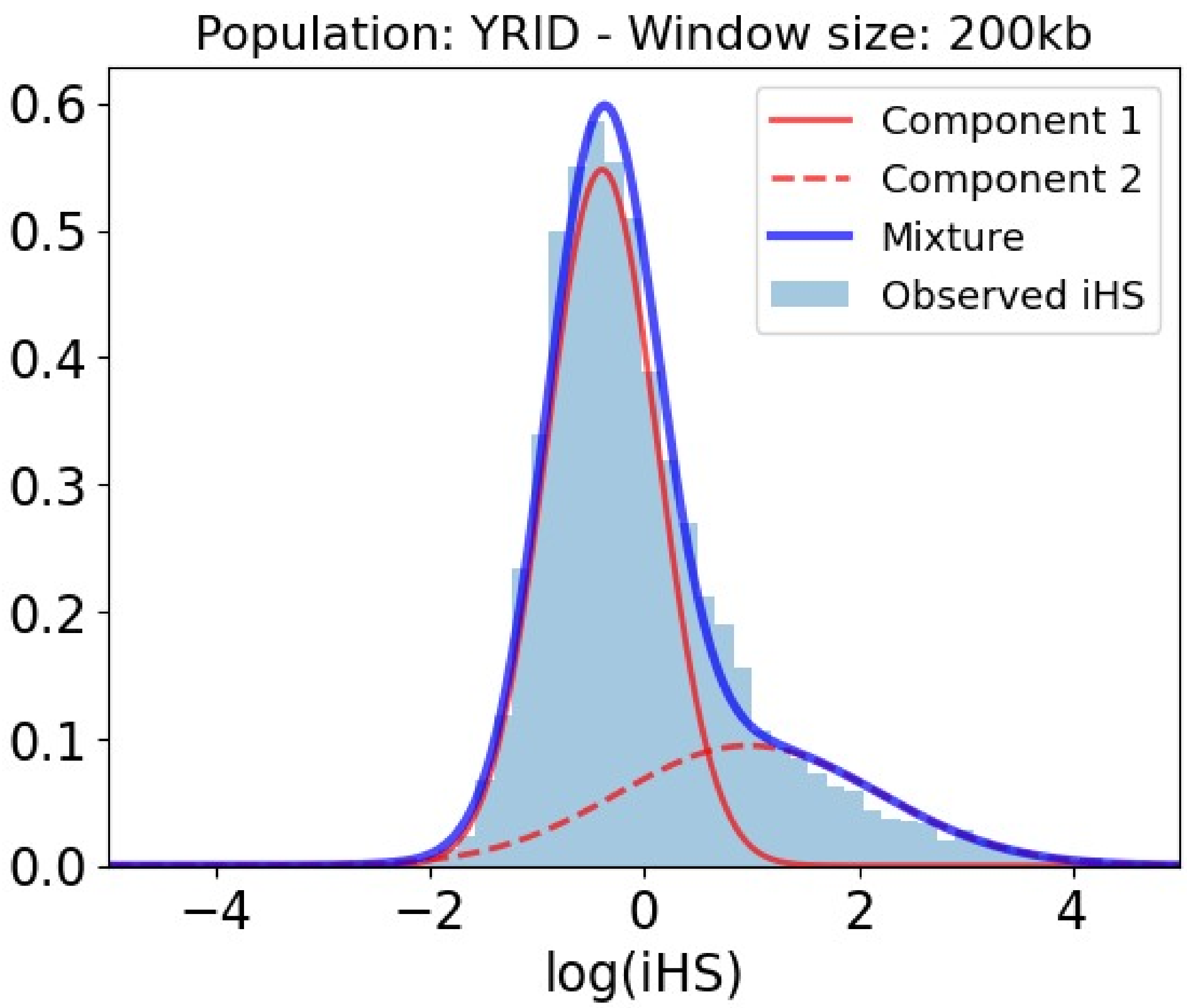

Table S3: Slopes and p-values of the association between iHS and genomic factors for the Yoruba population in 200kb within the selection-enriched component.

Covariate	Slope	P-value
Intercept	-1.377	0.000E+00
Number iHS data points	-0.336	0.000E+00
Density of conserved elements	-0.058	2.247E-01
Recombination rate	-1.290	0.000E+00
Number PPIs	0.022	4.770E-01
Regulatory density (ChIP-seq)	-0.041	6.043E-01
Distance to VIPs	-0.046	1.347E-01
Gene number	0.032	5.768E-01
Coding density	0.083	1.708E-01
Gene length	-0.010	7.725E-01
Regulatory density in immune cells (ChIP-seq)	-0.197	7.978E-03

Covariate	Slope	P-value
Gene expression	-0.127	5.274E-02
Gene expression in testis	0.167	1.668E-04
Gene expression in immune cells	0.362	2.182E-09
Regulatory density in testis (ChIP-seq)	-0.047	3.231E-01
Regulatory density (DNaseI)	-0.088	3.746E-01
GC-content	-0.181	2.904E-02

Yoruba 500kb

Figure S4: Mixture of Gaussian distributions fitting observed iHS (500kb windows) for Yoruba. The figure shows the two Gaussian distributions, component 1 and 2 of iHS, being the latter enriched in positive selection. In that component, iHS linearly depends on the genomic factors considered. The figure shows iHS after log transformation and scaling (see Methods). Legend: Light blue = Observed iHS; Dark blue = Mixture model; Full red curve = Component 1 of the mixture model; Dashed red curve = Component 2 of the mixture model enriched in positive selection.

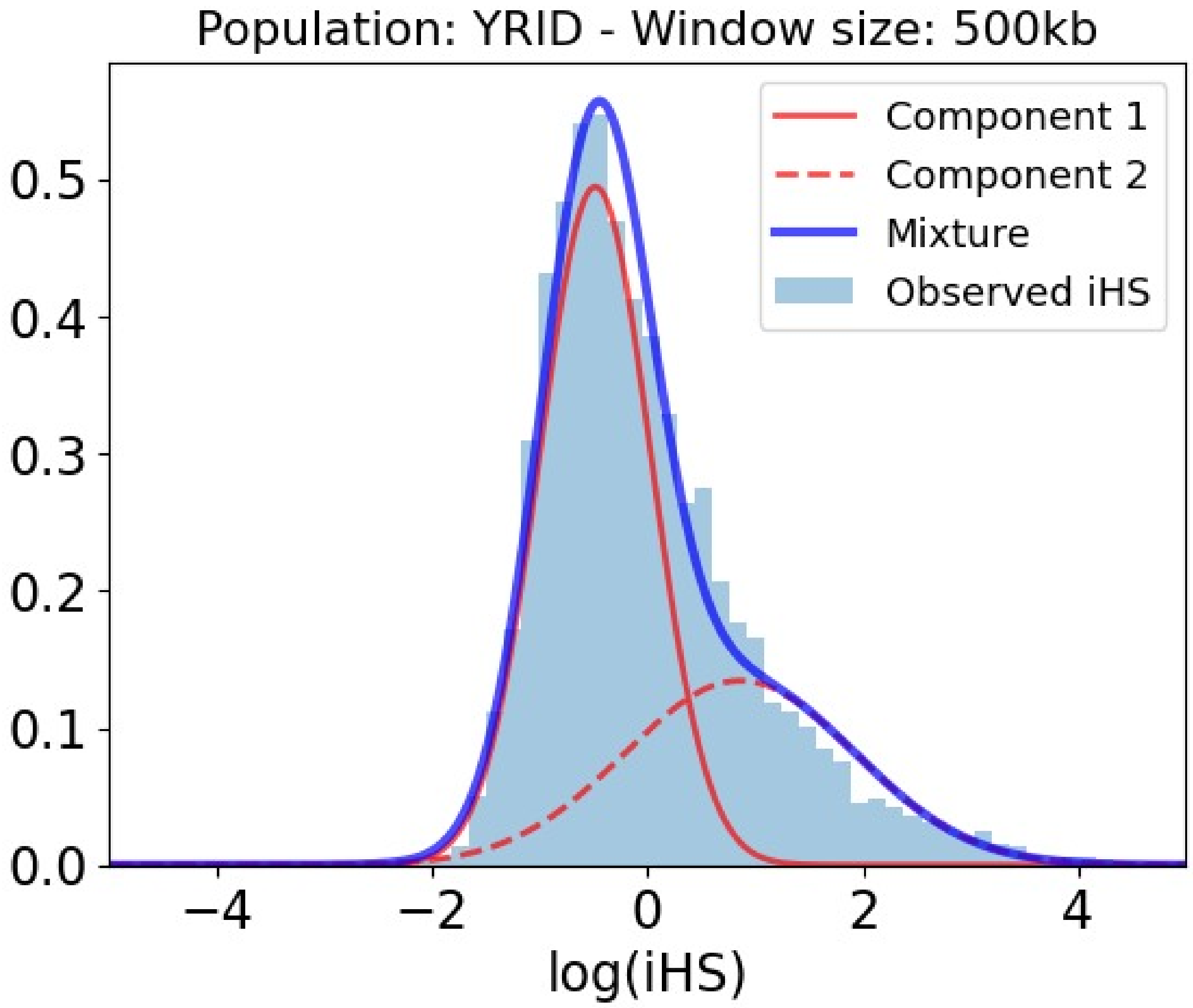

Table S4: Slopes and p-values of the association between iHS and genomic factors for the Yoruba population in 500kb within the selection-enriched component.

**Covariate**	**Slope**	**P-value**
Intercept	-0.994	0.000E+00
Number iHS data points	-0.305	3.331E-16
Density of conserved elements	-0.030	4.976E-01
Recombination rate	-1.560	0.000E+00
Number PPIs	-0.044	1.559E-01
Regulatory density (ChIP-seq)	-0.002	9.915E-01
Distance to VIPs	-0.090	4.146E-03
Gene number	0.009	9.019E-01
Coding density	0.075	2.787E-01
Gene length	-0.048	1.707E-01
Regulatory density in immune cells (ChIP-seq)	0.092	1.891E-01

Covariate	Slope	P-value
Gene expression	-0.123	4.693E-02
Gene expression in testis	0.137	1.248E-03
Gene expression in immune cells	0.200	3.962E-04
Regulatory density in testis (ChIP-seq)	-0.510	0.000E+00
Regulatory density (DNaseI)	0.062	5.669E-01
GC-content	-0.035	6.941E-01

Yoruba 1000kb

Figure S5: Mixture of Gaussian distributions fitting observed iHS (1000kb windows) for Yoruba. The figure shows the two Gaussian distributions, component 1 and 2 of iHS, being the latter enriched in positive selection. In that component, iHS linearly depends on the genomic factors considered. The figure shows iHS after log transformation and scaling (see Methods). Legend: Light blue = Observed iHS; Dark blue = Mixture model; Full red curve = Component 1 of the mixture model; Dashed red curve = Component 2 of the mixture model enriched in positive selection.

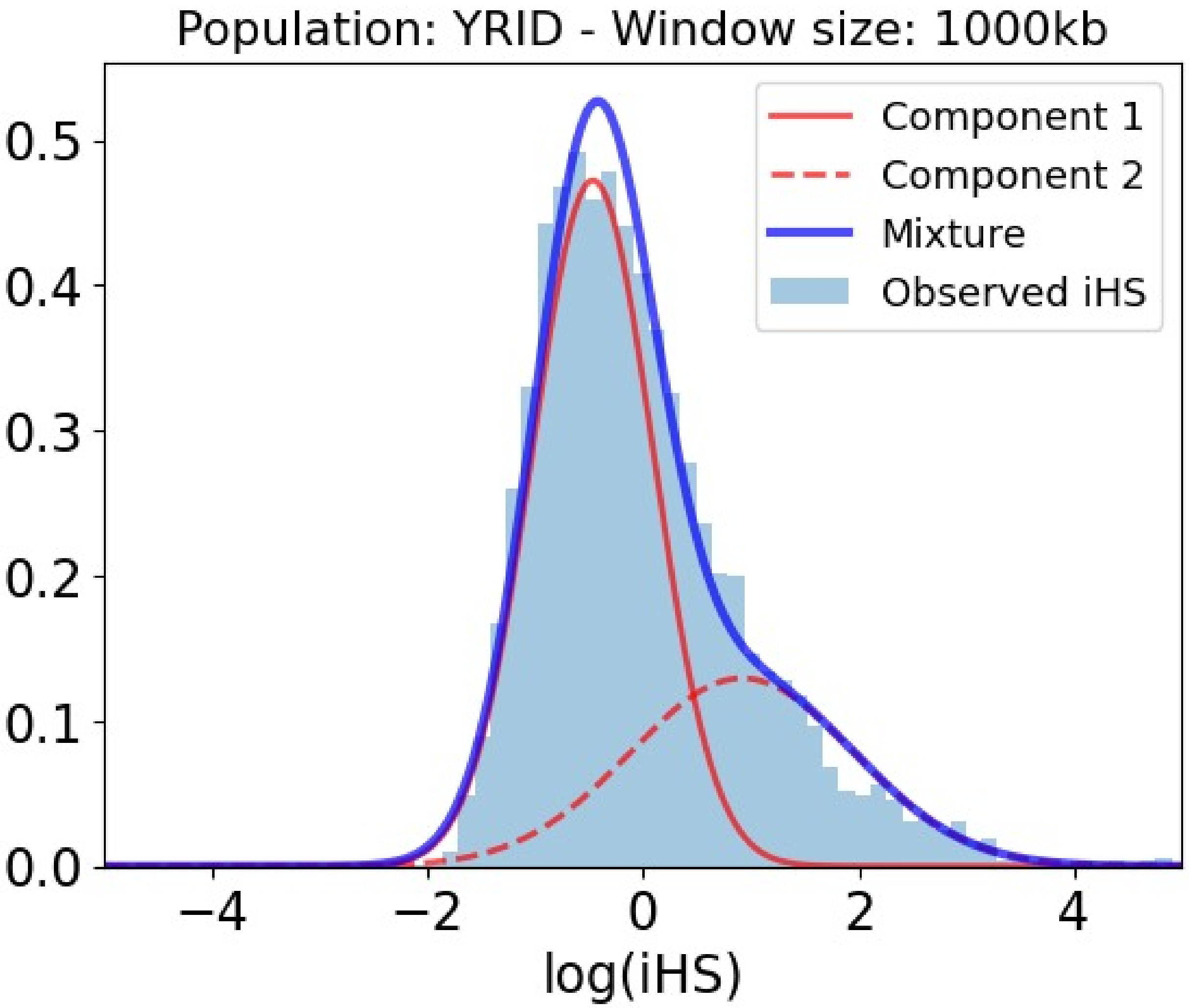

Table S5: Slopes and p-values of the association between iHS and genomic factors for the Yoruba population in 1000kb within the selection-enriched component.

**Covariate**	**Slope**	**P-value**
Intercept	-1.426	0.000E+00
Number iHS data points	-0.007	8.193E-01
Density of conserved elements	0.159	6.401E-04
Recombination rate	-2.435	0.000E+00
Number PPIs	-0.070	4.252E-02
Regulatory density (ChIP-seq)	-0.147	2.372E-01
Distance to VIPs	-0.170	4.161E-06
Gene number	-0.086	3.458E-01
Coding density	0.159	8.412E-02
Gene length	-0.059	1.125E-01
Regulatory density in immune cells (ChIP-seq)	0.047	6.243E-01

Covariate	Slope	P-value
Gene expression	-0.139	3.768E-02
Gene expression in testis	0.001	9.900E-01
Gene expression in immune cells	0.265	1.512E-05
Regulatory density in testis (ChIP-seq)	-0.795	0.000E+00
Regulatory density (DNaseI)	-0.047	7.149E-01
GC-content	0.550	1.340E-06

Utah residents

Utah residents 50kb

Figure S6: Mixture of Gaussian distributions fitting observed iHS (50kb windows) for Utah residents. The figure shows the two Gaussian distributions, component 1 and 2 of iHS, being the latter enriched in positive selection. In that component, iHS linearly depends on the genomic factors considered. The figure shows iHS after log transformation and scaling (see Methods). Legend: Light blue = Observed iHS; Dark blue = Mixture model; Full red curve = Component 1 of the mixture model; Dashed red curve = Component 2 of the mixture model enriched in positive selection.

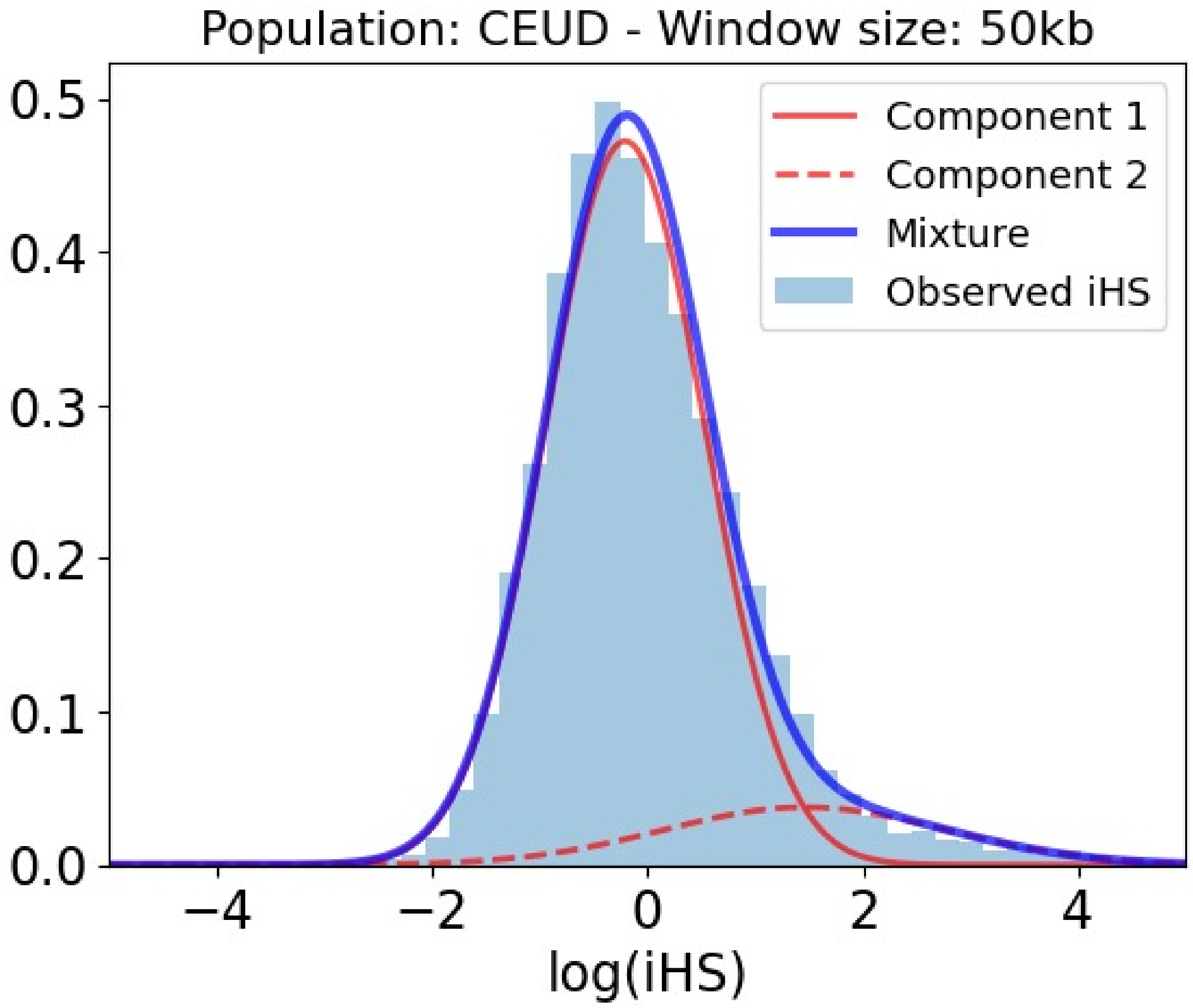

Table S6: Slopes and p-values of the association between iHS and genomic factors for the Utah residents population in 50kb within the selection-enriched component.

**Covariate**	**Slope**	**P-value**
Intercept	-99.047	0.000E+00
Number iHS data points	0.966	0.000E+00
Density of conserved elements	-0.108	5.375E-01
Recombination rate	-154.830	0.000E+00
Number PPIs	0.140	1.122E-01
Regulatory density (ChIP-seq)	0.628	1.056E-02
Distance to VIPs	-0.082	2.687E-01
Gene number	0.165	2.155E-01
Coding density	0.076	6.096E-01
Gene length	0.328	1.359E-02
Regulatory density in immune cells (ChIP-seq)	-0.984	1.394E-04

**Covariate**	**Slope**	**P-value**
Gene expression	0.010	9.667E-01
Gene expression in testis	0.458	2.294E-03
Gene expression in immune cells	0.250	3.006E-01
Regulatory density in testis (ChIP-seq)	0.144	3.101E-01
Regulatory density (DNaseI)	-0.338	2.616E-01
GC-content	-0.067	7.399E-01

Utah residents 100kb

Figure S7: Mixture of Gaussian distributions fitting observed iHS (100kb windows) for Utah residents. The figure shows the two Gaussian distributions, component 1 and 2 of iHS, being the latter enriched in positive selection. In that component, iHS linearly depends on the genomic factors considered. The figure shows iHS after log transformation and scaling (see Methods). Legend: Light blue = Observed iHS; Dark blue = Mixture model; Full red curve = Component 1 of the mixture model; Dashed red curve = Component 2 of the mixture model enriched in positive selection.

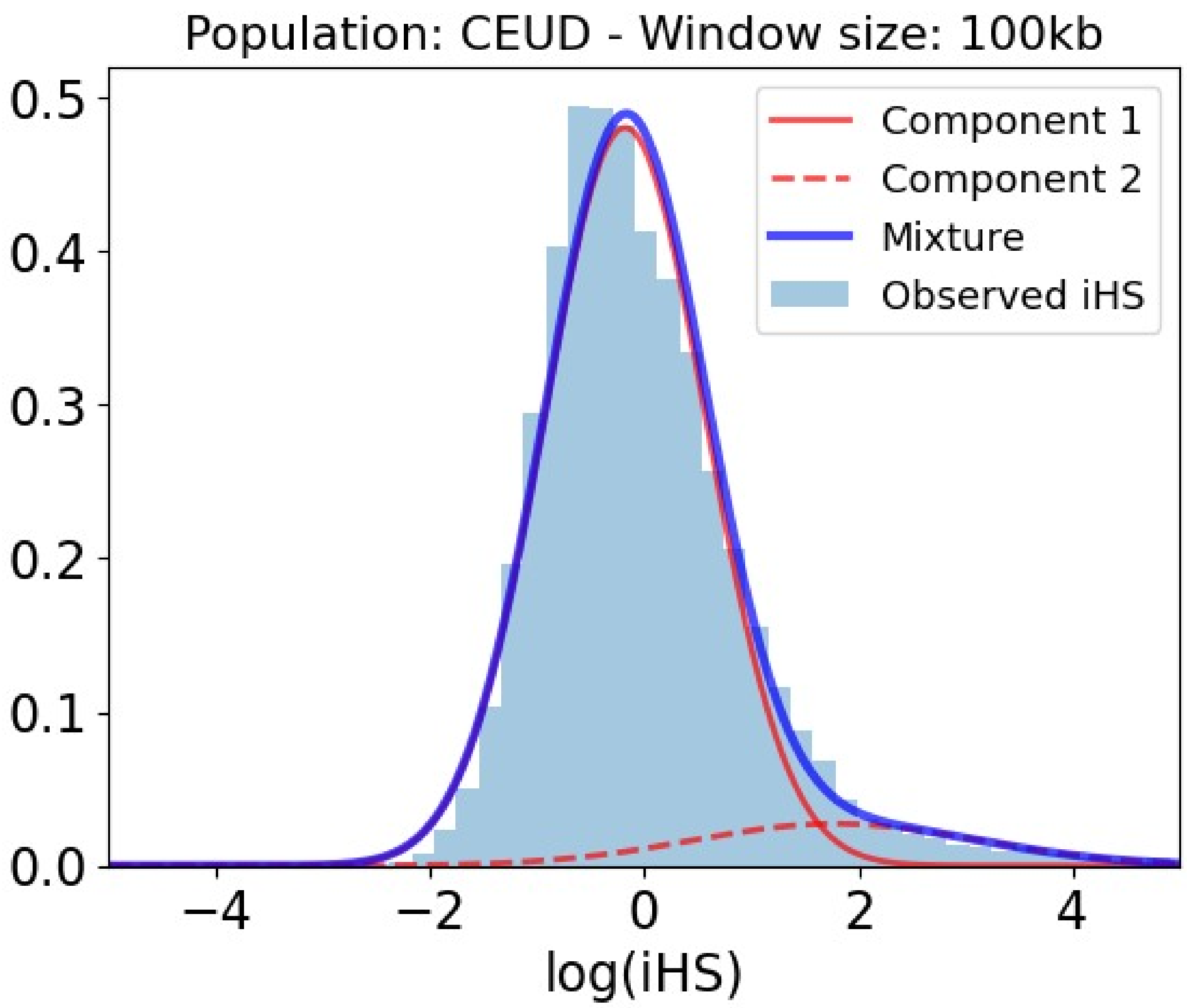

Table S7: Slopes and p-values of the association between iHS and genomic factors for the Utah residents population in 100kb within the selection-enriched component.

**Covariate**	**Slope**	**P-value**
Intercept	-41.468	0.000E+00
Number iHS data points	0.558	2.960E-13
Density of conserved elements	-0.028	8.499E-01
Recombination rate	-52.197	0.000E+00
Number PPIs	0.080	2.794E-01
Regulatory density (ChIP-seq)	-0.144	4.999E-01
Distance to VIPs	-0.087	2.095E-01
Gene number	0.048	7.240E-01
Coding density	-0.005	9.818E-01
Gene length	0.047	6.440E-01
Regulatory density in immune cells (ChIP-seq)	-0.013	9.515E-01

Covariate	Slope	P-value
Gene expression	0.112	5.865E-01
Gene expression in testis	0.341	4.043E-03
Gene expression in immune cells	0.304	8.982E-02
Regulatory density in testis (ChIP-seq)	-0.341	1.194E-02
Regulatory density (DNaseI)	-0.117	6.535E-01
GC-content	-0.034	8.598E-01

Utah residents 200kb

Figure S8: Mixture of Gaussian distributions fitting observed iHS (200kb windows) for Utah residents. The figure shows the two Gaussian distributions, component 1 and 2 of iHS, being the latter enriched in positive selection. In that component, iHS linearly depends on the genomic factors considered. The figure shows iHS after log transformation and scaling (see Methods). Legend: Light blue = Observed iHS; Dark blue = Mixture model; Full red curve = Component 1 of the mixture model; Dashed red curve = Component 2 of the mixture model enriched in positive selection.

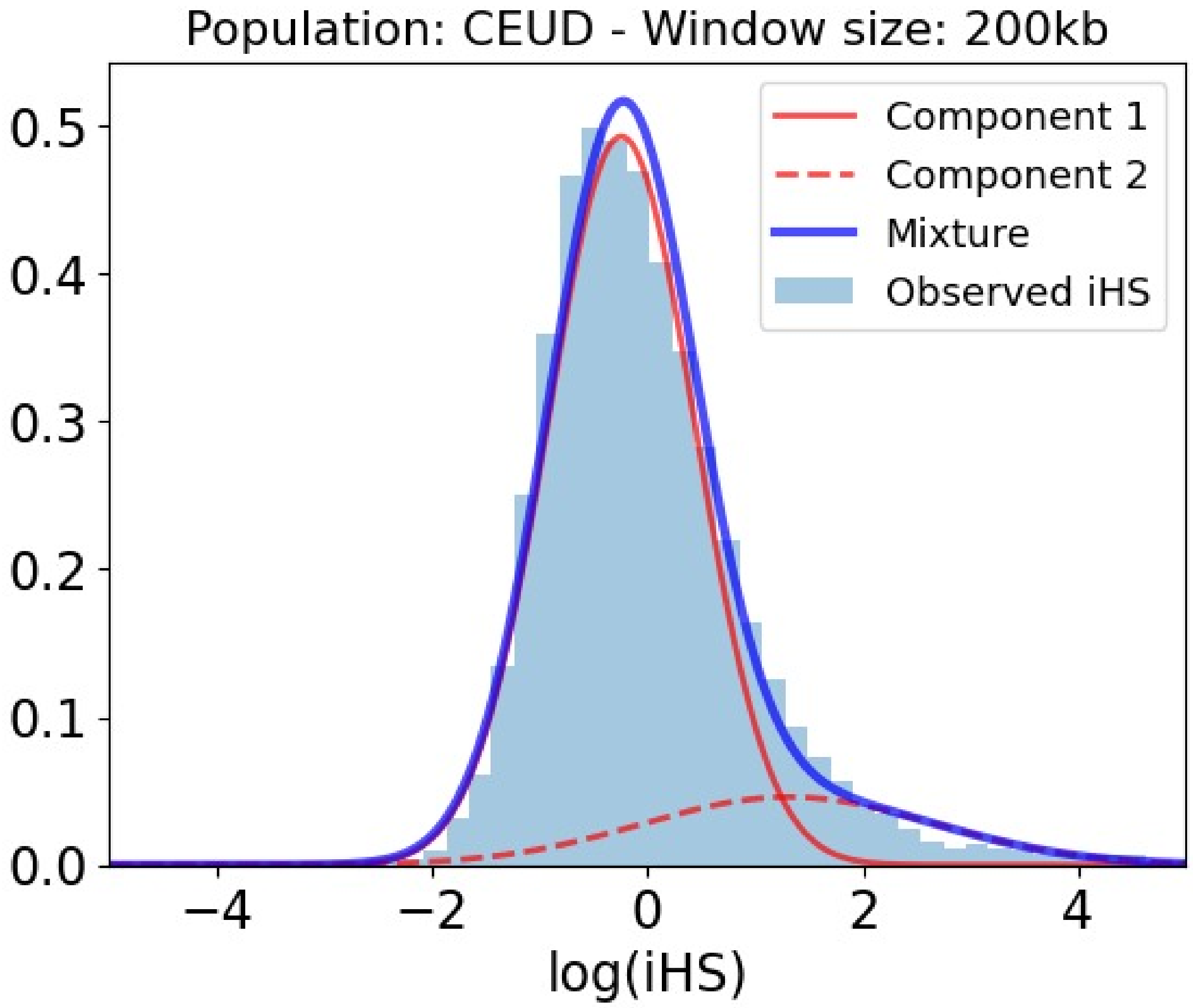

Table S8: Slopes and p-values of the association between iHS and genomic factors for the Utah residents population in 200kb within the selection-enriched component.

**Covariate**	**Slope**	**P-value**
Intercept	-3.400	0.000E+00
Number iHS data points	0.035	1.692E-01
Density of conserved elements	0.262	7.451E-05
Recombination rate	-3.015	0.000E+00
Number PPIs	0.047	2.519E-01
Regulatory density (ChIP-seq)	-0.200	9.420E-02
Distance to VIPs	-0.165	1.175E-04
Gene number	-0.312	1.112E-03
Coding density	0.208	1.838E-02
Gene length	0.044	3.334E-01
Regulatory density in immune cells (ChIP-seq)	-0.142	2.101E-01

Covariate	Slope	P-value
Gene expression	-0.265	7.272E-03
Gene expression in testis	0.192	3.291E-03
Gene expression in immune cells	0.576	1.895E-10
Regulatory density in testis (ChIP-seq)	-0.124	7.328E-02
Regulatory density (DNaseI)	-0.140	3.582E-01
GC-content	-0.123	2.845E-01

Utah residents 500kb

Figure S9: Mixture of Gaussian distributions fitting observed iHS (500kb windows) for Utah residents. The figure shows the two Gaussian distributions, component 1 and 2 of iHS, being the latter enriched in positive selection. In that component, iHS linearly depends on the genomic factors considered. The figure shows iHS after log transformation and scaling (see Methods). Legend: Light blue = Observed iHS; Dark blue = Mixture model; Full red curve = Component 1 of the mixture model; Dashed red curve = Component 2 of the mixture model enriched in positive selection.

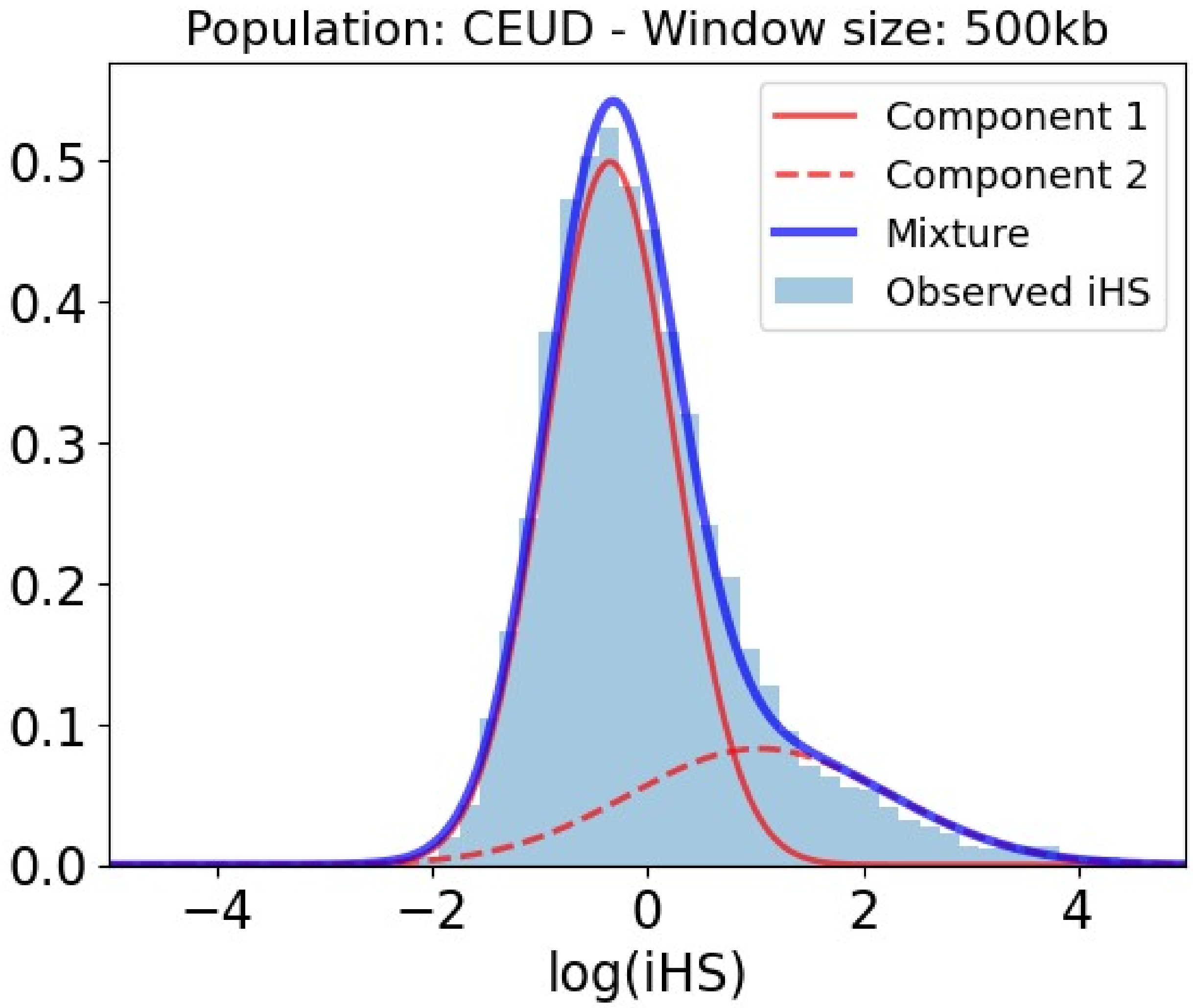

Table S9: Slopes and p-values of the association between iHS and genomic factors for the Utah residents population in 500kb within the selection-enriched component.

**Covariate**	**Slope**	**P-value**
Intercept	-1.924	0.000E+00
Number iHS data points	0.075	5.070E-03
Density of conserved elements	0.183	4.455E-04
Recombination rate	-2.127	0.000E+00
Number PPIs	-0.038	2.921E-01
Regulatory density (ChIP-seq)	0.162	1.474E-01
Distance to VIPs	-0.216	2.541E-08
Gene number	-0.335	1.595E-04
Coding density	0.299	1.320E-03
Gene length	0.018	6.375E-01
Regulatory density in immune cells (ChIP-seq)	0.174	3.061E-02

**Covariate**	**Slope**	**P-value**
Gene expression	-0.182	1.284E-02
Gene expression in testis	0.034	4.919E-01
Gene expression in immune cells	0.376	2.216E-08
Regulatory density in testis (ChIP-seq)	-0.489	0.000E+00
Regulatory density (DNaseI)	-0.270	4.698E-02
GC-content	0.097	3.703E-01

Utah residents 1000kb

Figure S10: Mixture of Gaussian distributions fitting observed iHS (1000kb windows) for Utah residents. The figure shows the two Gaussian distributions, component 1 and 2 of iHS, being the latter enriched in positive selection. In that component, iHS linearly depends on the genomic factors considered. The figure shows iHS after log transformation and scaling (see Methods). Legend: Light blue = Observed iHS; Dark blue = Mixture model; Full red curve = Component 1 of the mixture model; Dashed red curve = Component 2 of the mixture model enriched in positive selection.

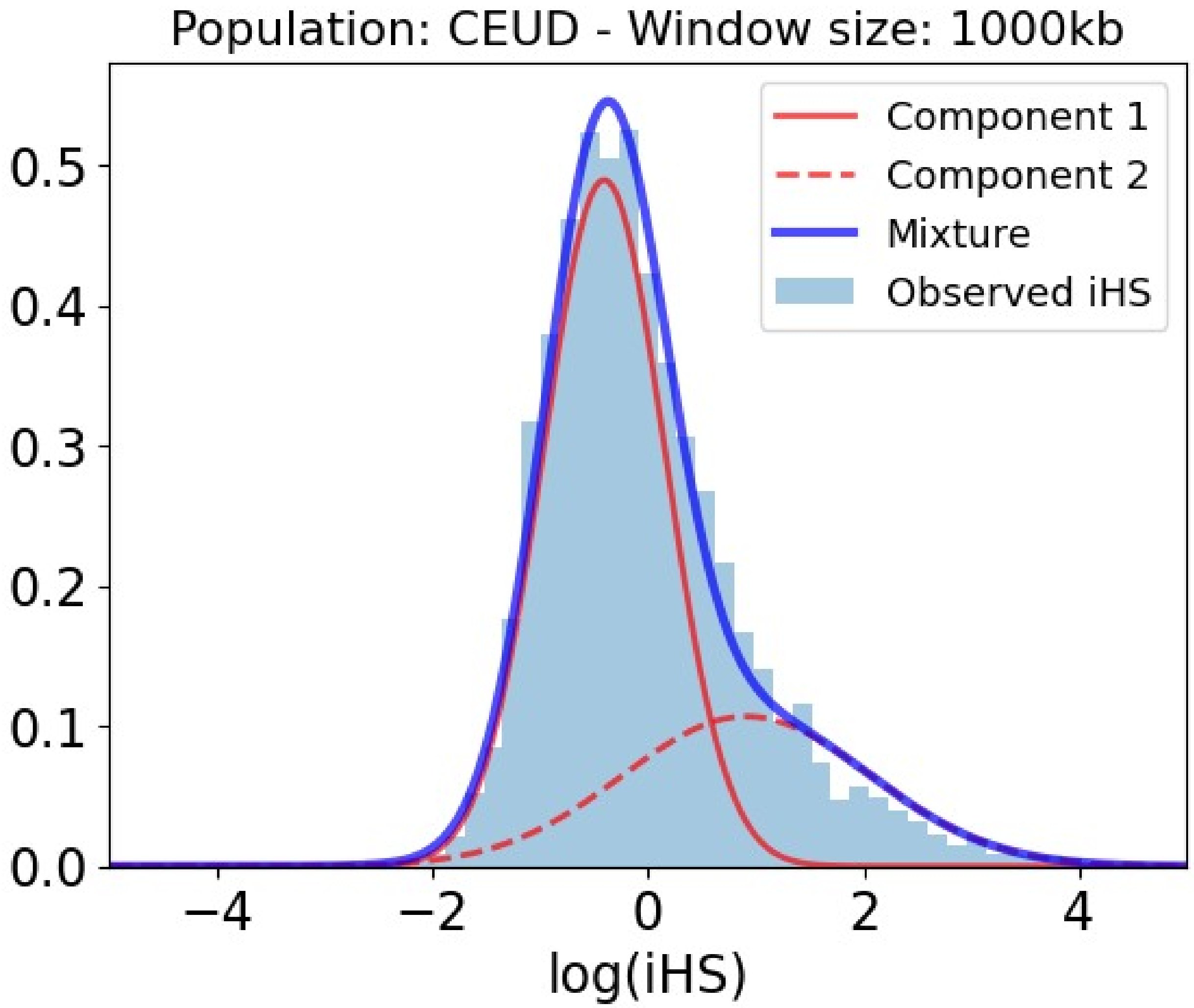

Table S10: Slopes and p-values of the association between iHS and genomic factors for the Utah residents population in 1000kb within the selection-enriched component.

**Covariate**	**Slope**	**P-value**
Intercept	-1.766	0.000E+00
Number iHS data points	0.315	0.000E+00
Density of conserved elements	0.379	7.738E-13
Recombination rate	-2.798	0.000E+00
Number PPIs	-0.050	1.951E-01
Regulatory density (ChIP-seq)	0.593	3.450E-06
Distance to VIPs	-0.337	8.449E-14
Gene number	-0.521	1.284E-06
Coding density	0.535	8.716E-06
Gene length	-0.047	2.840E-01
Regulatory density in immune cells (ChIP-seq)	-0.051	6.010E-01

Covariate	Slope	P-value
Gene expression	-0.335	9.695E-06
Gene expression in testis	-0.031	5.450E-01
Gene expression in immune cells	0.408	6.868E-09
Regulatory density in testis (ChIP-seq)	-1.031	0.000E+00
Regulatory density (DNaseI)	-0.609	5.447E-05
GC-content	0.745	1.350E-08

Toscani

Toscani 50kb

Figure S11: Mixture of Gaussian distributions fitting observed iHS (50kb windows) for Toscani. The figure shows the two Gaussian distributions, component 1 and 2 of iHS, being the latter enriched in positive selection. In that component, iHS linearly depends on the genomic factors considered. The figure shows iHS after log transformation and scaling (see Methods). Legend: Light blue = Observed iHS; Dark blue = Mixture model; Full red curve = Component 1 of the mixture model; Dashed red curve = Component 2 of the mixture model enriched in positive selection.

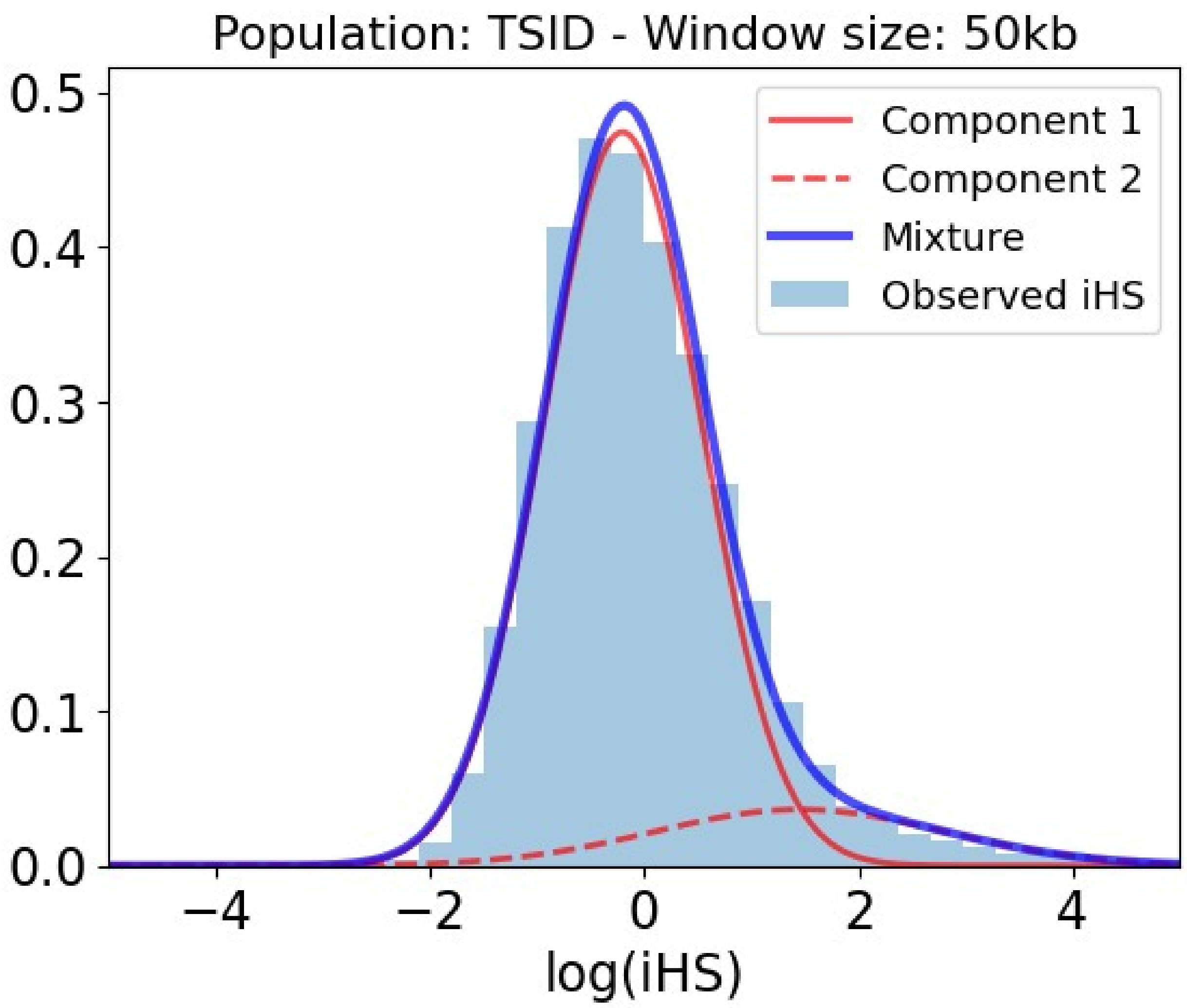

Table S11: Slopes and p-values of the association between iHS and genomic factors for the Toscani population in 50kb within the selection-enriched component.

**Covariate**	**Slope**	**P-value**
Intercept	-107.704	0.000E+00
Number iHS data points	1.332	0.000E+00
Density of conserved elements	0.016	9.181E-01
Recombination rate	-168.545	0.000E+00
Number PPIs	0.266	3.654E-03
Regulatory density (ChIP-seq)	0.667	3.454E-02
Distance to VIPs	-0.088	2.359E-01
Gene number	0.171	2.920E-01
Coding density	0.129	4.685E-01
Gene length	0.136	2.698E-01
Regulatory density in immune cells (ChIP-seq)	-0.909	5.142E-03

**Covariate**	**Slope**	**P-value**
Gene expression	0.151	5.291E-01
Gene expression in testis	0.363	1.632E-02
Gene expression in immune cells	0.194	4.504E-01
Regulatory density in testis (ChIP-seq)	0.072	6.815E-01
Regulatory density (DNaseI)	-0.310	3.124E-01
GC-content	-0.078	7.724E-01

Toscani 100kb

Figure S12: Mixture of Gaussian distributions fitting observed iHS (100kb windows) for Toscani. The figure shows the two Gaussian distributions, component 1 and 2 of iHS, being the latter enriched in positive selection. In that component, iHS linearly depends on the genomic factors considered. The figure shows iHS after log transformation and scaling (see Methods). Legend: Light blue = Observed iHS; Dark blue = Mixture model; Full red curve = Component 1 of the mixture model; Dashed red curve = Component 2 of the mixture model enriched in positive selection.

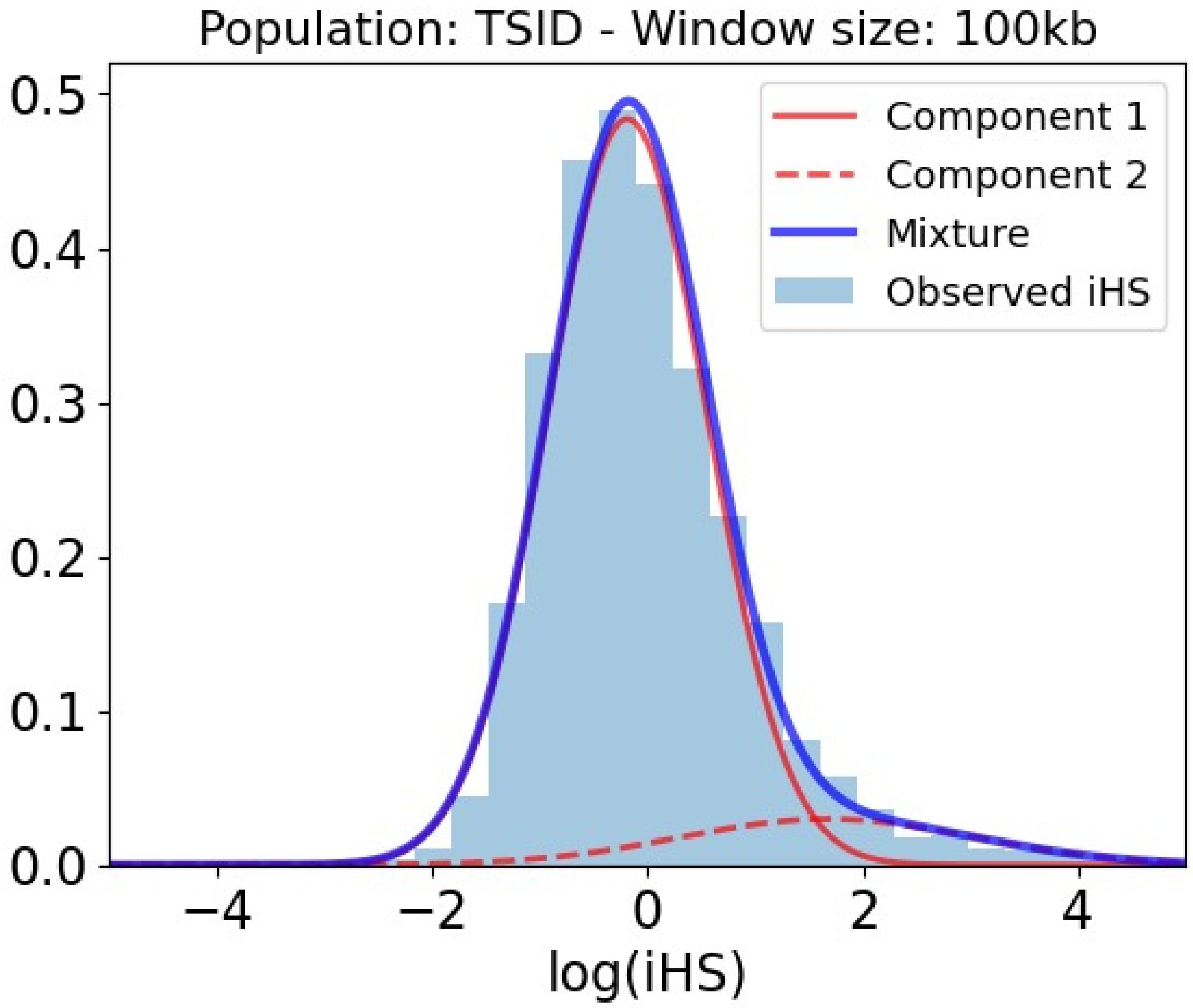

Table S12: Slopes and p-values of the association between iHS and genomic factors for the Toscani population in 100kb within the selection-enriched component.

**Covariate**	**Slope**	**P-value**
Intercept	-23.200	0.000E+00
Number iHS data points	0.499	3.463E-12
Density of conserved elements	0.143	2.029E-01
Recombination rate	-29.339	0.000E+00
Number PPIs	0.153	9.134E-03
Regulatory density (ChIP-seq)	0.131	4.527E-01
Distance to VIPs	-0.065	2.178E-01
Gene number	0.024	8.024E-01
Coding density	-0.014	8.975E-01
Gene length	0.108	1.694E-01
Regulatory density in immune cells (ChIP-seq)	-0.143	4.497E-01

Covariate	Slope	P-value
Gene expression	-0.154	3.359E-01
Gene expression in testis	0.253	9.575E-03
Gene expression in immune cells	0.387	9.657E-03
Regulatory density in testis (ChIP-seq)	-0.134	1.789E-01
Regulatory density (DNaseI)	-0.107	5.990E-01
GC-content	-0.131	4.052E-01

Toscani 200kb

Figure S13: Mixture of Gaussian distributions fitting observed iHS (200kb windows) for Toscani. The figure shows the two Gaussian distributions, component 1 and 2 of iHS, being the latter enriched in positive selection. In that component, iHS linearly depends on the genomic factors considered. The figure shows iHS after log transformation and scaling (see Methods). Legend: Light blue = Observed iHS; Dark blue = Mixture model; Full red curve = Component 1 of the mixture model; Dashed red curve = Component 2 of the mixture model enriched in positive selection.

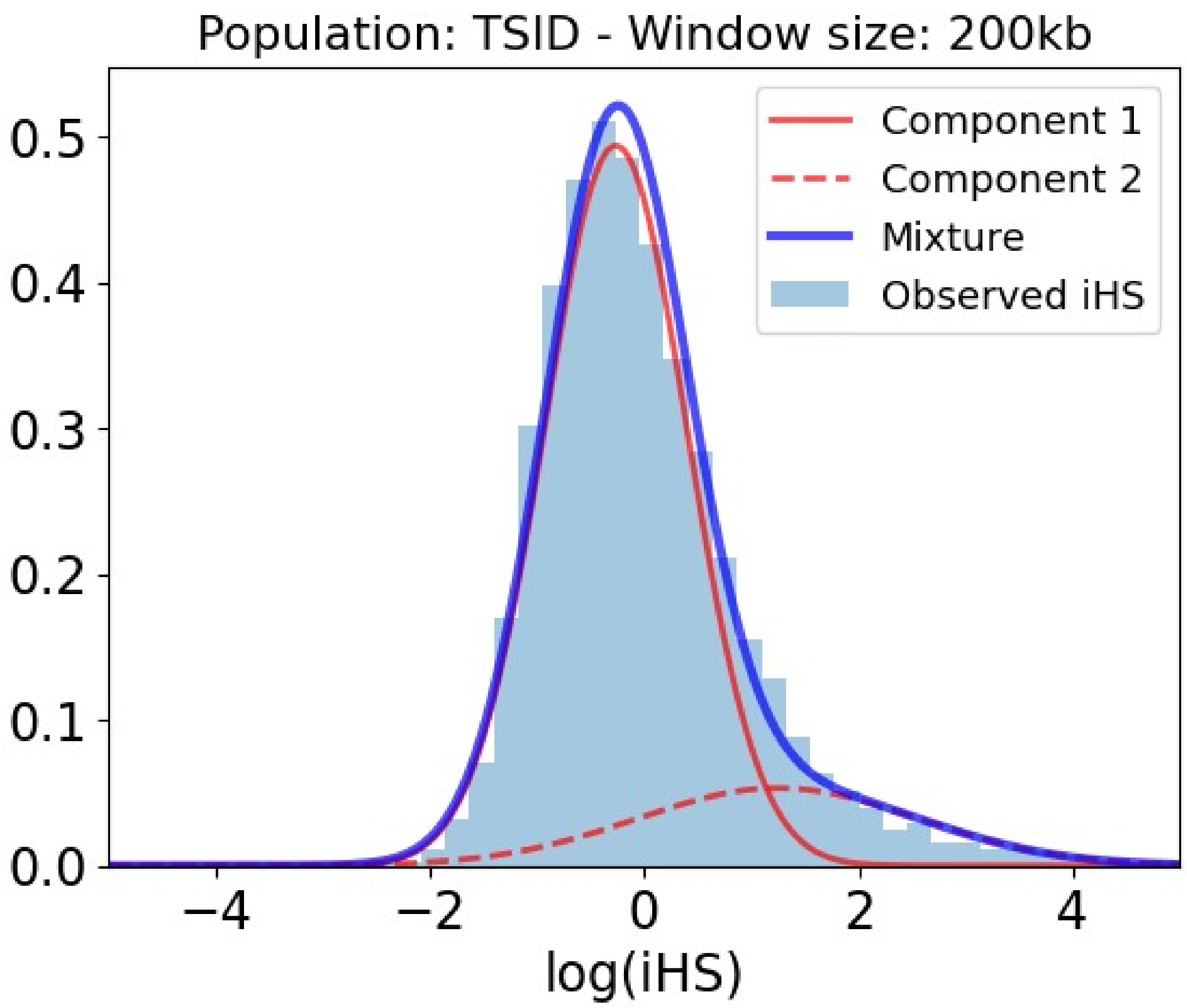

Table S13: Slopes and p-values of the association between iHS and genomic factors for the Toscani population in 200kb within the selection-enriched component.

**Covariate**	**Slope**	**P-value**
Intercept	-2.868	0.000E+00
Number iHS data points	0.073	1.139E-02
Density of conserved elements	0.182	2.434E-03
Recombination rate	-2.571	0.000E+00
Number PPIs	0.054	1.503E-01
Regulatory density (ChIP-seq)	0.164	1.165E-01
Distance to VIPs	-0.154	1.622E-04
Gene number	-0.112	1.334E-01
Coding density	0.180	2.083E-02
Gene length	0.089	3.656E-02
Regulatory density in immune cells (ChIP-seq)	-0.186	4.183E-02

Covariate	Slope	P-value
Gene expression	-0.355	6.950E-05
Gene expression in testis	0.260	1.088E-05
Gene expression in immune cells	0.526	1.051E-10
Regulatory density in testis (ChIP-seq)	0.071	2.765E-01
Regulatory density (DNaseI)	-0.188	1.610E-01
GC-content	-0.339	1.435E-03

Toscani 500kb

Figure S14: Mixture of Gaussian distributions fitting observed iHS (500kb windows) for Toscani. The figure shows the two Gaussian distributions, component 1 and 2 of iHS, being the latter enriched in positive selection. In that component, iHS linearly depends on the genomic factors considered. The figure shows iHS after log transformation and scaling (see Methods). Legend: Light blue = Observed iHS; Dark blue = Mixture model; Full red curve = Component 1 of the mixture model; Dashed red curve = Component 2 of the mixture model enriched in positive selection.

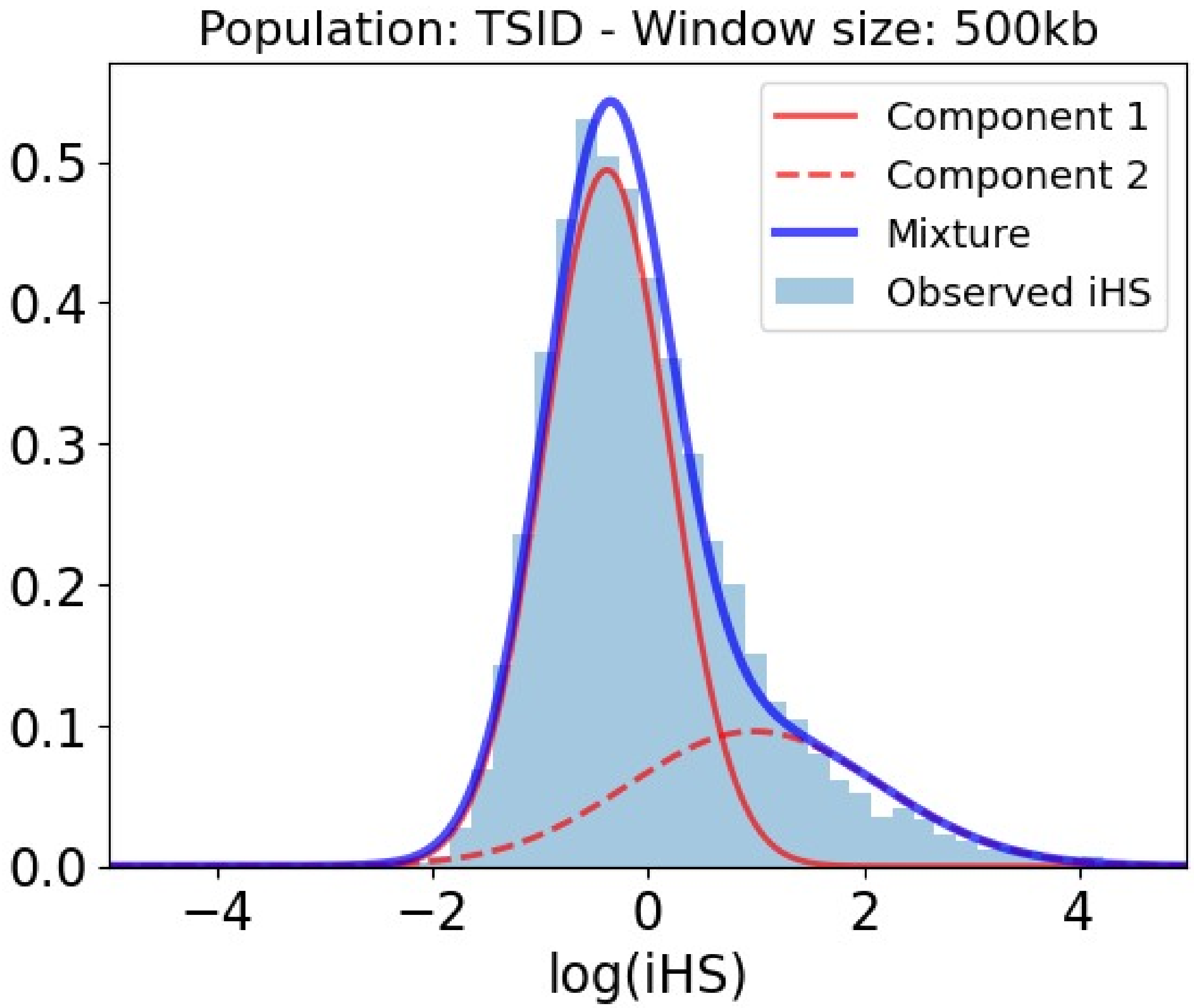

Table S14: Slopes and p-values of the association between iHS and genomic factors for the Toscani population in 500kb within the selection-enriched component.

**Covariate**	**Slope**	**P-value**
Intercept	-1.679	0.000E+00
Number iHS data points	0.092	4.913E-04
Density of conserved elements	0.213	1.402E-05
Recombination rate	-2.025	0.000E+00
Number PPIs	-0.024	4.767E-01
Regulatory density (ChIP-seq)	0.463	2.710E-06
Distance to VIPs	-0.154	4.266E-05
Gene number	-0.199	1.038E-02
Coding density	0.344	2.734E-05
Gene length	0.004	9.115E-01
Regulatory density in immune cells (ChIP-seq)	0.121	1.064E-01

Covariate	Slope	P-value
Gene expression	-0.182	8.404E-03
Gene expression in testis	0.066	1.686E-01
Gene expression in immune cells	0.333	2.317E-07
Regulatory density in testis (ChIP-seq)	-0.444	3.275E-14
Regulatory density (DNaseI)	-0.324	1.123E-02
GC-content	-0.138	1.985E-01

Toscani 1000kb

Figure S15: Mixture of Gaussian distributions fitting observed iHS (1000kb windows) for Toscani. The figure shows the two Gaussian distributions, component 1 and 2 of iHS, being the latter enriched in positive selection. In that component, iHS linearly depends on the genomic factors considered. The figure shows iHS after log transformation and scaling (see Methods). Legend: Light blue = Observed iHS; Dark blue = Mixture model; Full red curve = Component 1 of the mixture model; Dashed red curve = Component 2 of the mixture model enriched in positive selection.

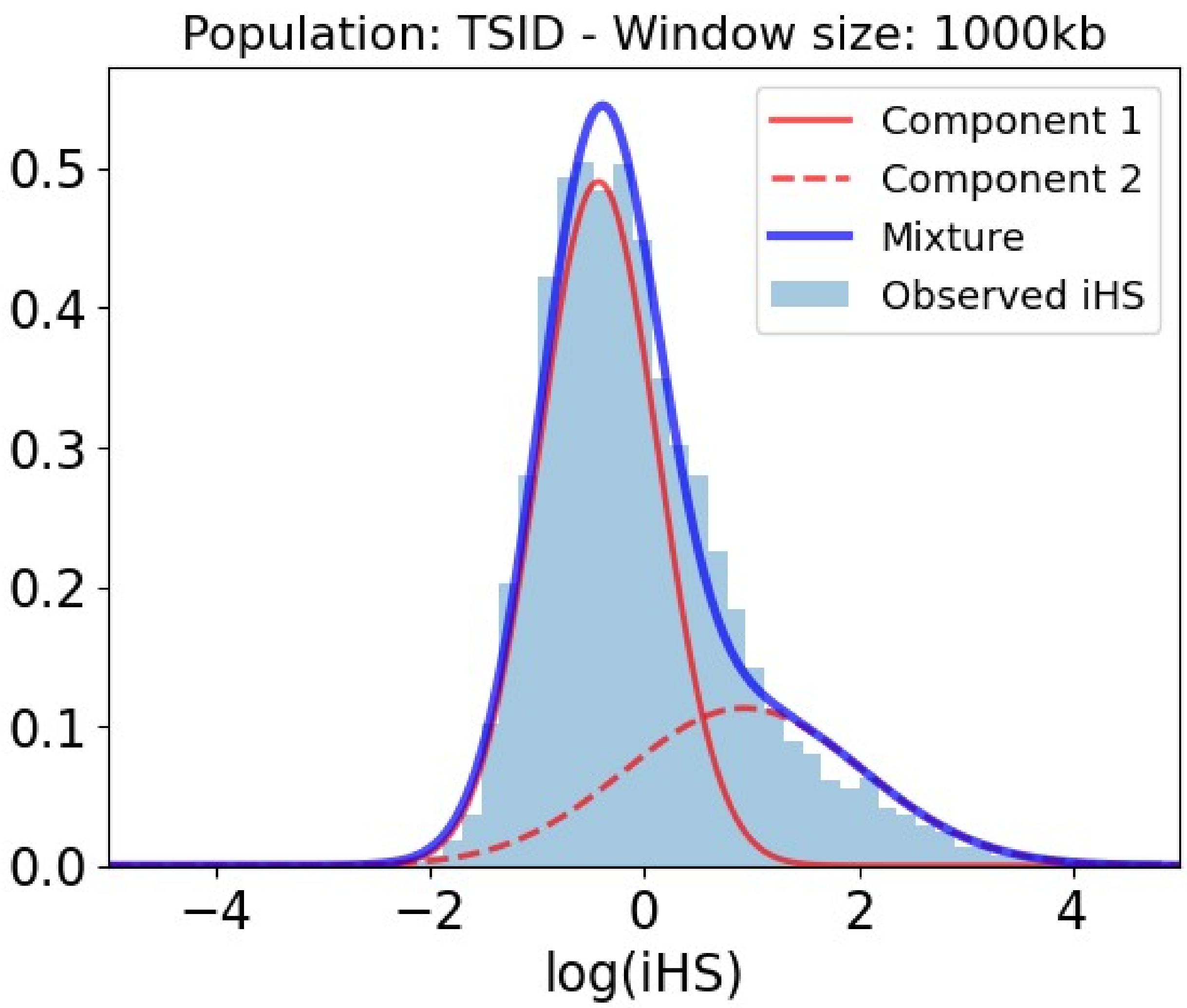

Table S15: Slopes and p-values of the association between iHS and genomic factors for the Toscani population in 1000kb within the selection-enriched component.

Covariate	Slope	P-value
Intercept	-1.660	0.000E+00
Number iHS data points	0.330	0.000E+00
Density of conserved elements	0.337	2.701E-11
Recombination rate	-2.696	0.000E+00
Number PPIs	-0.041	2.671E-01
Regulatory density (ChIP-seq)	0.742	3.063E-10
Distance to VIPs	-0.266	1.917E-10
Gene number	-0.397	5.912E-05
Coding density	0.586	3.772E-08
Gene length	-0.024	5.588E-01
Regulatory density in immune cells (ChIP-seq)	-0.079	3.944E-01

**Covariate**	**Slope**	**P-value**
Gene expression	-0.253	4.915E-04
Gene expression in testis	-0.040	4.213E-01
Gene expression in immune cells	0.366	5.339E-08
Regulatory density in testis (ChIP-seq)	-0.861	0.000E+00
Regulatory density (DNaseI)	-0.635	8.237E-06
GC-content	0.516	2.469E-05

Han Chinese

Han Chinese 50kb

Figure S16: Mixture of Gaussian distributions fitting observed iHS (50kb windows) for Han Chinese. The figure shows the two Gaussian distributions, component 1 and 2 of iHS, being the latter enriched in positive selection. In that component, iHS linearly depends on the genomic factors considered. The figure shows iHS after log transformation and scaling (see Methods). Legend: Light blue = Observed iHS; Dark blue = Mixture model; Full red curve = Component 1 of the mixture model; Dashed red curve = Component 2 of the mixture model enriched in positive selection.

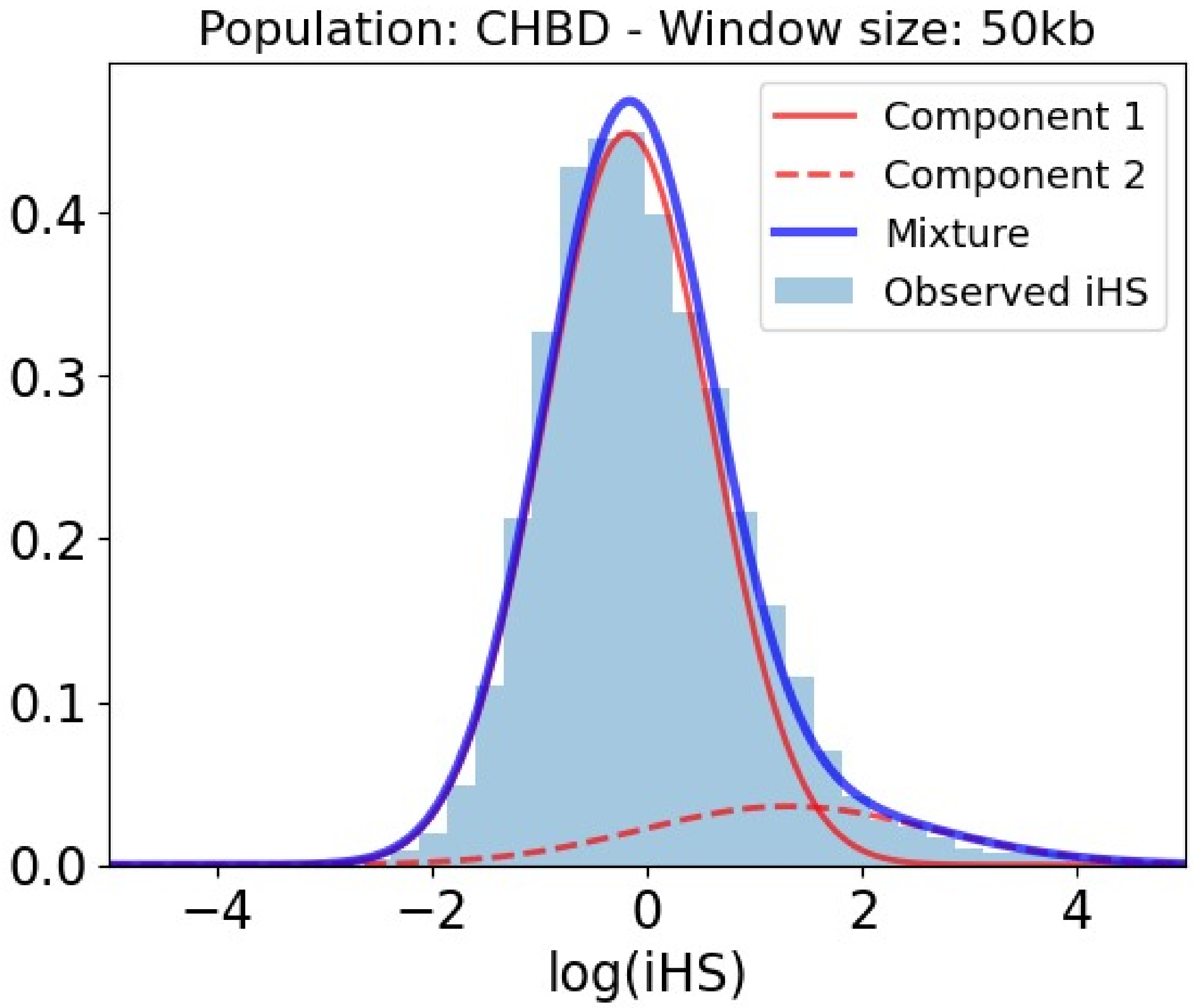

Table S16: Slopes and p-values of the association between iHS and genomic factors for the Han Chinese population in 50kb within the selection-enriched component.

Covariate	Slope	P-value
Intercept	-92.249	0.000E+00
Number iHS data points	1.219	0.000E+00
Density of conserved elements	-0.122	4.844E-01
Recombination rate	-144.576	0.000E+00
Number PPIs	0.075	4.238E-01
Regulatory density (ChIP-seq)	0.320	1.732E-01
Distance to VIPs	-0.108	9.944E-02
Gene number	0.119	3.918E-01
Coding density	0.258	1.073E-01
Gene length	0.907	2.363E-06
Regulatory density in immune cells (ChIP-seq)	-0.999	1.356E-04

Covariate	Slope	P-value
Gene expression	-0.594	2.694E-02
Gene expression in testis	0.204	2.022E-01
Gene expression in immune cells	0.855	8.659E-04
Regulatory density in testis (ChIP-seq)	0.004	9.712E-01
Regulatory density (DNaseI)	0.261	4.141E-01
GC-content	0.033	9.028E-01

Han Chinese 100kb

Figure S17: Mixture of Gaussian distributions fitting observed iHS (100kb windows) for Han Chinese. The figure shows the two Gaussian distributions, component 1 and 2 of iHS, being the latter enriched in positive selection. In that component, iHS linearly depends on the genomic factors considered. The figure shows iHS after log transformation and scaling (see Methods). Legend: Light blue = Observed iHS; Dark blue = Mixture model; Full red curve = Component 1 of the mixture model; Dashed red curve = Component 2 of the mixture model enriched in positive selection.

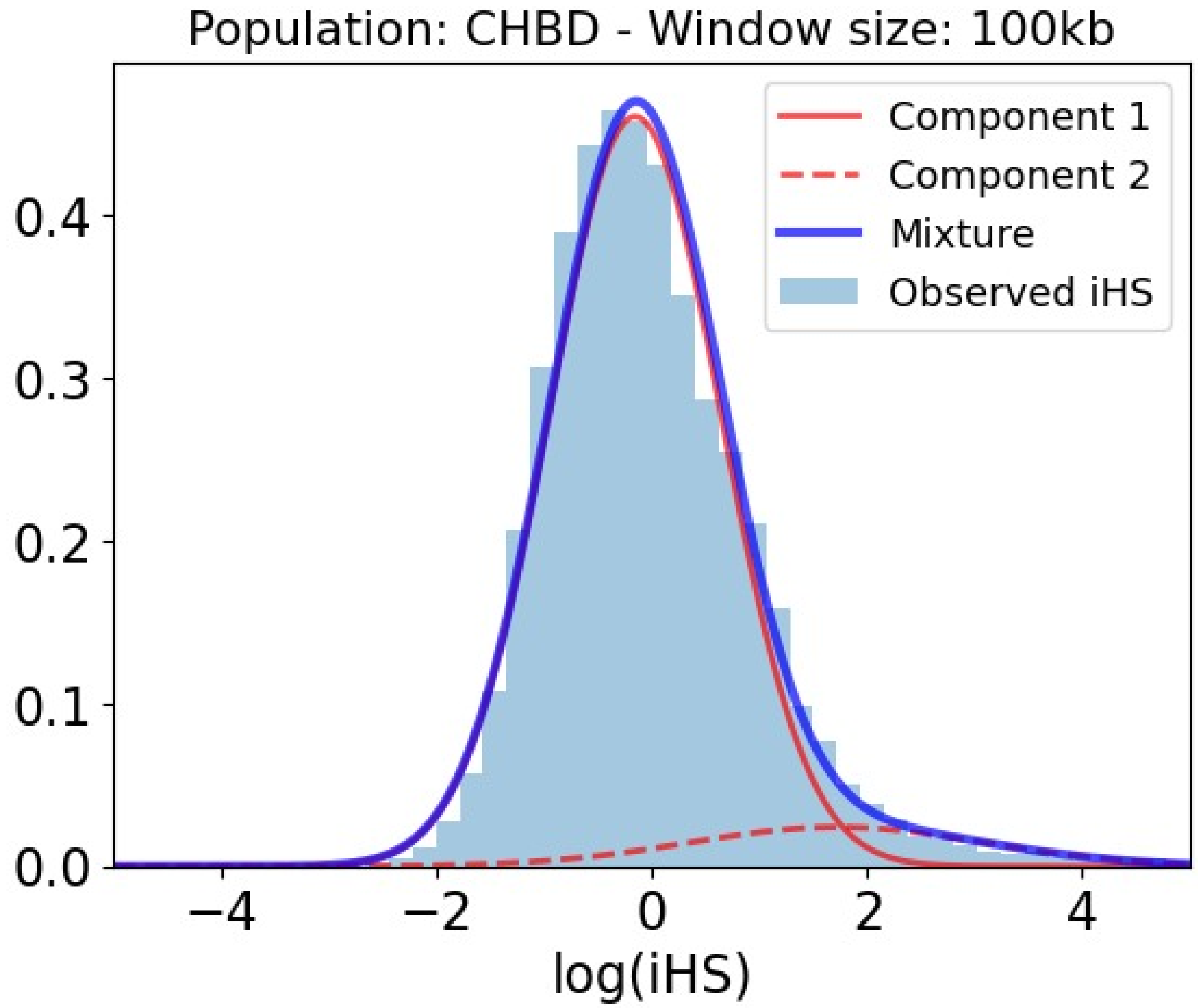

Table S17: Slopes and p-values of the association between iHS and genomic factors for the Han Chinese population in 100kb within the selection-enriched component.

Covariate	Slope	P-value
Intercept	-58.905	0.000E+00
Number iHS data points	1.538	0.000E+00
Density of conserved elements	0.085	6.065E-01
Recombination rate	-74.503	0.000E+00
Number PPIs	0.137	8.902E-02
Regulatory density (ChIP-seq)	0.490	8.604E-02
Distance to VIPs	-0.048	5.595E-01
Gene number	0.466	2.526E-03
Coding density	-0.160	3.587E-01
Gene length	0.585	5.204E-04
Regulatory density in immune cells (ChIP-seq)	-0.768	5.987E-03

Covariate	Slope	P-value
Gene expression	-0.345	1.618E-01
Gene expression in testis	0.275	5.362E-02
Gene expression in immune cells	0.701	1.941E-03
Regulatory density in testis (ChIP-seq)	0.114	3.893E-01
Regulatory density (DNaseI)	-0.041	9.046E-01
GC-content	-0.060	7.931E-01

Han Chinese 200kb

Figure S18: Mixture of Gaussian distributions fitting observed iHS (200kb windows) for Han Chinese. The figure shows the two Gaussian distributions, component 1 and 2 of iHS, being the latter enriched in positive selection. In that component, iHS linearly depends on the genomic factors considered. The figure shows iHS after log transformation and scaling (see Methods). Legend: Light blue = Observed iHS; Dark blue = Mixture model; Full red curve = Component 1 of the mixture model; Dashed red curve = Component 2 of the mixture model enriched in positive selection.

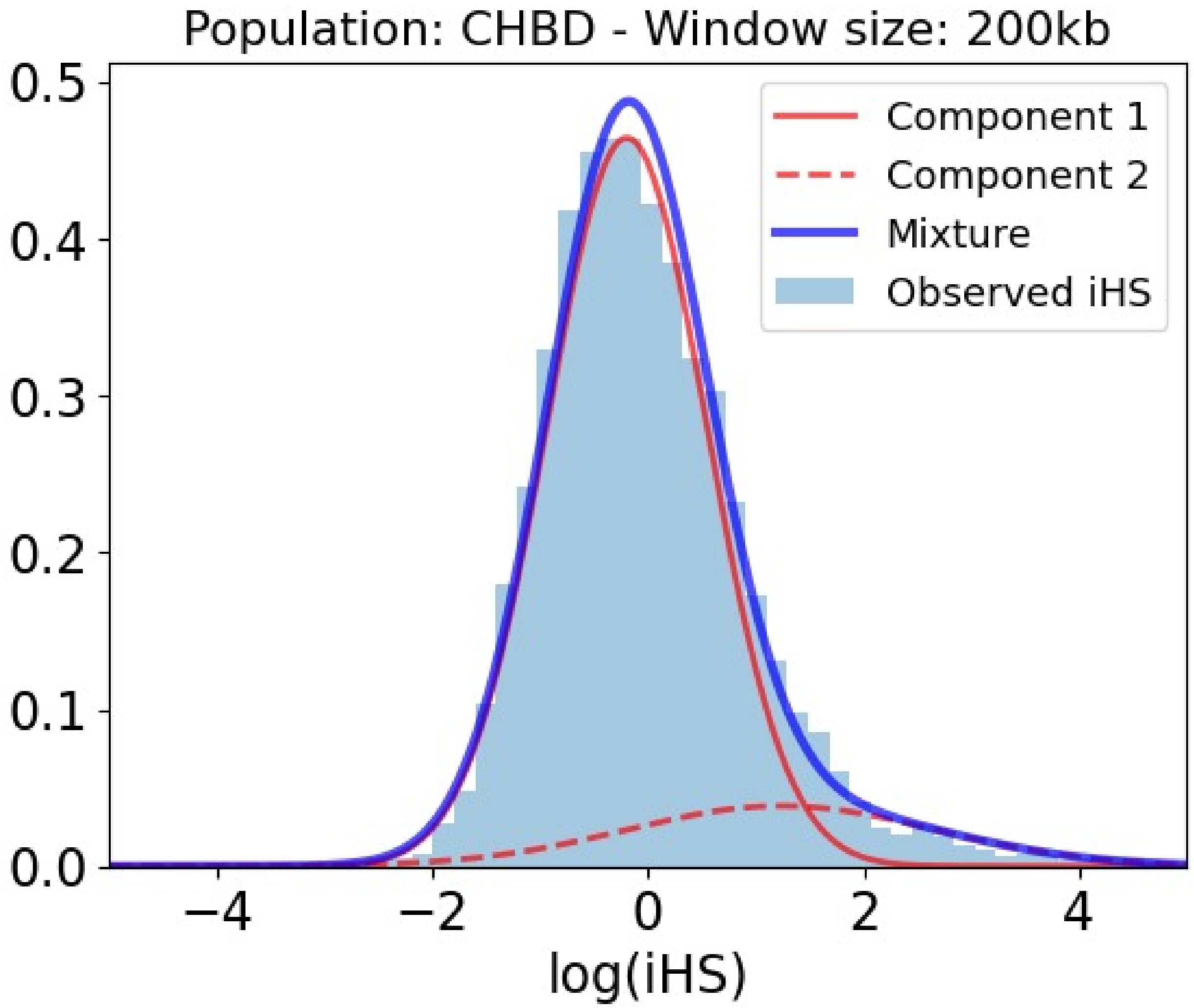

Table S18: Slopes and p-values of the association between iHS and genomic factors for the Han Chinese population in 200kb within the selection-enriched component.

**Covariate**	**Slope**	**P-value**
Intercept	-3.774	0.000E+00
Number iHS data points	0.143	7.126E-05
Density of conserved elements	0.115	1.193E-01
Recombination rate	-3.444	0.000E+00
Number PPIs	0.046	3.054E-01
Regulatory density (ChIP-seq)	0.170	2.119E-01
Distance to VIPs	-0.172	1.465E-03
Gene number	-0.001	9.979E-01
Coding density	0.251	8.256E-03
Gene length	0.196	4.565E-05
Regulatory density in immune cells (ChIP-seq)	-0.429	5.853E-04

Covariate	Slope	P-value
Gene expression	-0.453	4.496E-05
Gene expression in testis	0.207	3.392E-03
Gene expression in immune cells	0.623	9.617E-10
Regulatory density in testis (ChIP-seq)	0.112	2.204E-01
Regulatory density (DNaseI)	-0.283	9.546E-02
GC-content	-0.020	8.817E-01

Han Chinese 500kb

Figure S19: Mixture of Gaussian distributions fitting observed iHS (500kb windows) for Han Chinese. The figure shows the two Gaussian distributions, component 1 and 2 of iHS, being the latter enriched in positive selection. In that component, iHS linearly depends on the genomic factors considered. The figure shows iHS after log transformation and scaling (see Methods). Legend: Light blue = Observed iHS; Dark blue = Mixture model; Full red curve = Component 1 of the mixture model; Dashed red curve = Component 2 of the mixture model enriched in positive selection.

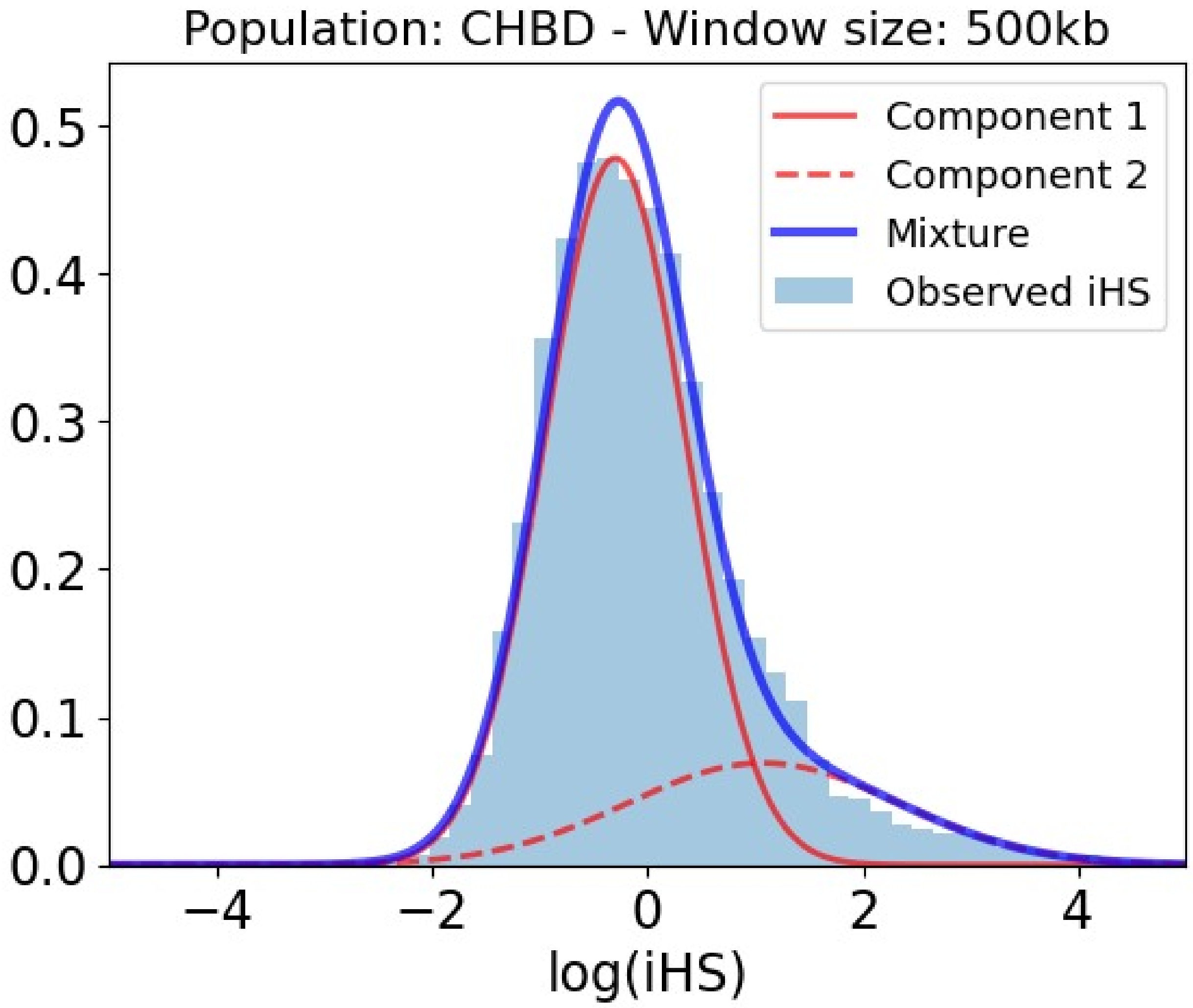

Table S19: Slopes and p-values of the association between iHS and genomic factors for the Han Chinese population in 500kb within the selection-enriched component.

Covariate	Slope	P-value
Intercept	-2.146	0.000E+00
Number iHS data points	0.141	5.436E-07
Density of conserved elements	0.196	2.236E-04
Recombination rate	-2.191	0.000E+00
Number PPIs	-0.044	2.385E-01
Regulatory density (ChIP-seq)	0.294	1.344E-02
Distance to VIPs	-0.156	2.071E-04
Gene number	-0.128	1.567E-01
Coding density	0.201	3.041E-02
Gene length	0.070	7.385E-02
Regulatory density in immune cells (ChIP-seq)	-0.158	9.246E-02

Covariate	Slope	P-value
Gene expression	-0.314	5.479E-05
Gene expression in testis	0.117	2.815E-02
Gene expression in immune cells	0.372	2.474E-07
Regulatory density in testis (ChIP-seq)	-0.123	5.385E-02
Regulatory density (DNaseI)	-0.415	4.798E-03
GC-content	0.341	4.762E-03

Han Chinese 1000kb

Figure S20: Mixture of Gaussian distributions fitting observed iHS (1000kb windows) for Han Chinese. The figure shows the two Gaussian distributions, component 1 and 2 of iHS, being the latter enriched in positive selection. In that component, iHS linearly depends on the genomic factors considered. The figure shows iHS after log transformation and scaling (see Methods). Legend: Light blue = Observed iHS; Dark blue = Mixture model; Full red curve = Component 1 of the mixture model; Dashed red curve = Component 2 of the mixture model enriched in positive selection.

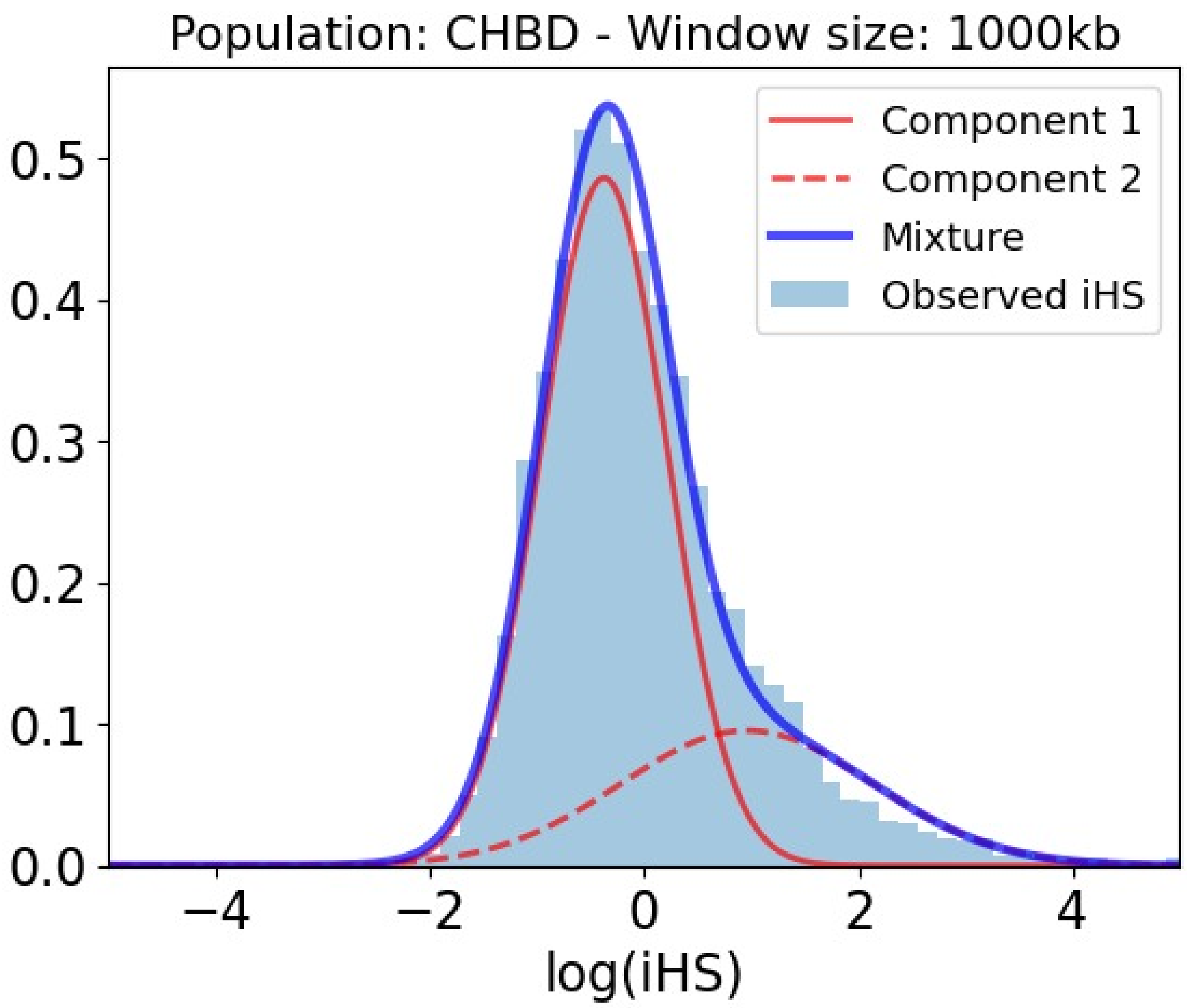

Table S20: Slopes and p-values of the association between iHS and genomic factors for the Han Chinese population in 1000kb within the selection-enriched component.

**Covariate**	**Slope**	**P-value**
Intercept	-1.778	0.000E+00
Number iHS data points	0.157	7.292E-08
Density of conserved elements	0.311	4.199E-10
Recombination rate	-2.510	0.000E+00
Number PPIs	-0.088	1.925E-02
Regulatory density (ChIP-seq)	0.490	3.921E-05
Distance to VIPs	-0.142	5.348E-04
Gene number	-0.460	6.230E-04
Coding density	0.396	1.928E-03
Gene length	0.004	9.125E-01
Regulatory density in immune cells (ChIP-seq)	-0.160	1.258E-01

Covariate	Slope	P-value
Gene expression	-0.245	1.135E-03
Gene expression in testis	0.065	2.056E-01
Gene expression in immune cells	0.307	9.507E-06
Regulatory density in testis (ChIP-seq)	-0.393	1.650E-09
Regulatory density (DNaseI)	-0.838	1.660E-08
GC-content	1.171	0.000E+00

Peruvians

Peruvians 50kb

Figure S21: Mixture of Gaussian distributions fitting observed iHS (50kb windows) for Peruvians. The figure shows the two Gaussian distributions, component 1 and 2 of iHS, being the latter enriched in positive selection. In that component, iHS linearly depends on the genomic factors considered. The figure shows iHS after log transformation and scaling (see Methods). Legend: Light blue = Observed iHS; Dark blue = Mixture model; Full red curve = Component 1 of the mixture model; Dashed red curve = Component 2 of the mixture model enriched in positive selection.

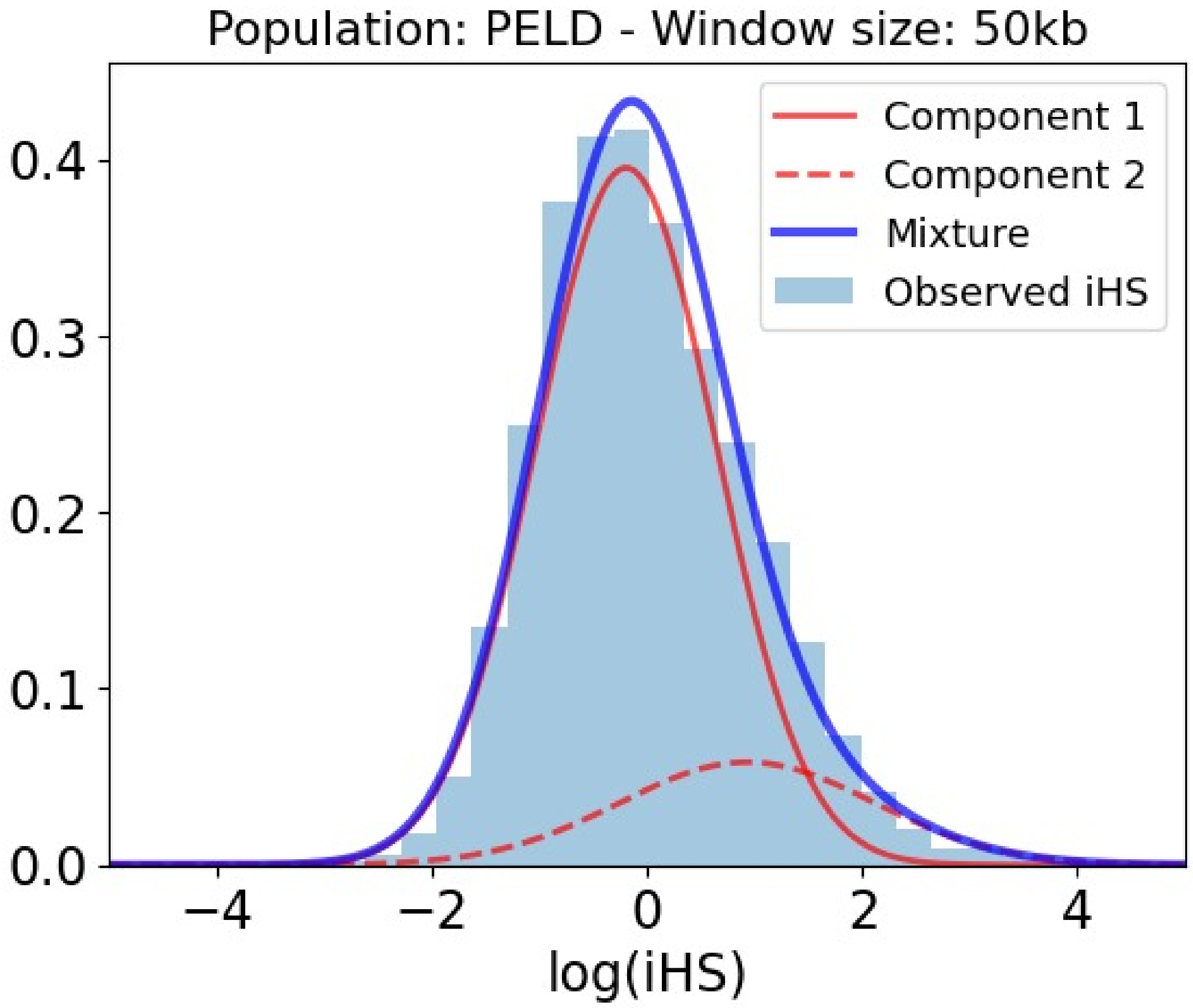

Table S21: Slopes and p-values of the association between iHS and genomic factors for the Peruvians population in 50kb within the selection-enriched component.

**Covariate**	**Slope**	**P-value**
Intercept	-36.485	0.000E+00
Number iHS data points	0.648	6.796E-09
Density of conserved elements	-0.443	7.655E-03
Recombination rate	-58.880	0.000E+00
Number PPIs	0.229	7.376E-03
Regulatory density (ChIP-seq)	-0.059	7.992E-01
Distance to VIPs	-0.219	6.141E-03
Gene number	-0.077	3.939E-01
Coding density	0.068	6.490E-01
Gene length	0.423	1.041E-03
Regulatory density in immune cells (ChIP-seq)	0.062	7.900E-01

Covariate	Slope	P-value
Gene expression	-0.632	5.121E-03
Gene expression in testis	0.449	8.541E-04
Gene expression in immune cells	0.535	1.707E-02
Regulatory density in testis (ChIP-seq)	0.243	7.025E-02
Regulatory density (DNaseI)	0.391	1.777E-01
GC-content	-0.023	9.263E-01

Peruvians 100kb

Figure S22: Mixture of Gaussian distributions fitting observed iHS (100kb windows) for Peruvians. The figure shows the two Gaussian distributions, component 1 and 2 of iHS, being the latter enriched in positive selection. In that component, iHS linearly depends on the genomic factors considered. The figure shows iHS after log transformation and scaling (see Methods). Legend: Light blue = Observed iHS; Dark blue = Mixture model; Full red curve = Component 1 of the mixture model; Dashed red curve = Component 2 of the mixture model enriched in positive selection.

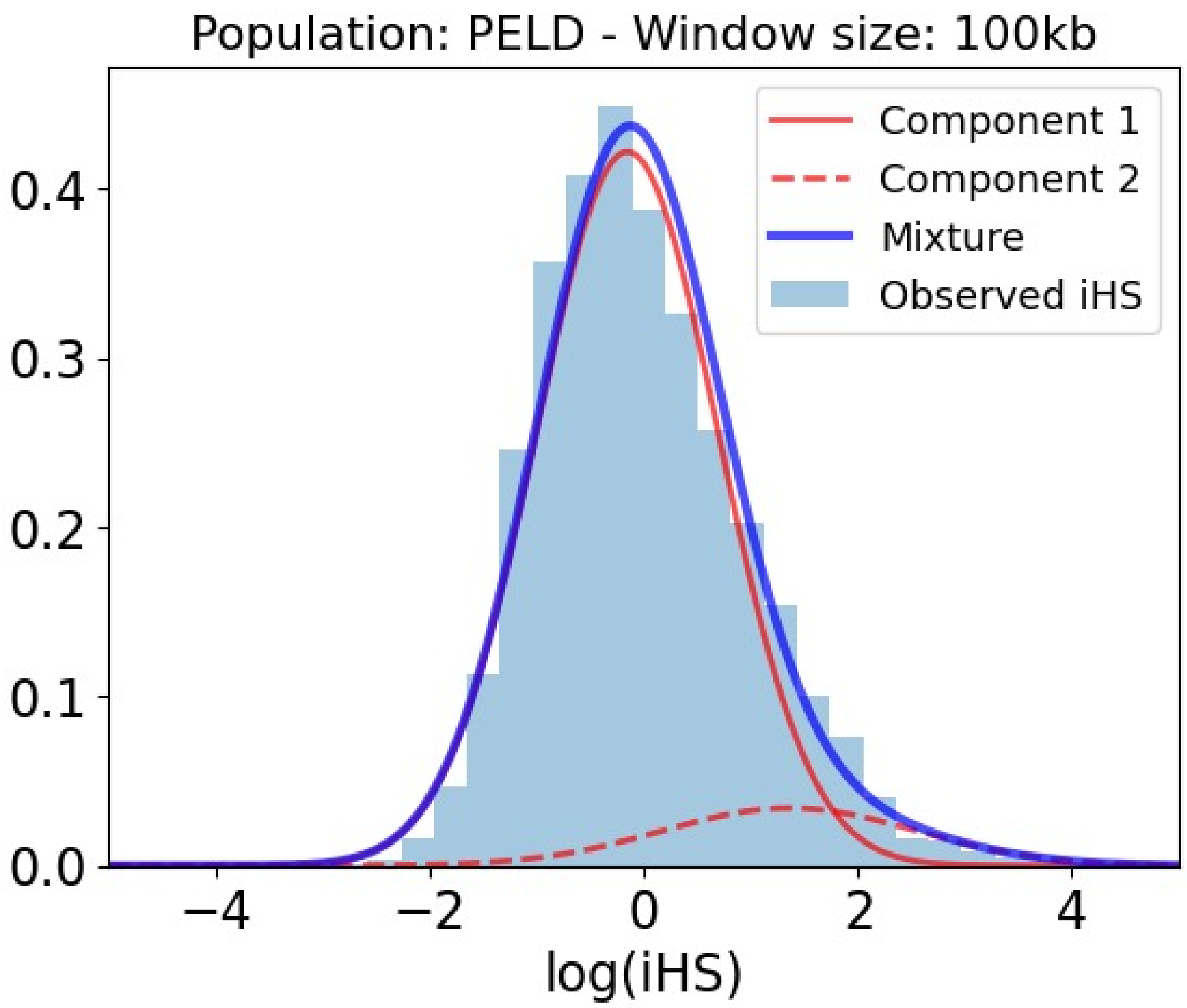

Table S22: Slopes and p-values of the association between iHS and genomic factors for the Peruvians population in 100kb within the selection-enriched component.

**Covariate**	**Slope**	**P-value**
Intercept	-46.010	0.000E+00
Number iHS data points	1.430	0.000E+00
Density of conserved elements	-0.050	7.650E-01
Recombination rate	-59.143	0.000E+00
Number PPIs	0.123	1.741E-01
Regulatory density (ChIP-seq)	-0.368	1.963E-01
Distance to VIPs	-0.183	4.899E-02
Gene number	0.247	1.160E-01
Coding density	-0.392	2.096E-02
Gene length	0.318	3.375E-03
Regulatory density in immune cells (ChIP-seq)	0.135	6.664E-01

Covariate	Slope	P-value
Gene expression	-0.128	5.601E-01
Gene expression in testis	0.313	3.456E-02
Gene expression in immune cells	0.340	1.265E-01
Regulatory density in testis (ChIP-seq)	0.190	1.681E-01
Regulatory density (DNaseI)	-0.102	7.513E-01
GC-content	0.294	2.438E-01

Peruvians 200kb

Figure S23: Mixture of Gaussian distributions fitting observed iHS (200kb windows) for Peruvians. The figure shows the two Gaussian distributions, component 1 and 2 of iHS, being the latter enriched in positive selection. In that component, iHS linearly depends on the genomic factors considered. The figure shows iHS after log transformation and scaling (see Methods). Legend: Light blue = Observed iHS; Dark blue = Mixture model; Full red curve = Component 1 of the mixture model; Dashed red curve = Component 2 of the mixture model enriched in positive selection.

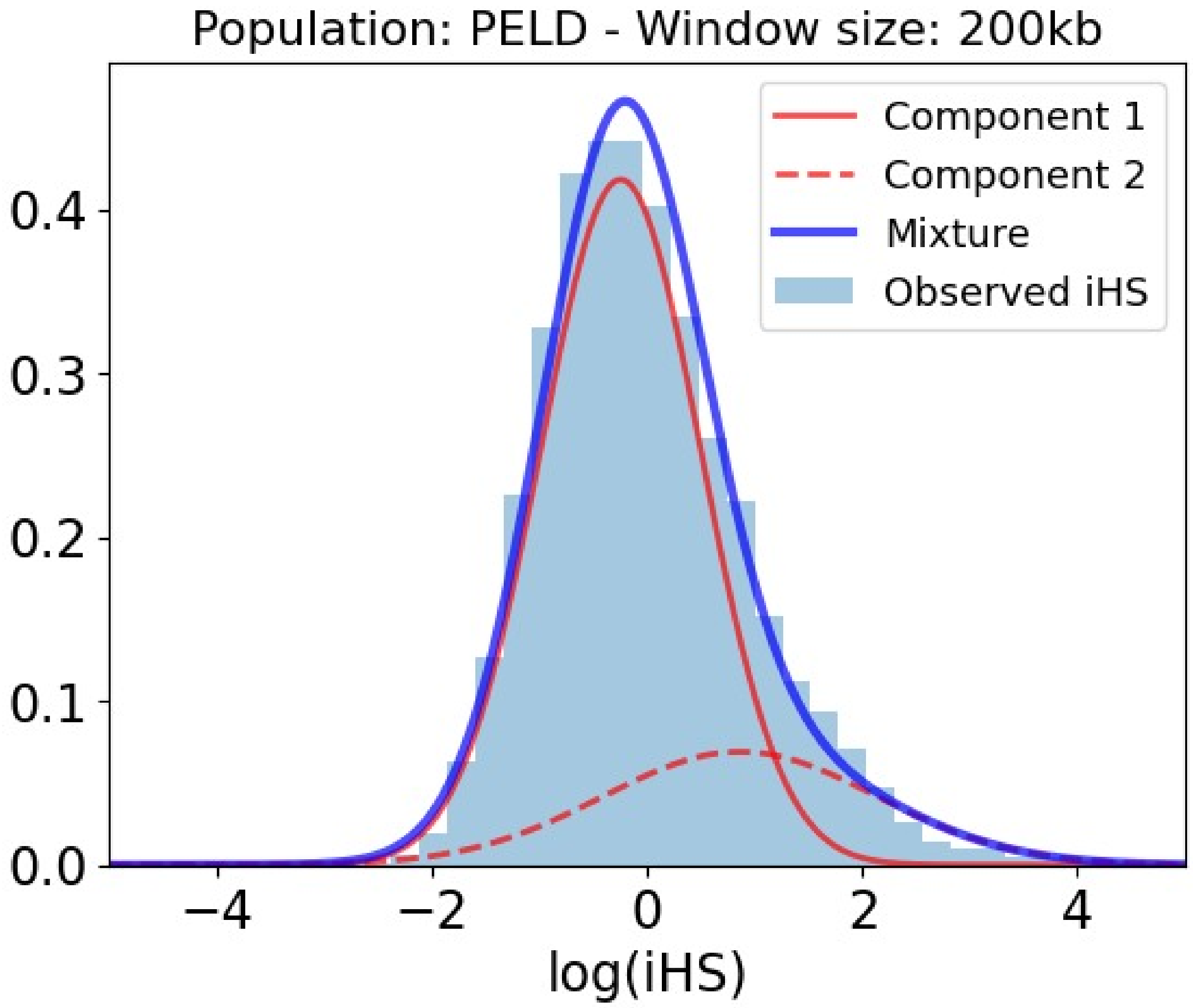

Table S23: Slopes and p-values of the association between iHS and genomic factors for the Peruvians population in 200kb within the selection-enriched component.

**Covariate**	**Slope**	**P-value**
Intercept	-2.553	0.000E+00
Number iHS data points	0.077	1.952E-02
Density of conserved elements	-0.216	2.002E-03
Recombination rate	-2.963	0.000E+00
Number PPIs	0.030	4.892E-01
Regulatory density (ChIP-seq)	0.134	2.563E-01
Distance to VIPs	-0.231	3.345E-05
Gene number	0.024	7.596E-01
Coding density	0.163	4.123E-02
Gene length	0.135	6.324E-03
Regulatory density in immune cells (ChIP-seq)	-0.122	2.385E-01

Covariate	Slope	P-value
Gene expression	-0.311	2.186E-03
Gene expression in testis	0.090	1.676E-01
Gene expression in immune cells	0.442	2.461E-06
Regulatory density in testis (ChIP-seq)	0.139	1.327E-02
Regulatory density (DNaseI)	-0.056	6.978E-01
GC-content	0.069	5.587E-01

Peruvians 500kb

Figure S24: Mixture of Gaussian distributions fitting observed iHS (500kb windows) for Peruvians. The figure shows the two Gaussian distributions, component 1 and 2 of iHS, being the latter enriched in positive selection. In that component, iHS linearly depends on the genomic factors considered. The figure shows iHS after log transformation and scaling (see Methods). Legend: Light blue = Observed iHS; Dark blue = Mixture model; Full red curve = Component 1 of the mixture model; Dashed red curve = Component 2 of the mixture model enriched in positive selection.

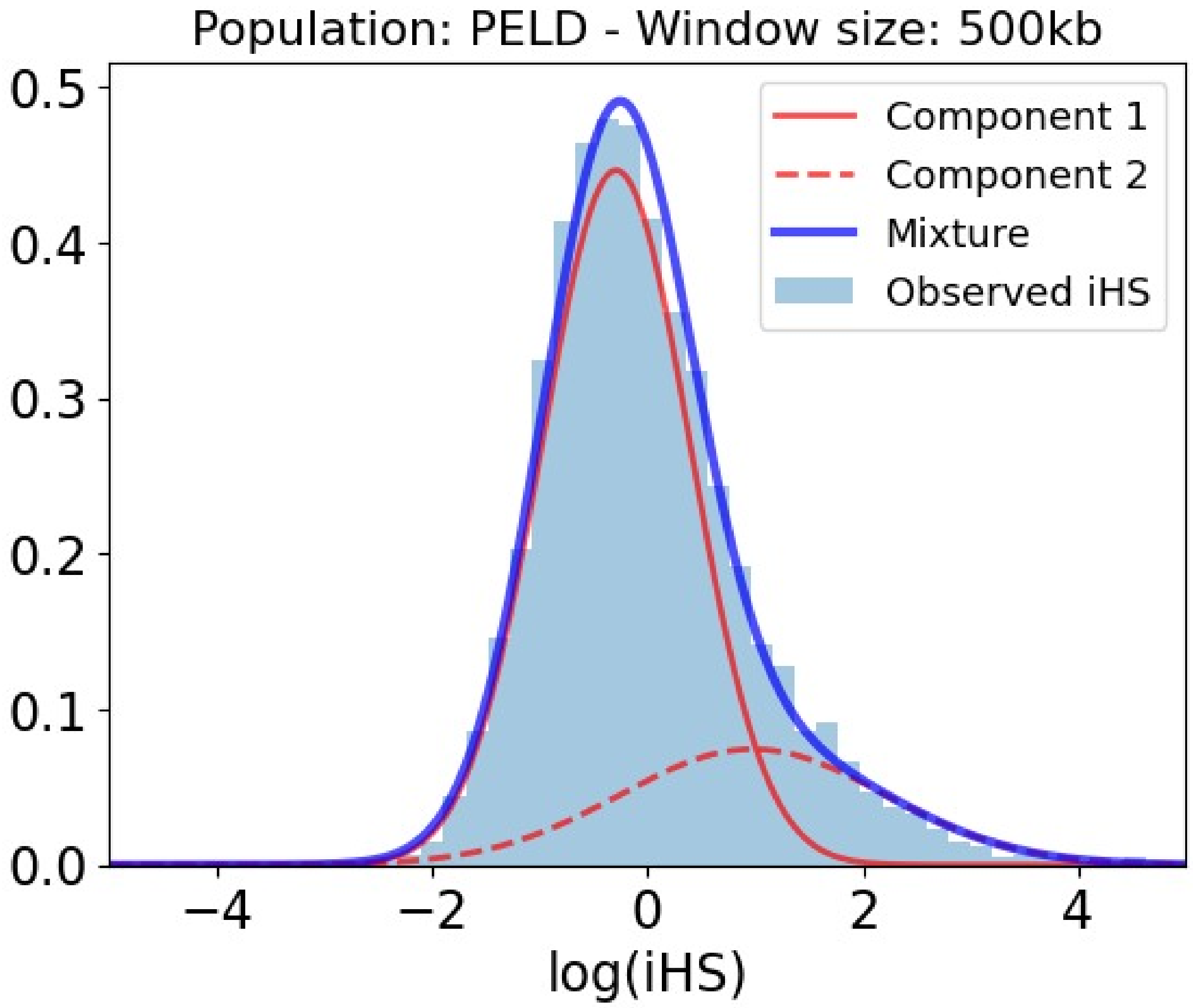

Table S24: Slopes and p-values of the association between iHS and genomic factors for the Peruvians population in 500kb within the selection-enriched component.

**Covariate**	**Slope**	**P-value**
Intercept	-1.970	0.000E+00
Number iHS data points	0.093	1.343E-03
Density of conserved elements	-0.137	1.499E-02
Recombination rate	-2.033	0.000E+00
Number PPIs	-0.040	3.115E-01
Regulatory density (ChIP-seq)	-0.044	7.231E-01
Distance to VIPs	-0.295	2.812E-07
Gene number	-0.072	3.523E-01
Coding density	0.216	8.404E-03
Gene length	0.077	8.007E-02
Regulatory density in immune cells (ChIP-seq)	0.221	1.628E-02

Covariate	Slope	P-value
Gene expression	-0.287	4.502E-04
Gene expression in testis	0.130	1.819E-02
Gene expression in immune cells	0.284	1.561E-04
Regulatory density in testis (ChIP-seq)	0.017	7.460E-01
Regulatory density (DNaseI)	-0.208	1.577E-01
GC-content	0.180	1.323E-01

Peruvians 1000kb

Figure S25: Mixture of Gaussian distributions fitting observed iHS (1000kb windows) for Peruvians. The figure shows the two Gaussian distributions, component 1 and 2 of iHS, being the latter enriched in positive selection. In that component, iHS linearly depends on the genomic factors considered. The figure shows iHS after log transformation and scaling (see Methods). Legend: Light blue = Observed iHS; Dark blue = Mixture model; Full red curve = Component 1 of the mixture model; Dashed red curve = Component 2 of the mixture model enriched in positive selection.

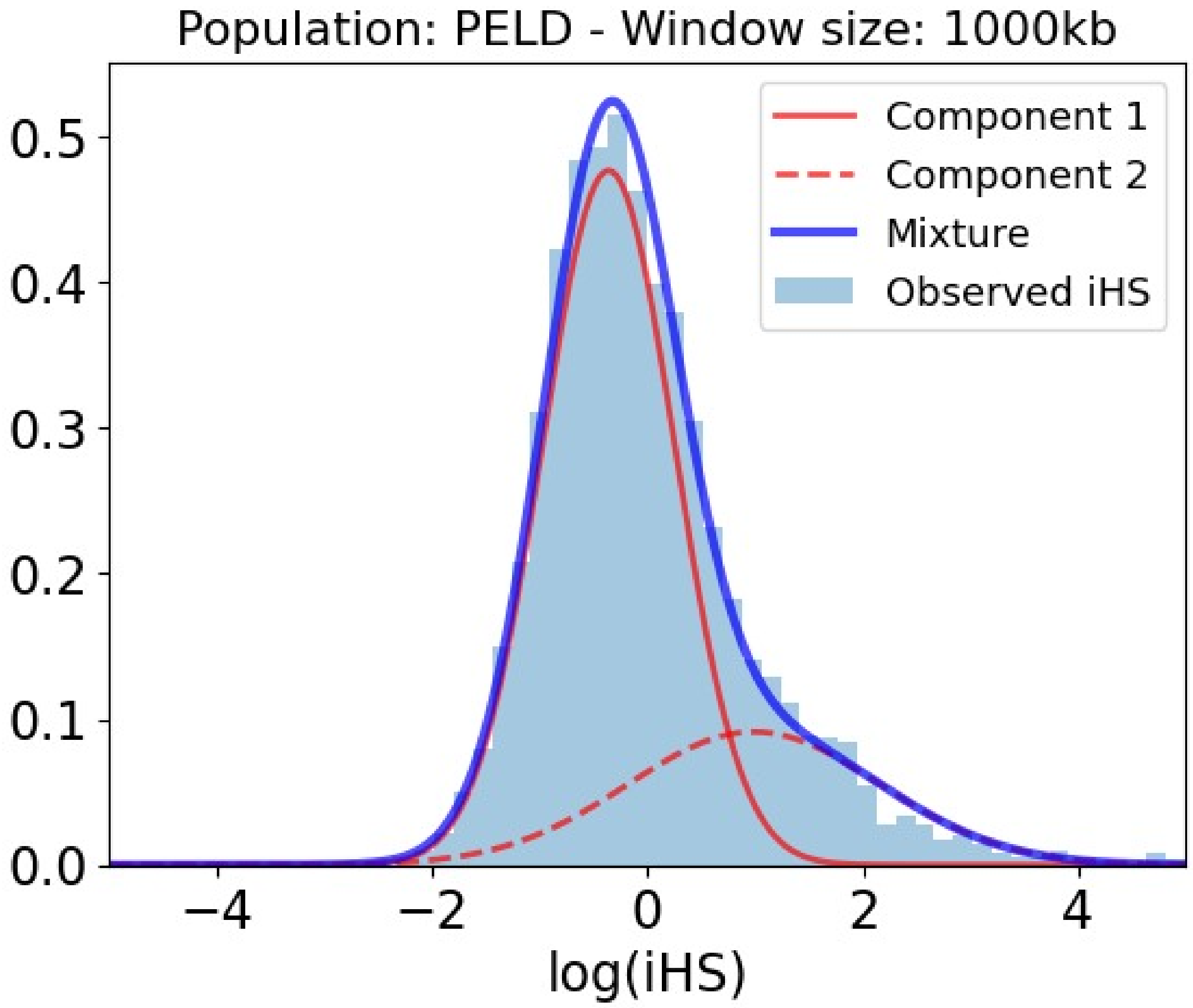

Table S25: Slopes and p-values of the association between iHS and genomic factors for the Peruvians population in 1000kb within the selection-enriched component.

Covariate	Slope	P-value
Intercept	-1.548	0.000E+00
Number iHS data points	0.081	1.506E-03
Density of conserved elements	0.057	2.433E-01
Recombination rate	-1.738	0.000E+00
Number PPIs	-0.037	3.003E-01
Regulatory density (ChIP-seq)	0.327	7.640E-03
Distance to VIPs	-0.178	2.765E-05
Gene number	-0.021	7.960E-01
Coding density	0.202	2.729E-02
Gene length	0.023	5.465E-01
Regulatory density in immune cells (ChIP-seq)	0.146	1.317E-01

**Covariate**	**Slope**	**P-value**
Gene expression	-0.261	2.560E-04
Gene expression in testis	0.053	2.606E-01
Gene expression in immune cells	0.251	1.561E-04
Regulatory density in testis (ChIP-seq)	-0.107	4.757E-02
Regulatory density (DNaseI)	-0.551	8.267E-05
GC-content	0.351	2.628E-03

Supplemental Results S3: Associations between genomic factors and iHS after removing specific predictors

Yoruba

Yoruba 1000kb: Removing recombination rate.

Figure S1: Mixture of Gaussian distributions fitting observed iHS (1000kb windows) for Yoruba. The figure shows the two Gaussian distributions, component 1 and 2 of iHS, being the latter enriched in positive selection. In that component, iHS linearly depends on the genomic factors considered. The figure shows iHS after log transformation and scaling (see Methods). Legend: Light blue = Observed iHS; Dark blue = Mixture model; Full red curve = Component 1 of the mixture model; Dashed red curve = Component 2 of the mixture model enriched in positive selection.

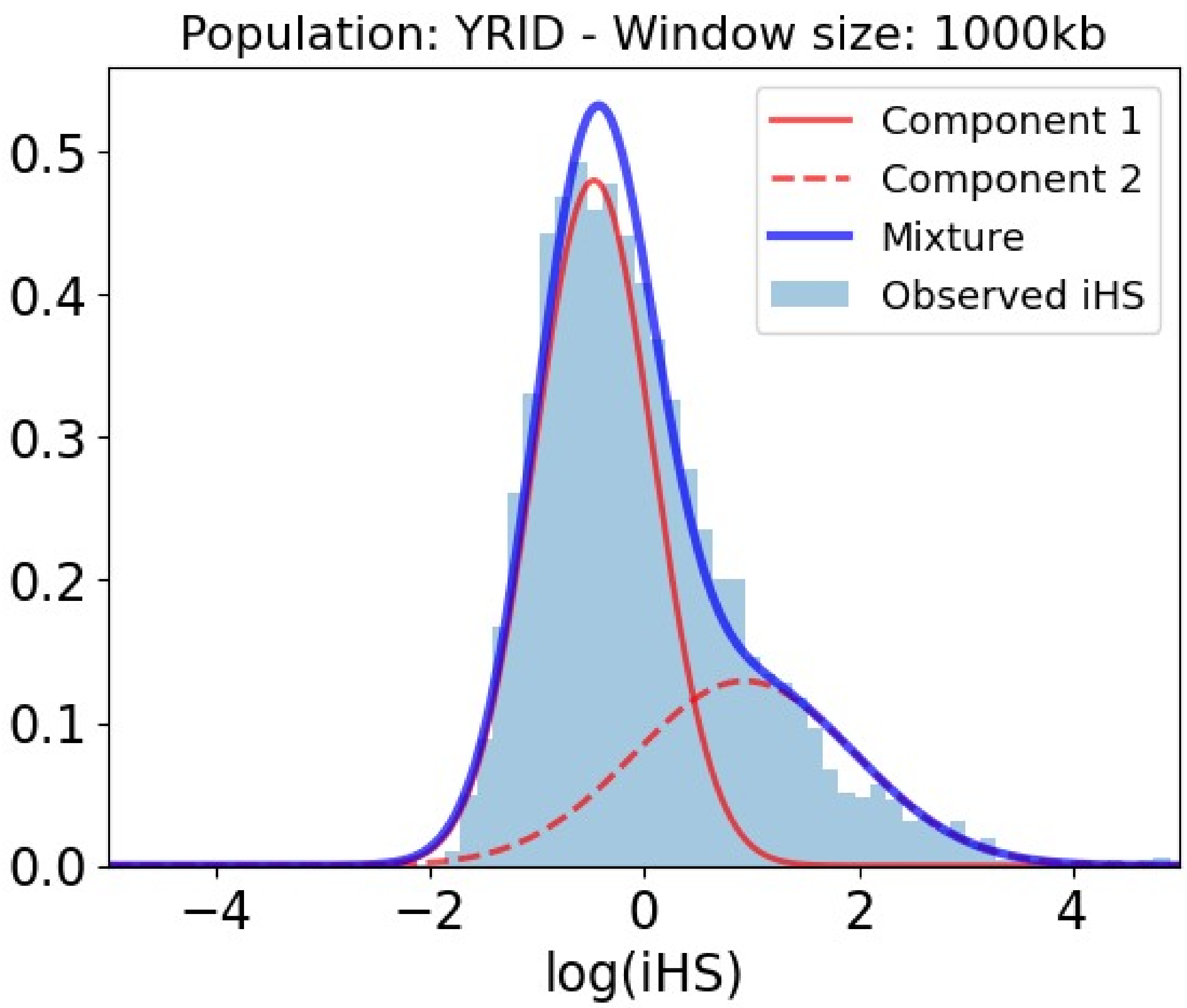

Table S1: Slopes and p-values of the association between iHS and genomic factors for the Yoruba population in 1000kb within the selection-enriched component.

**Covariate**	**Slope**	**P-value**
Intercept	-0.863	0.000E+00
Number iHS data points	-0.872	0.000E+00
Regulatory density (ChIP-seq)	-0.305	6.125E-04
Regulatory density in immune cells (ChIP-seq)	0.271	4.487E-04
Regulatory density in testis (ChIP-seq)	-0.523	0.000E+00
Coding density	0.414	1.209E-07
Density of conserved elements	0.095	1.462E-02
Gene expression	-0.138	1.354E-02
GC-content	-0.287	1.224E-03
Gene length	-0.039	2.278E-01

Covariate	Slope	P-value
Gene number	-0.063	4.293E-01
Gene expression in immune cells	0.260	4.759E-07
Number PPIs	-0.078	6.274E-03
Regulatory density (DNaseI)	-0.127	2.198E-01
Gene expression in testis	0.075	5.629E-02
Distance to VIPs	-0.181	1.460E-07

Yoruba 1000kb: Removing GC-content.

Figure S2: Mixture of Gaussian distributions fitting observed iHS (1000kb windows) for Yoruba. The figure shows the two Gaussian distributions, component 1 and 2 of iHS, being the latter enriched in positive selection. In that component, iHS linearly depends on the genomic factors considered. The figure shows iHS after log transformation and scaling (see Methods). Legend: Light blue = Observed iHS; Dark blue = Mixture model; Full red curve = Component 1 of the mixture model; Dashed red curve = Component 2 of the mixture model enriched in positive selection.

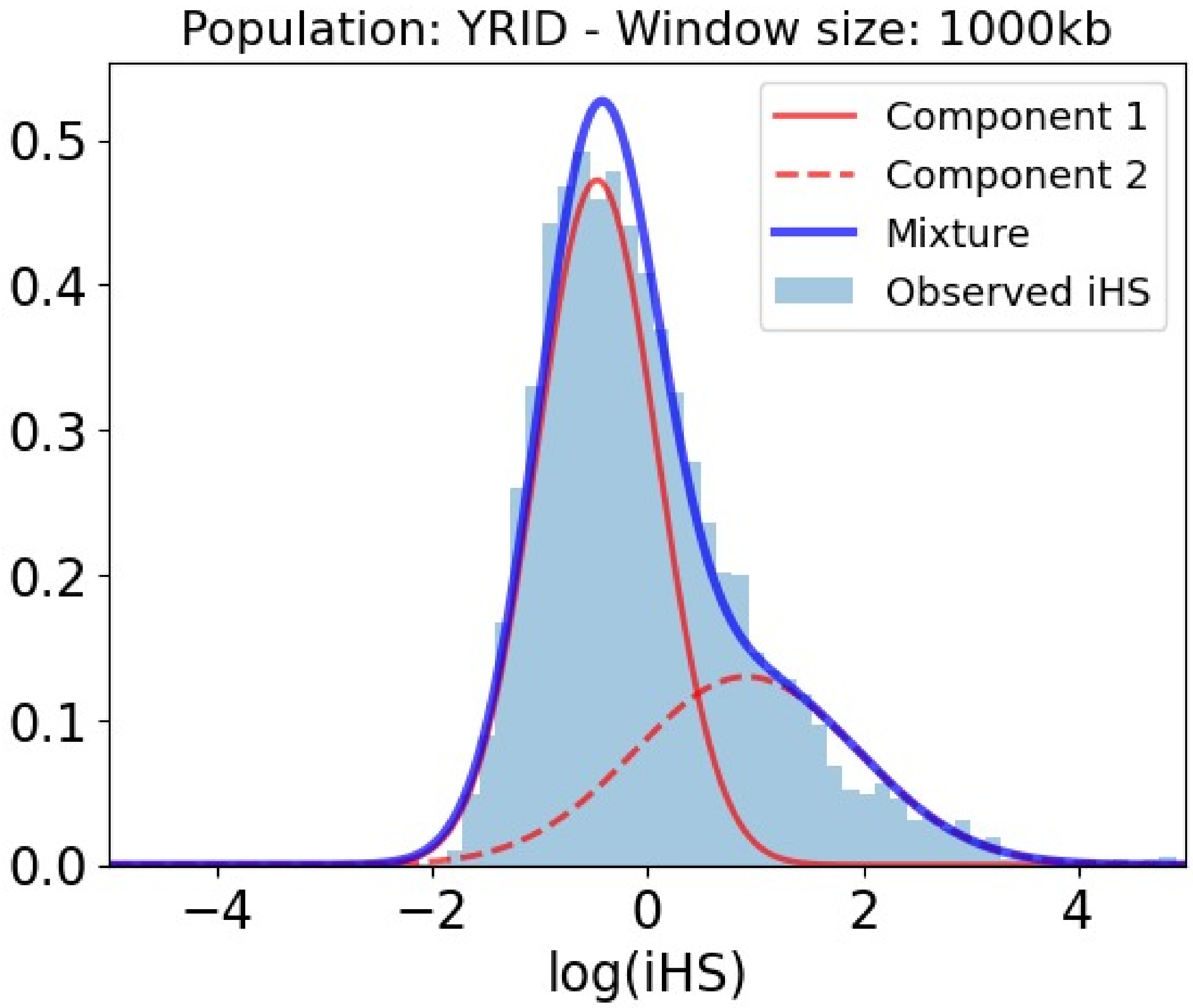

Table S2: Slopes and p-values of the association between iHS and genomic factors for the Yoruba population in 1000kb within the selection-enriched component.

Covariate	Slope	P-value
Intercept	-1.420	0.000E+00
Number iHS data points	-0.027	3.780E-01
Regulatory density (ChIP-seq)	-0.222	7.334E-02
Regulatory density in immune cells (ChIP-seq)	0.072	4.520E-01
Regulatory density in testis (ChIP-seq)	-0.765	0.000E+00
Coding density	0.225	1.321E-02
Density of conserved elements	0.100	2.620E-02
Gene expression	-0.148	2.805E-02
Gene length	-0.061	1.028E-01
Gene number	-0.039	6.654E-01

Covariate	Slope	P-value
Gene expression in immune cells	0.271	9.964E-06
Number PPIs	-0.076	2.607E-02
Recombination rate	-2.390	0.000E+00
Regulatory density (DNaseI)	0.444	1.775E-07
Gene expression in testis	0.027	5.736E-01
Distance to VIPs	-0.177	1.581E-06

Yoruba 1000kb: Removing regulatory density according to DNAseI hypersensitivity and coding density.

Figure S3: Mixture of Gaussian distributions fitting observed iHS (1000kb windows) for Yoruba. The figure shows the two Gaussian distributions, component 1 and 2 of iHS, being the latter enriched in positive selection. In that component, iHS linearly depends on the genomic factors considered. The figure shows iHS after log transformation and scaling (see Methods). Legend: Light blue = Observed iHS; Dark blue = Mixture model; Full red curve = Component 1 of the mixture model; Dashed red curve = Component 2 of the mixture model enriched in positive selection.

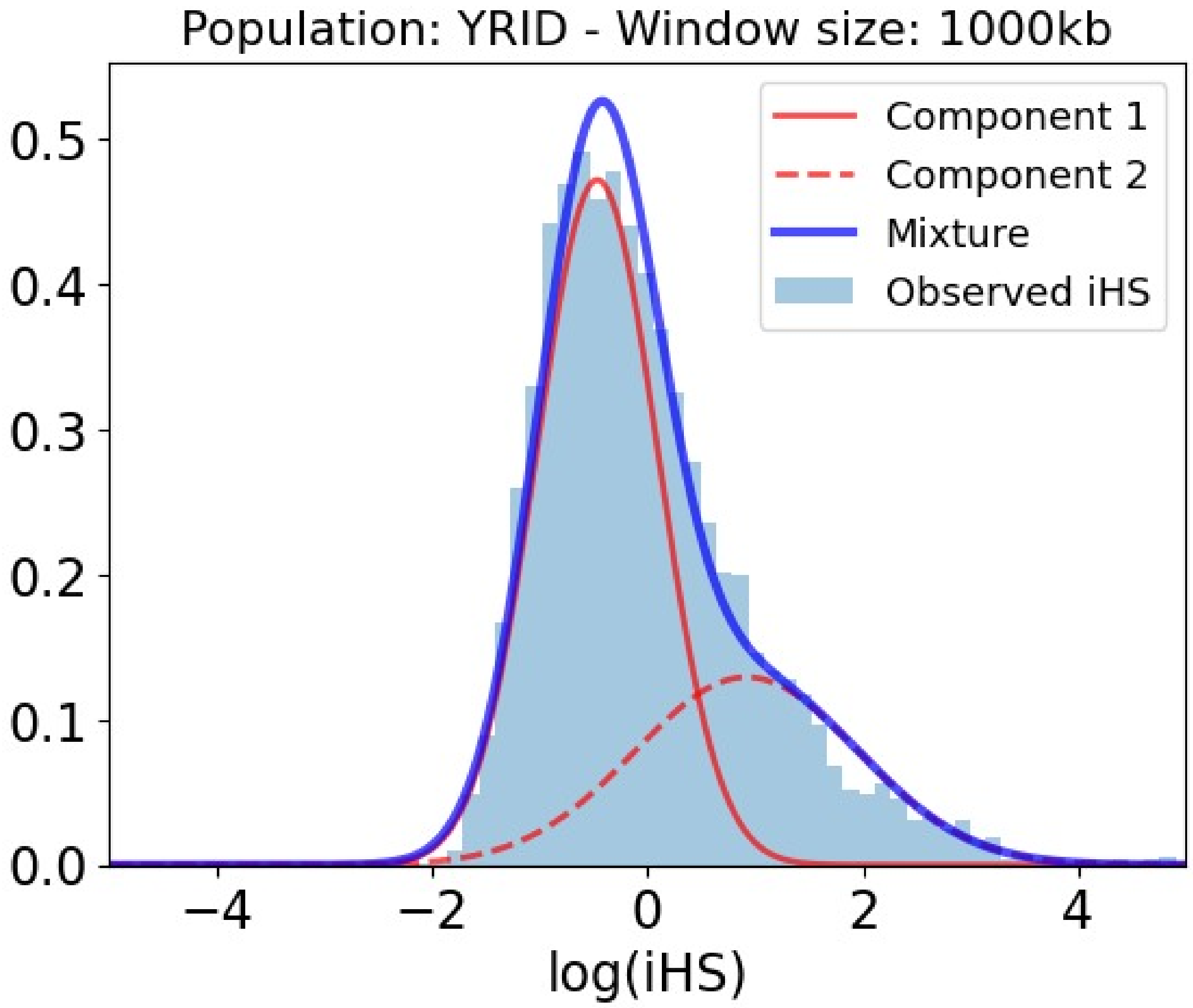

Table S3: Slopes and p-values of the association between iHS and genomic factors for the Yoruba population in 1000kb within the selection-enriched component.

**Covariate**	**Slope**	**P-value**
Intercept	-1.433	0.000E+00
Number iHS data points	-0.003	9.285E-01
Regulatory density (ChIP-seq)	-0.175	1.029E-01
Regulatory density in immune cells (ChIP-seq)	0.037	6.950E-01
Regulatory density in testis (ChIP-seq)	-0.783	0.000E+00
Density of conserved elements	0.183	2.482E-05
Gene expression	-0.134	4.510E-02
GC-content	0.543	2.398E-14
Gene length	-0.058	1.195E-01
Gene number	0.042	4.417E-01

Covariate	Slope	P-value
Gene expression in immune cells	0.264	1.699E-05
Number PPIs	-0.073	3.572E-02
Recombination rate	-2.451	0.000E+00
Gene expression in testis	0.006	9.036E-01
Distance to VIPs	-0.169	4.627E-06

Yoruba 1000kb: Removing regulatory density according to DNAseI sensitivity, coding density, regulatory density according to ChIP-seq experiments across multiple tissues and specifically in immune cells, gene number and gene length.

Figure S4: Mixture of Gaussian distributions fitting observed iHS (1000kb windows) for Yoruba. The figure shows the two Gaussian distributions, component 1 and 2 of iHS, being the latter enriched in positive selection. In that component, iHS linearly depends on the genomic factors considered. The figure shows iHS after log transformation and scaling (see Methods). Legend: Light blue = Observed iHS; Dark blue = Mixture model; Full red curve = Component 1 of the mixture model; Dashed red curve = Component 2 of the mixture model enriched in positive selection.

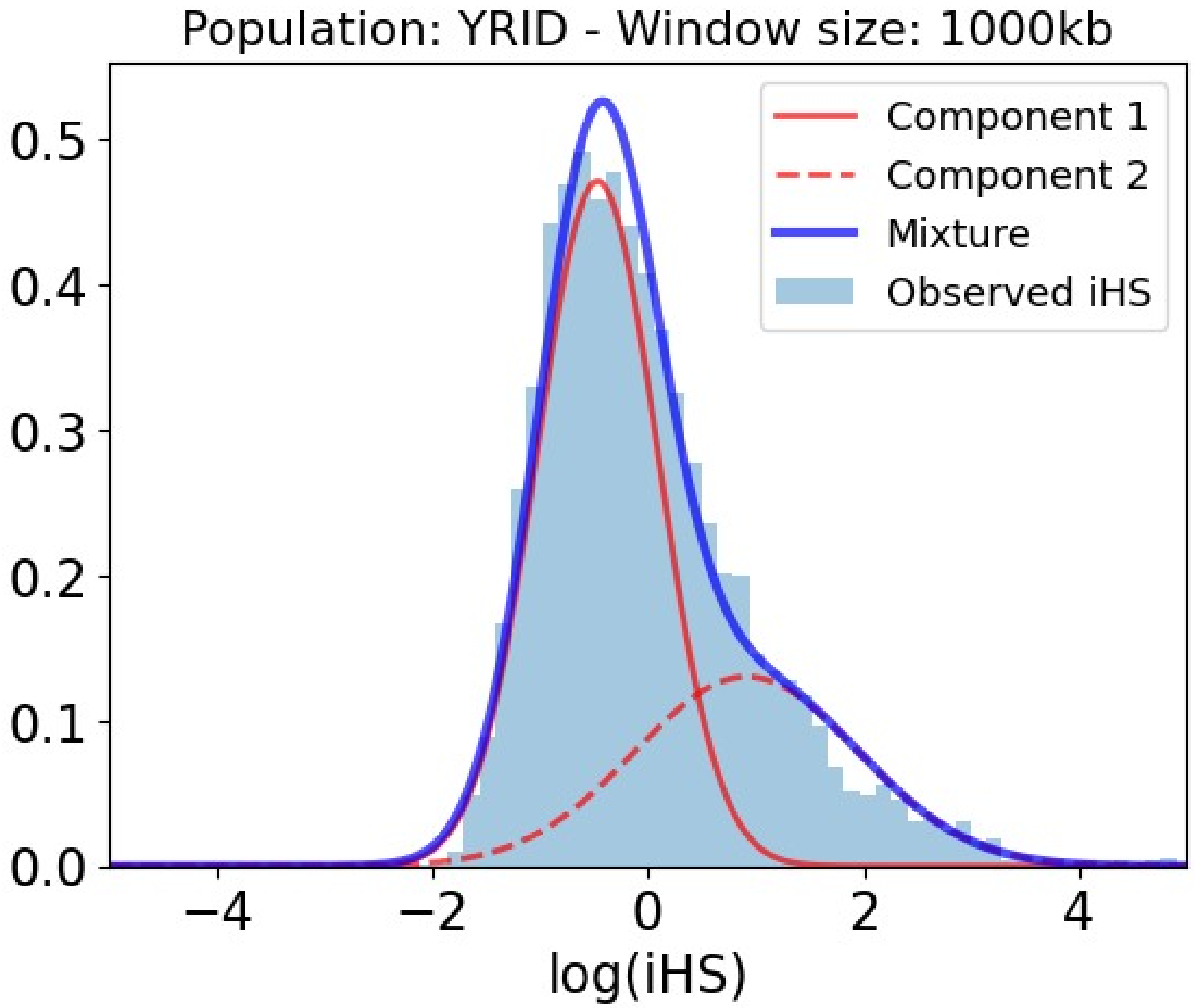

Table S4: Slopes and p-values of the association between iHS and genomic factors for the Yoruba population in 1000kb within the selection-enriched component.

**Covariate**	**Slope**	**P-value**
Intercept	-1.412	0.000E+00
Number iHS data points	-0.003	9.021E-01
Regulatory density in testis (ChIP-seq)	-0.792	0.000E+00
Density of conserved elements	0.170	2.757E-05
Gene expression	-0.154	1.781E-02
GC-content	0.492	0.000E+00
Gene expression in immune cells	0.274	3.358E-06
Number PPIs	-0.080	1.953E-02
Recombination rate	-2.466	0.000E+00
Gene expression in testis	0.002	9.685E-01

Covariate	Slope	P-value
Distance to VIPs	-0.176	7.015E-07

Yoruba 1000kb: Removing the density of conserved elements.

Figure S5: Mixture of Gaussian distributions fitting observed iHS (1000kb windows) for Yoruba. The figure shows the two Gaussian distributions, component 1 and 2 of iHS, being the latter enriched in positive selection. In that component, iHS linearly depends on the genomic factors considered. The figure shows iHS after log transformation and scaling (see Methods). Legend: Light blue = Observed iHS; Dark blue = Mixture model; Full red curve = Component 1 of the mixture model; Dashed red curve = Component 2 of the mixture model enriched in positive selection.

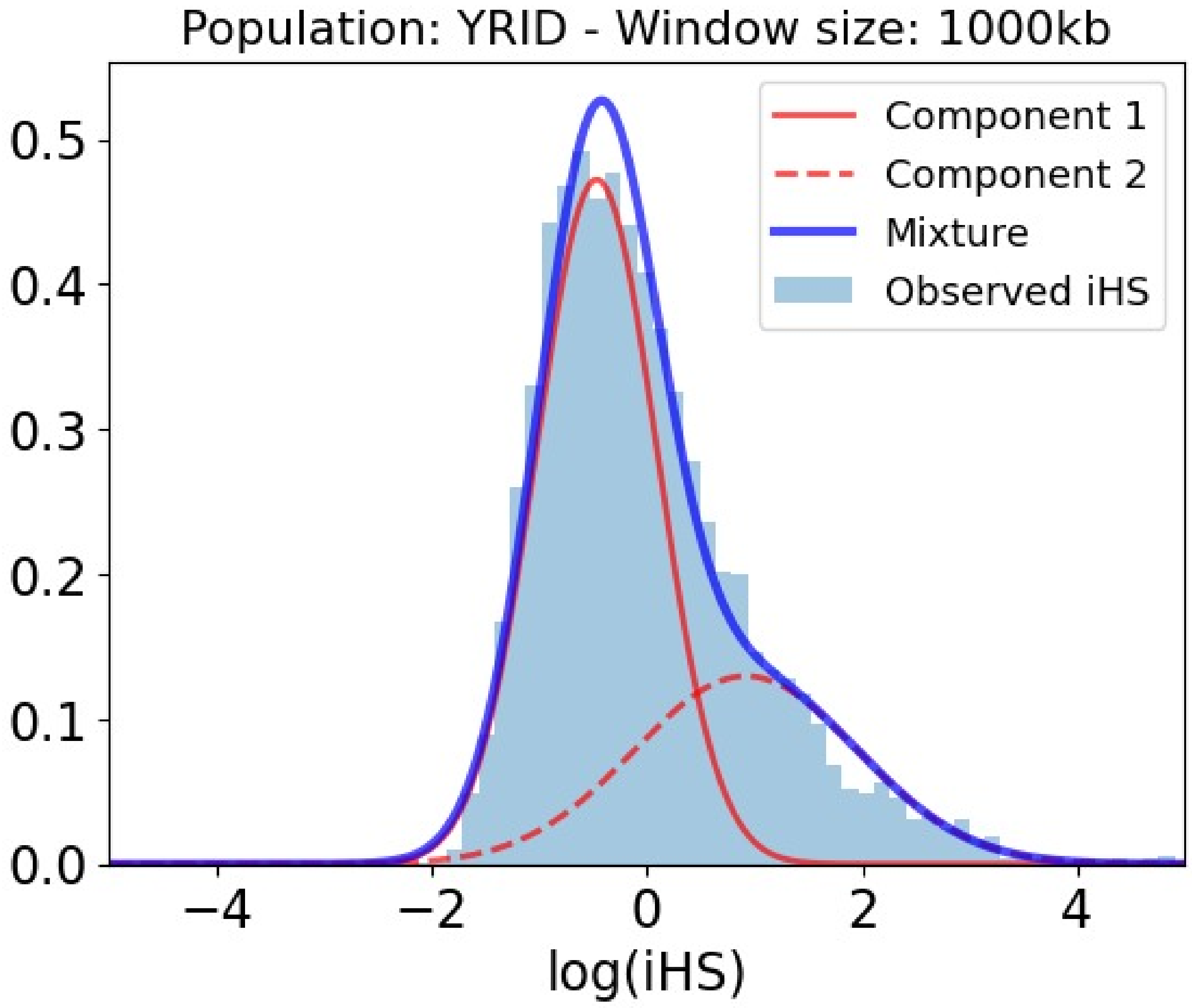

Table S5: Slopes and p-values of the association between iHS and genomic factors for the

Yoruba population in 1000kb within the selection-enriched component.

Covariate	Slope	P-value
Intercept	-1.411	0.000E+00
Number iHS data points	-0.033	2.703E-01
Regulatory density (ChIP-seq)	-0.135	2.741E-01
Regulatory density in immune cells (ChIP-seq)	0.095	3.160E-01
Regulatory density in testis (ChIP-seq)	-0.763	0.000E+00
Coding density	0.259	2.347E-03
Gene expression	-0.126	5.905E-02
GC-content	0.447	4.494E-05
Gene length	-0.046	2.151E-01
Gene number	-0.120	1.822E-01
Gene expression in immune cells	0.249	4.291E-05
Number PPIs	-0.068	4.944E-02
Recombination rate	-2.425	0.000E+00
Regulatory density (DNaseI)	0.023	8.815E-01
Gene expression in testis	0.010	8.351E-01
Distance to VIPs	-0.174	2.795E-06

Yoruba 1000kb: Removing the density of conserved elements and GC-content.

Figure S6: Mixture of Gaussian distributions fitting observed iHS (1000kb windows) for Yoruba. The figure shows the two Gaussian distributions, component 1 and 2 of iHS, being the latter enriched in positive selection. In that component, iHS linearly depends on the genomic factors considered. The figure shows iHS after log transformation and scaling (see Methods). Legend: Light blue = Observed iHS; Dark blue = Mixture model; Full red curve = Component 1 of the mixture model; Dashed red curve = Component 2 of the mixture model enriched in positive selection.

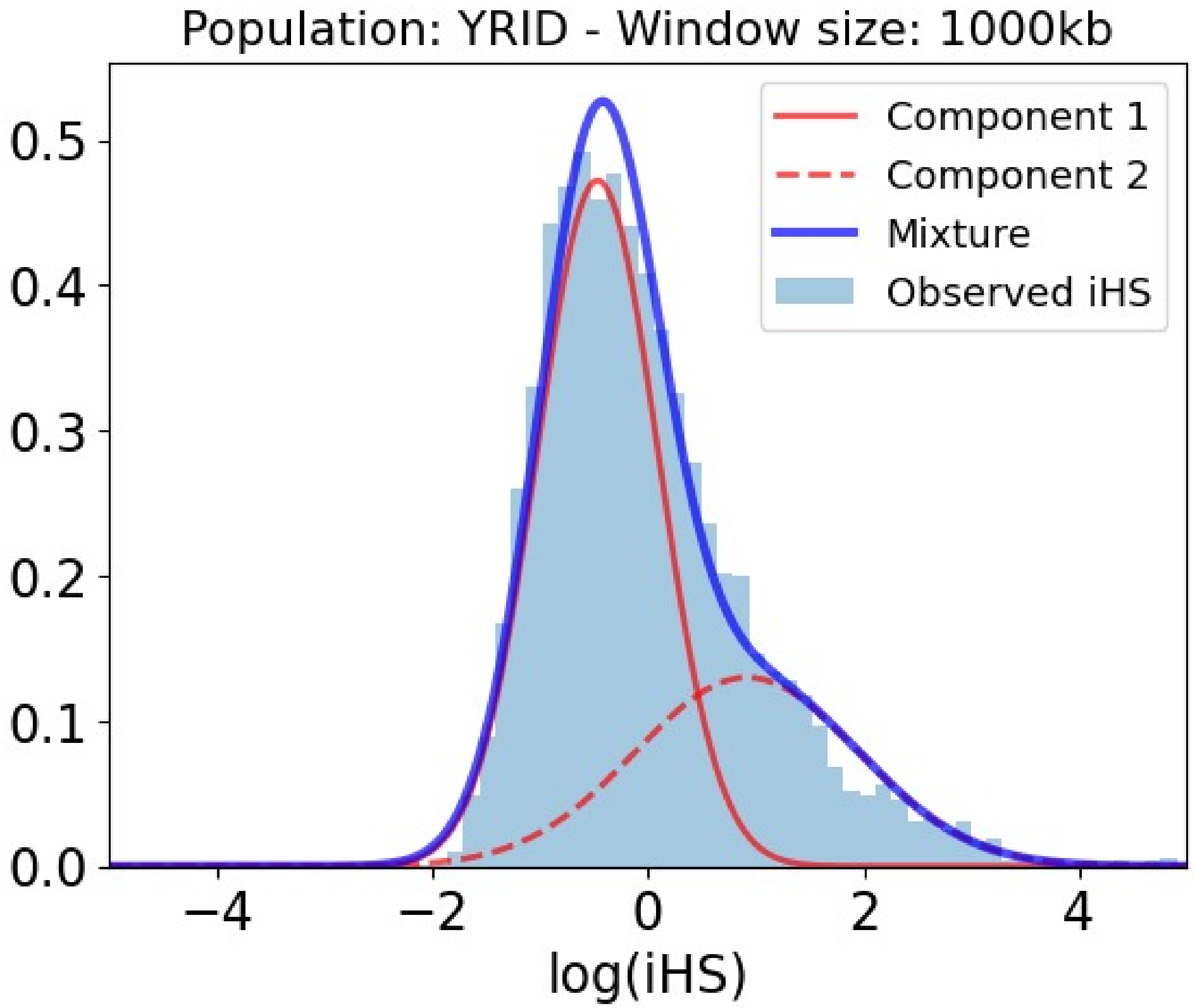

Table S6: Slopes and p-values of the association between iHS and genomic factors for the Yoruba population in 1000kb within the selection-enriched component.

**Covariate**	**Slope**	**P-value**
Intercept	-1.409	0.000E+00
Number iHS data points	-0.041	1.612E-01
Regulatory density (ChIP-seq)	-0.202	9.854E-02
Regulatory density in immune cells (ChIP-seq)	0.099	2.894E-01
Regulatory density in testis (ChIP-seq)	-0.749	0.000E+00
Coding density	0.287	7.763E-04
Gene expression	-0.138	3.976E-02
Gene length	-0.052	1.618E-01
Gene number	-0.070	4.440E-01
Gene expression in immune	0.260	2.090E-05

Covariate	Slope	P-value
cells		
Number PPIs	-0.074	3.108E-02
Recombination rate	-2.387	0.000E+00
Regulatory density (DNaseI)	0.427	4.012E-07
Gene expression in testis	0.029	5.363E-01
Distance to VIPs	-0.180	1.293E-06

Yoruba 1000kb: Removing no predictor.

Figure S7: Mixture of Gaussian distributions fitting observed iHS (1000kb windows) for Yoruba. The figure shows the two Gaussian distributions, component 1 and 2 of iHS, being the latter enriched in positive selection. In that component, iHS linearly depends on the genomic factors considered. The figure shows iHS after log transformation and scaling (see Methods). Legend: Light blue = Observed iHS; Dark blue = Mixture model; Full red curve = Component 1 of the mixture model; Dashed red curve = Component 2 of the mixture model enriched in positive selection.

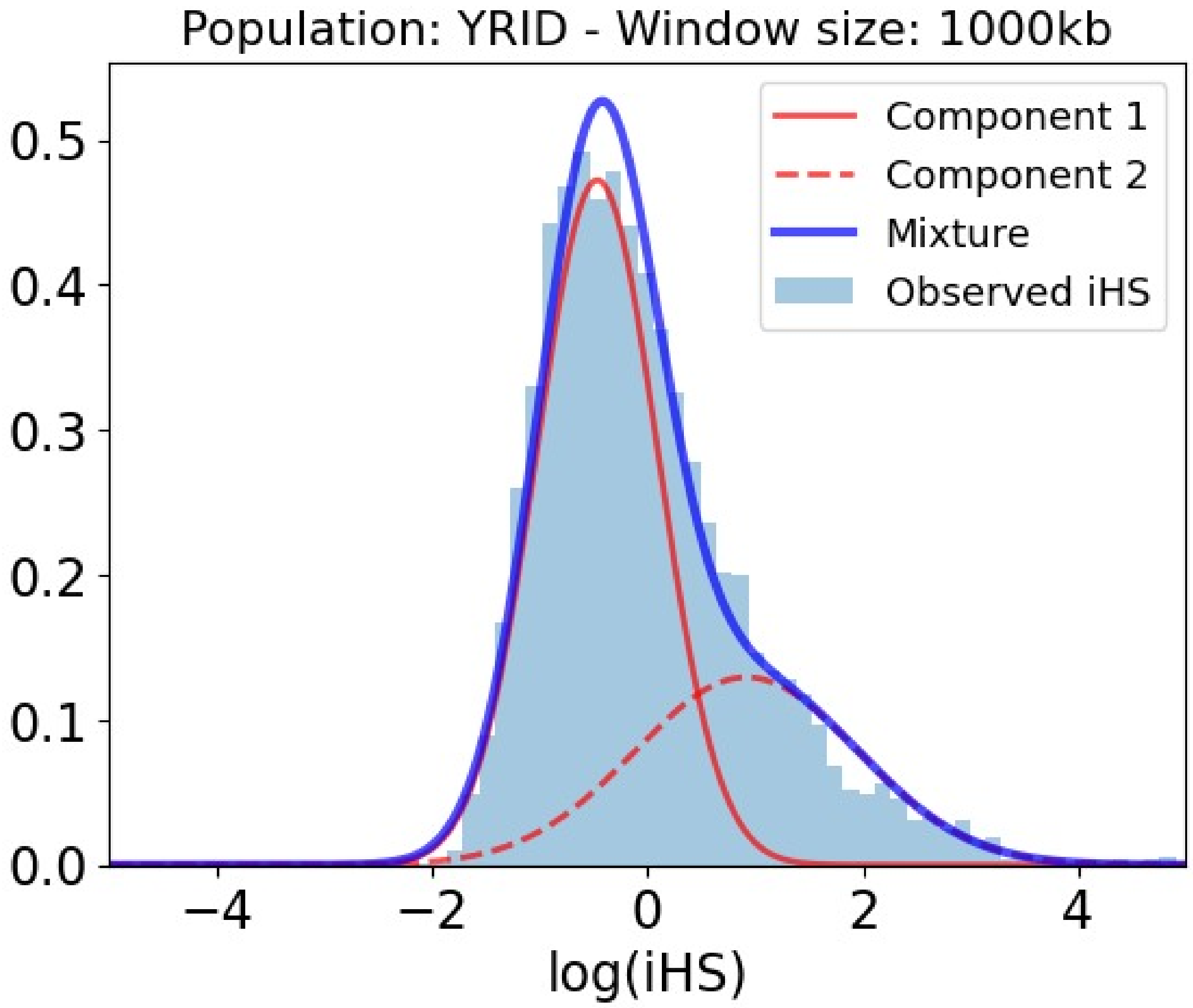

Table S7: Slopes and p-values of the association between iHS and genomic factors for the Yoruba population in 1000kb within the selection-enriched component.

**Covariate**	**Slope**	**P-value**
Intercept	-1.426	0.000E+00
Number iHS data points	-0.007	8.190E-01
Regulatory density (ChIP-seq)	-0.147	2.372E-01
Regulatory density in immune cells (ChIP-seq)	0.047	6.230E-01
Regulatory density in testis (ChIP-seq)	-0.795	0.000E+00
Coding density	0.159	8.411E-02
Density of conserved elements	0.159	6.397E-04
Gene expression	-0.139	3.768E-02
GC-content	0.550	1.339E-06
Gene length	-0.059	1.124E-01

Covariate	Slope	P-value
Gene number	-0.086	3.458E-01
Gene expression in immune cells	0.265	1.512E-05
Number PPIs	-0.070	4.252E-02
Recombination rate	-2.436	0.000E+00
Regulatory density (DNaseI)	-0.047	7.138E-01
Gene expression in testis	0.001	9.803E-01
Distance to VIPs	-0.170	4.160E-06

Supplemental Results S4: Fine scale analyses of recombination rate, regulatory density and positive selection

iHS, recombination rate and distance to regulatory elements

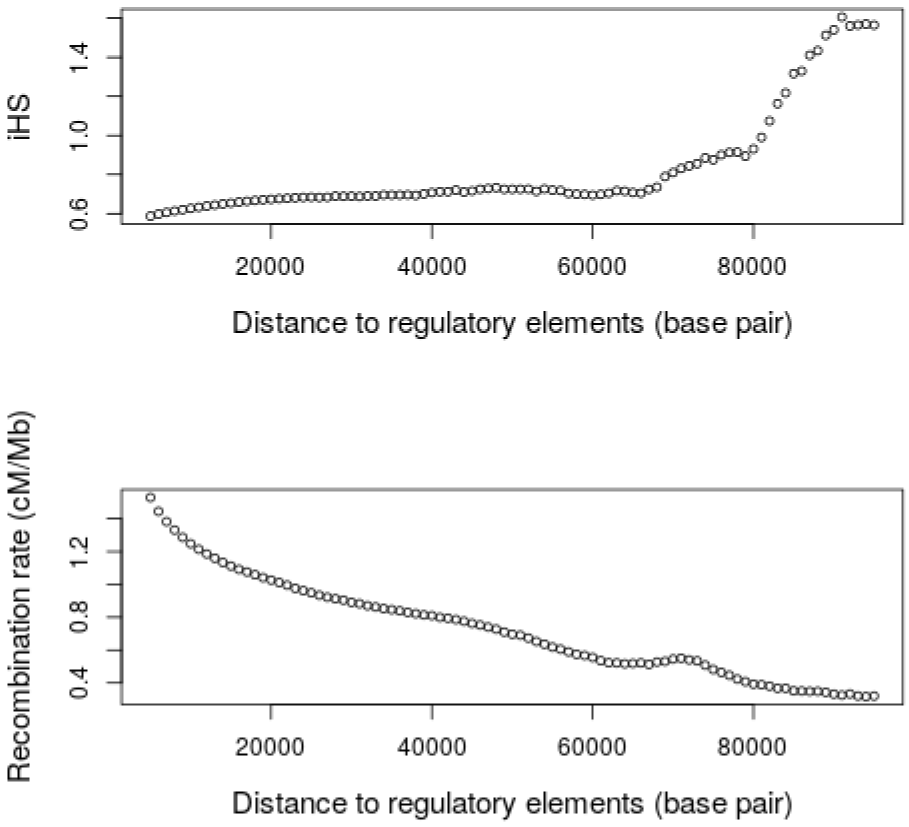

Figure S1: Association between distance to regulatory elements (ChIP-seq), iHS and recombination rate at a fine scale. For each of the studied SNPs in Yoruba, we calculated the distance to the center of the closest regulatory element. We also calculated the recombination rate in 100 kb windows centered around each SNP included in this study. We used the same approach than for the calculation of recombination rate in gene-centered windows (see Methods). We considered SNPs separated up to 100 kb from the closest regulatory element to focus on the fine scale pat-

terns of selection and recombination around regulatory elements. We calculated a moving average using sliding windows of 10 kb with a separation of 1 kb. For example, we calculated the average iHS and recombination rate of all SNPs at distance to regulatory elements between 0 and 10 kb. In the next averages, we considered SNPs between 1 and 11 kb, 2 and 12 kb and so on. The resulting averages are shown for iHS in the upper panel and recombination in the lower panel.

iHS, recombination rate and distance to regulatory elements in testis

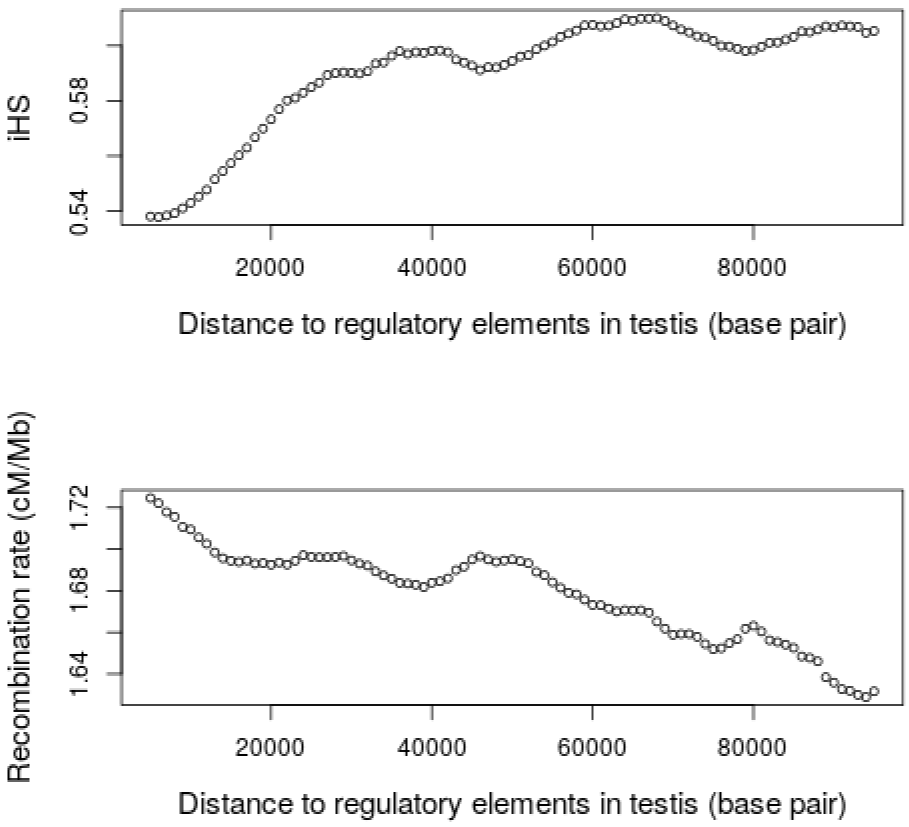

Figure S2: Association between distance to regulatory elements in testis (ChIP-seq), iHS and recombination rate at a fine scale. We used the same approach explained in Figure S1 but calculating the distance of each SNP to the center of the closest regulatory element in testis. The upper

panel shows the association between the distance to regulatory elements in testis and iHS, while the lower panel show the association with recombination rate.

iHS, recombination rate and distance to regulatory elements in lymphocytes

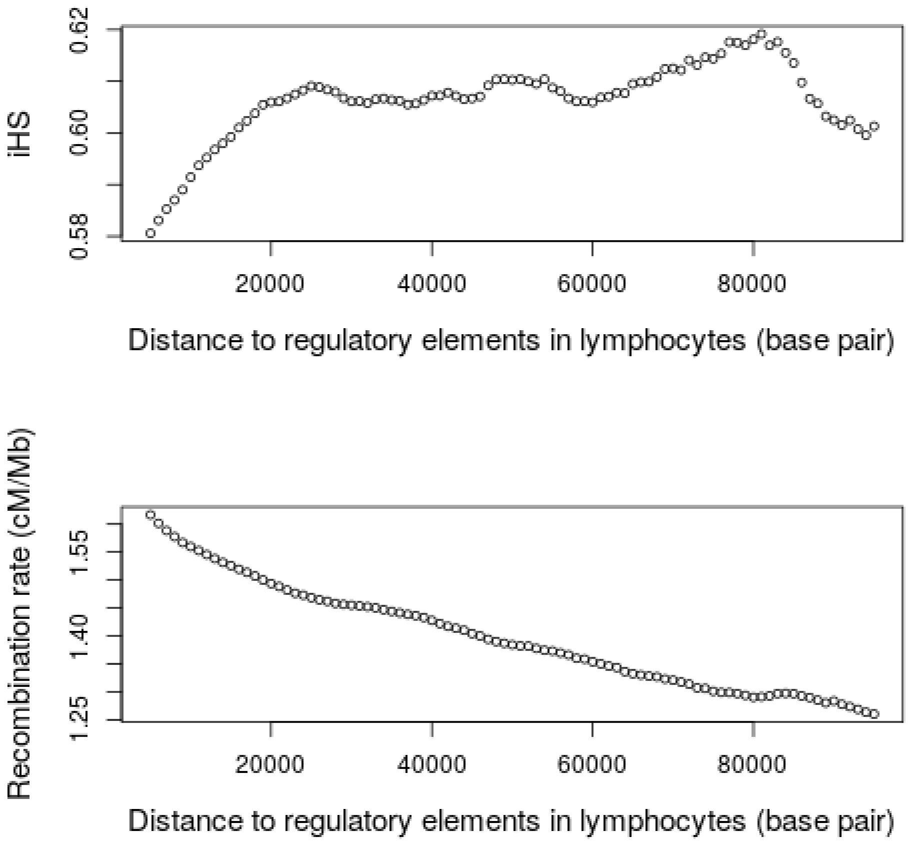

Figure S3: Association between distance to regulatory elements in lymphocytes (ChIP-seq), iHS and recombination rate at a fine scale. We used the same approach explained in Figure S1 but calculating the distance of each SNP to the center of the closest regulatory element in lymphocytes. The upper panel shows the association between the distance to regulatory elements in lymphocytes and iHS, while the lower panel show the association with recombination rate.

Recombination rate and the three regulatory densities

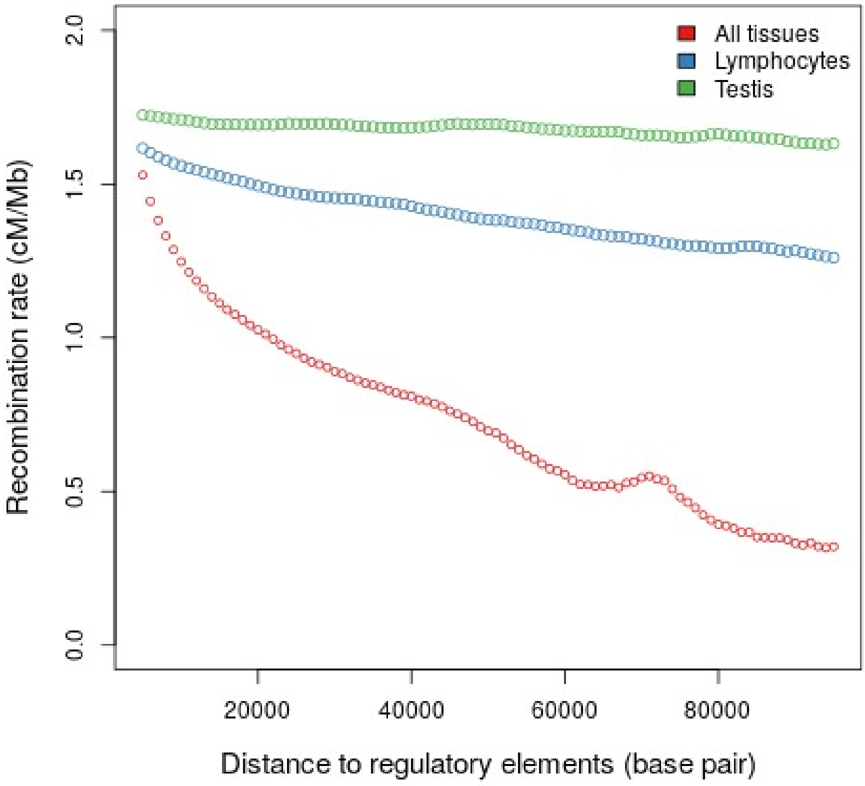

Figure S4: Association between recombination rate and the distance to regulatory elements in lymphocytes, testis and all tissues (ChIP-seq) at a fine scale. The plot shows the same average recombination rate previously calculated as a function of the distance to regulatory elements, but showing the three types of regulatory datasets at the same time.

iHS, recombination rate and distance to coding + regulatory elements

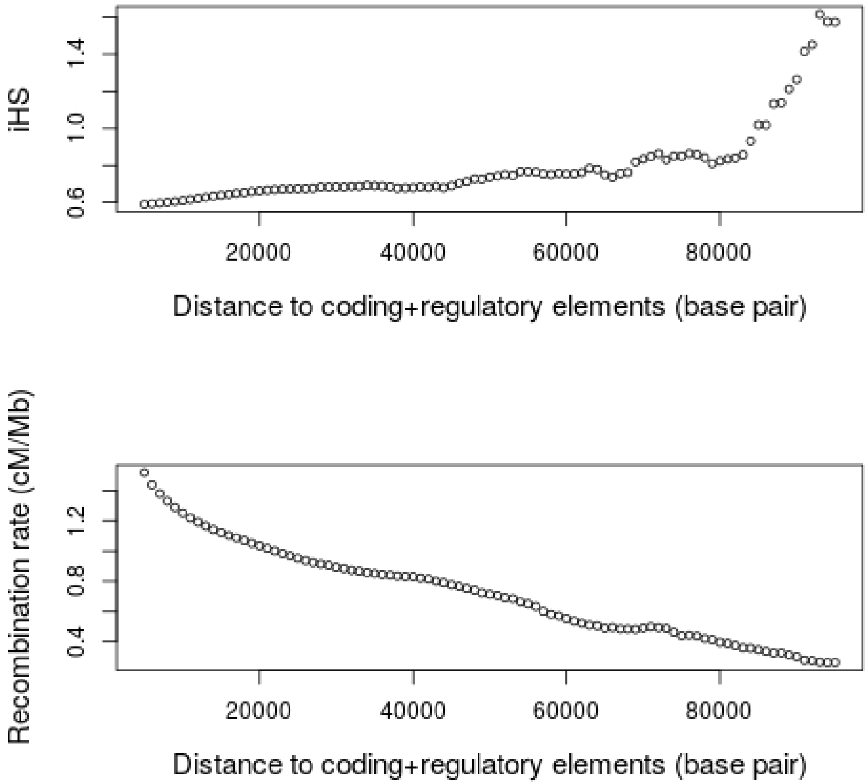

Figure S5: Association between distance to coding and regulatory elements (ChIP-seq), iHS and recombination rate at a fine scale. We used the same approach explained in Figure S1 but also considering the distance to center of coding elements. In other words, we calculated the distance between each SNP and the closest coding or regulatory element (whatever is closer). The upper panel shows the association between the distance to coding/regulatory elements and iHS, while the lower panel show the association with recombination rate.

Modeling results from fine scale-recombination analyses

Next sections include the results for analyses using variables about recombination around regulatory elements. We considered three sets of regulatory elements, that is, transcription factor binding sites across all tissues included in ChIP-seq experiments (UCSC Genome Browser), along with binding sites in lymphocytes and testis (see Methods for further details). In order to calculate the recombination around regulatory elements, we applied the following approach: For each set of regulatory elements, we created a 10 kb genomic window around the center of each element (5 kb at each side; regulatory window hereafter). We then fused those regulatory windows that were overlapped in at least 1 base. Next, recombination rate was calculated for the resulting regulatory windows. We followed the same approach than for gene windows, that is, calculating the ratio of genetic to physical distance between the ends of each regulatory window. In order to calculate the genetic position of each edge of the regulatory window, we again applied the same approach than in the case of gene windows. We searched for data about genetic position around each edge of the window up to 10 kb at each side, using then linear interpolation to estimate the genetic position of the corresponding edge (see Methods section for further details). Once the recombination rate around each regulatory window was estimated, we calculated the average of recombination of all regulatory windows completely overlapped with each gene window. In other words, we obtained a value of average recombination around regulatory elements for each gene window. We also counted the number of recombination data points, i.e., the number of regulatory windows for which recombination could be calculated in each gene window. In that way, we attempted to control for the potential noise caused by variability in the number of regulatory windows between gene windows.

We applied this approach separately for each regulatory variable, that is, considering the regulatory elements across all tissues, but also considering only regulatory elements in lymphocytes and testis in parallel calculations. Therefore, we obtained three different variables related to the recombination: recombination around regulatory elements in lymphocytes, testis and across all tissues. Each variable was included in the original model separately, being present only one recombination variable each time. We also run models that included one of these variables and the original recombination variable, that is, recombination rate across gene windows (window-

wide scale hereafter). In that way, we attempted to control for the possibility that these new variables would not fully account for the influence of recombination at a wider scale. Similar results after including the original window-wide recombination variable would suggest that our results are not caused by a lack of control of recombination at a window-wide scale. Finally, we repeated the analyses considering only high recombination regions, that is, analyzing only genes with a recombination rate (at a window-wide level) equal or higher than the second tertile (1.552 cM/Mb). We further limited in this way the confounding effect caused by low-recombination regions, i.e., a mismatch between recombination at different scales: low at the window-wide level, but high at local level around regulatory elements. This mismatch should not exist in high recombination regions, where recombination is also high at the window-wide level. Only the Yoruba population and 1,000 kb windows were analyzed.

Yoruba

Yoruba 1000kb: Recombination around lymphocytes regulatory elements without original recombination

Figure S6: Mixture of Gaussian distributions fitting observed iHS (1000kb windows) for Yoruba. The figure shows the two Gaussian distributions, component 1 and 2 of iHS, being the latter enriched in positive selection. In that component, iHS linearly depends on the genomic factors considered. The figure shows iHS after log transformation and scaling (see Methods). Legend: Light blue = Observed iHS; Dark blue = Mixture model; Full red curve = Component 1 of the mixture model; Dashed red curve = Component 2 of the mixture model enriched in positive selection.

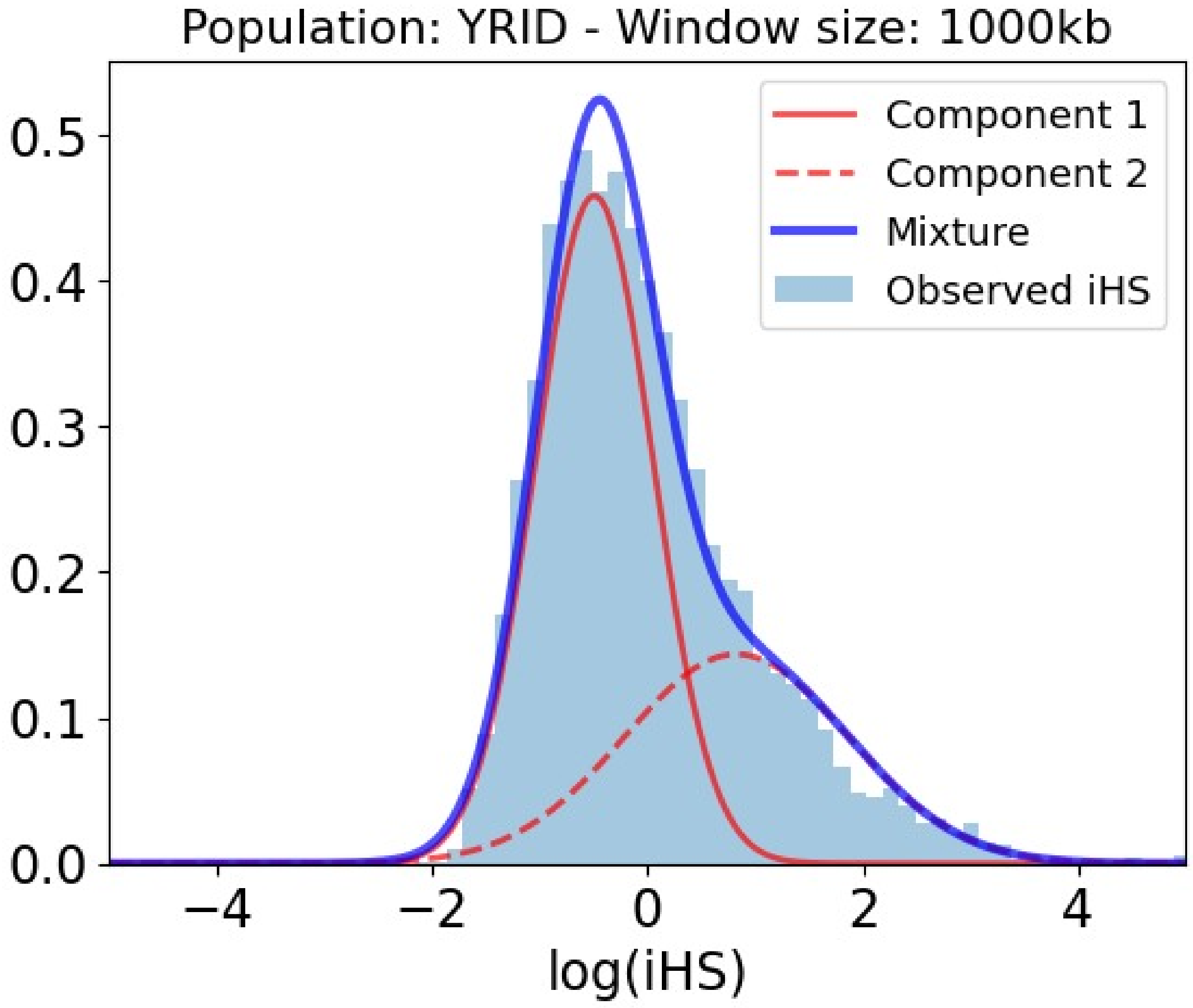

Table S1: Slopes and p-values of the association between iHS and genomic factors for the Yoruba population in 1000kb within the selection-enriched component.

Covariate	Slope	P-value
Intercept	-0.724	0.000E+00

Covariate	Slope	P-value
Number iHS data points	-0.193	1.629E-10
Regulatory density (ChIP-seq)	-0.261	1.029E-02
Regulatory density in immune cells (ChIP-seq)	0.680	4.596E-14
Recombination rate around regulatory elements in lymphocytes (ChIP-seq)	-0.451	0.000E+00
Number of recombination rate data points around regulatory elements in lymphocytes (ChIP-seq)	-1.289	0.000E+00
Regulatory density in testis (ChIP-seq)	-0.808	0.000E+00
Coding density	0.349	5.309E-04
Density of conserved elements	0.078	9.643E-02
Gene expression	-0.114	8.713E-02
GC-content	-0.118	2.687E-01
Gene length	-0.100	7.104E-03
Gene number	0.056	5.203E-01
Gene expression in immune cells	0.273	6.407E-06
Number PPIs	-0.108	1.736E-03
Regulatory density (DNaseI)	0.336	5.579E-03
Gene expression in testis	0.062	1.808E-01
Distance to VIPs	-0.313	0.000E+00

Yoruba 1000kb: Recombination around lymphocytes regulatory elements and original recombination

Figure S7: Mixture of Gaussian distributions fitting observed iHS (1000kb windows) for Yoruba. The figure shows the two Gaussian distributions, component 1 and 2 of iHS, being the latter enriched in positive selection. In that component, iHS linearly depends on the genomic factors considered. The figure shows iHS after log transformation and scaling (see Methods). Legend: Light blue = Observed iHS; Dark blue = Mixture model; Full red curve = Component 1 of the mixture model; Dashed red curve = Component 2 of the mixture model enriched in positive selection.

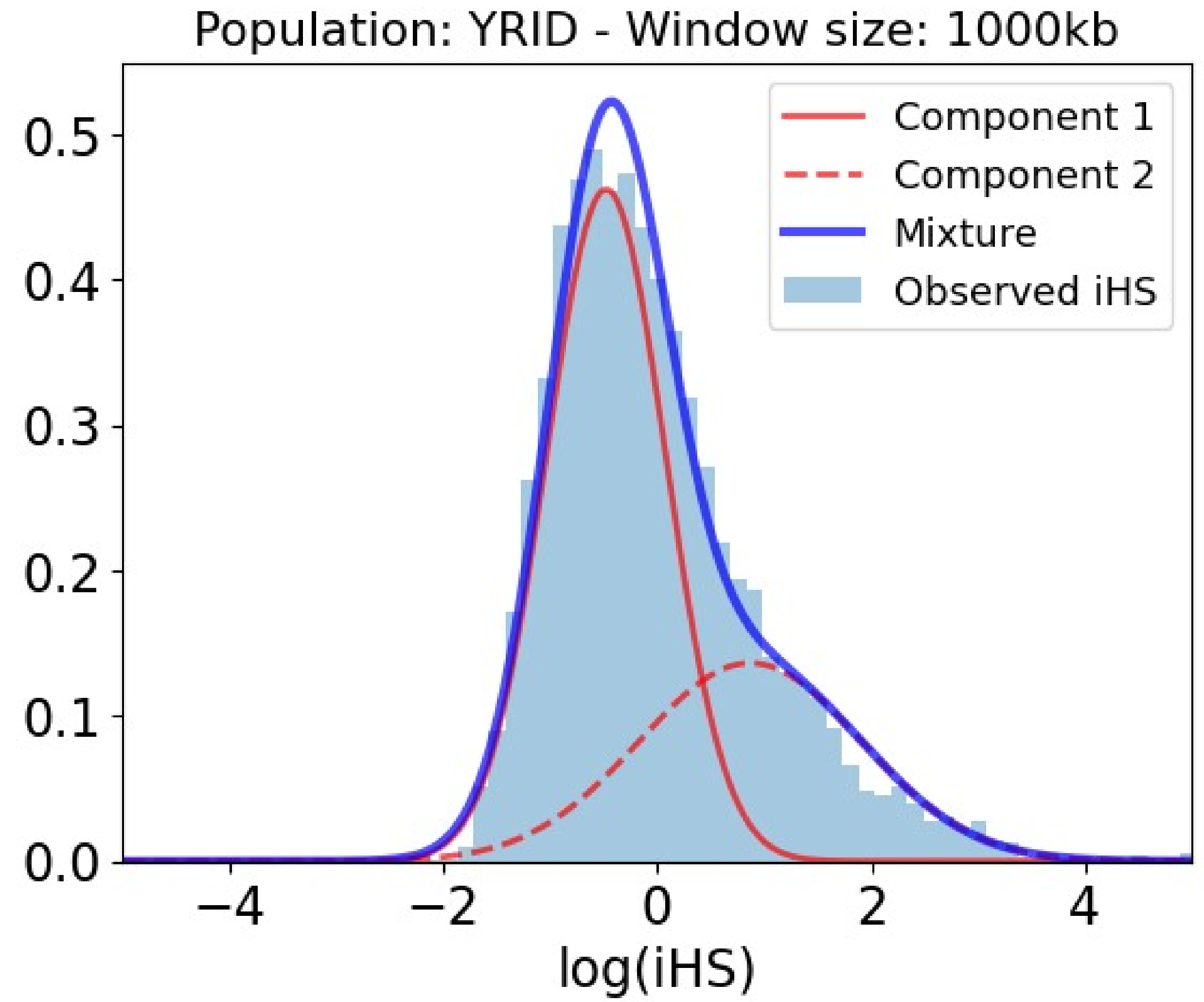

Table S2: Slopes and p-values of the association between iHS and genomic factors for the Yoruba population in 1000kb within the selection-enriched component.

**Covariate**	**Slope**	**P-value**
Intercept	-1.179	0.000E+00
Number iHS data points	0.045	1.529E-01
Regulatory density (ChIP-seq)	-0.073	5.309E-01
Regulatory density in immune cells (ChIP-seq)	0.275	5.717E-03
Recombination rate around regulatory elements in lymphocytes (ChIP-seq)	0.346	9.220E-10
Number of recombination rate data points around regulatory elements in lymphocytes (ChIP-seq)	-0.757	0.000E+00
Regulatory density in testis (ChIP-seq)	-0.909	0.000E+00
Coding density	0.144	1.789E-01

Covariate	Slope	P-value
Density of conserved elements	0.106	3.226E-02
Gene expression	-0.086	2.223E-01
GC-content	0.390	1.036E-03
Gene length	-0.085	2.672E-02
Gene number	0.020	8.623E-01
Gene expression in immune cells	0.219	6.323E-04
Number PPIs	-0.088	1.773E-02
Recombination rate	-2.110	0.000E+00
Regulatory density (DNaseI)	0.252	6.920E-02
Gene expression in testis	0.021	6.771E-01
Distance to VIPs	-0.259	1.180E-10

Yoruba 1000kb: Recombination around lymphocytes regulatory elements and original recombination in high recombination regions

Figure S8: Mixture of Gaussian distributions fitting observed iHS (1000kb windows) for Yoruba. The figure shows the two Gaussian distributions, component 1 and 2 of iHS, being the latter enriched in positive selection. In that component, iHS linearly depends on the genomic factors considered. The figure shows iHS after log transformation and scaling (see Methods). Legend: Light blue = Observed iHS; Dark blue = Mixture model; Full red curve = Component 1 of the mixture model; Dashed red curve = Component 2 of the mixture model enriched in positive selection.

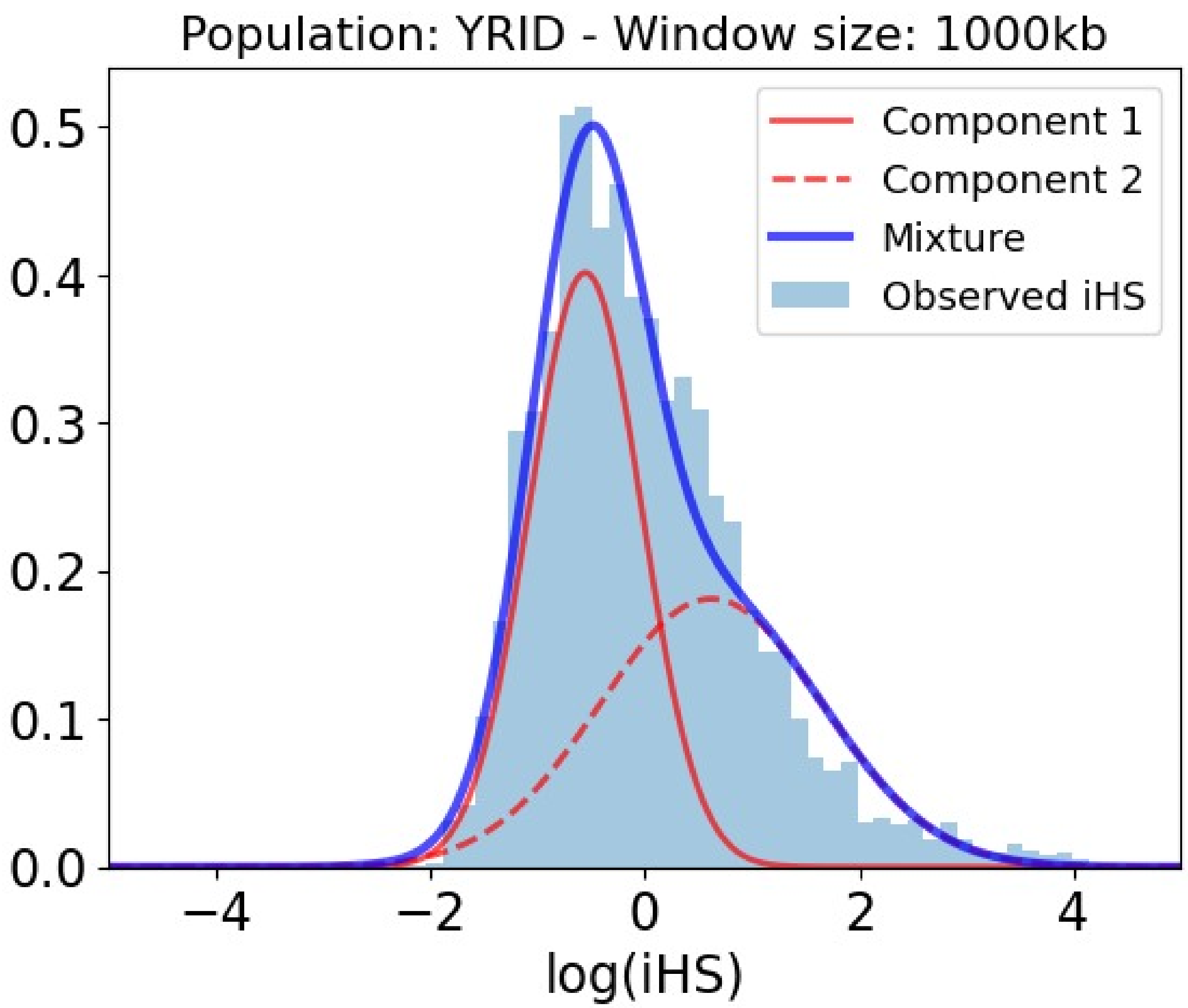

Table S3: Slopes and p-values of the association between iHS and genomic factors for the Yoruba population in 1000kb within the selection-enriched component.

**Covariate**	**Slope**	**P-value**
Intercept	-0.145	4.730E-03
Number iHS data points	-0.139	1.506E-02
Regulatory density (ChIP-seq)	0.496	9.345E-04
Regulatory density in immune cells (ChIP-seq)	0.846	1.267E-08
Recombination rate around regulatory elements in lymphocytes (ChIP-seq)	0.319	2.955E-06
Number of recombination rate data points around regulatory elements in lymphocytes (ChIP-seq)	-0.890	0.000E+00
Regulatory density in testis (ChIP-seq)	-0.483	1.498E-06
Coding density	-0.092	5.957E-01

Covariate	Slope	P-value
Density of conserved elements	-0.067	3.747E-01
Gene expression	-0.286	7.175E-03
GC-content	0.546	1.056E-03
Gene length	0.100	1.016E-01
Gene number	0.090	5.836E-01
Gene expression in immune cells	0.221	2.202E-02
Number PPIs	0.025	6.538E-01
Recombination rate	-0.859	0.000E+00
Regulatory density (DNaseI)	-0.506	4.277E-03
Gene expression in testis	0.046	5.458E-01
Distance to VIPs	-0.235	2.347E-04

Yoruba 1000kb: Recombination around testis regulatory elements without original recombination

Figure S9: Mixture of Gaussian distributions fitting observed iHS (1000kb windows) for Yoruba. The figure shows the two Gaussian distributions, component 1 and 2 of iHS, being the latter enriched in positive selection. In that component, iHS linearly depends on the genomic factors considered. The figure shows iHS after log transformation and scaling (see Methods). Legend: Light blue = Observed iHS; Dark blue = Mixture model; Full red curve = Component 1 of the mixture model; Dashed red curve = Component 2 of the mixture model enriched in positive selection.

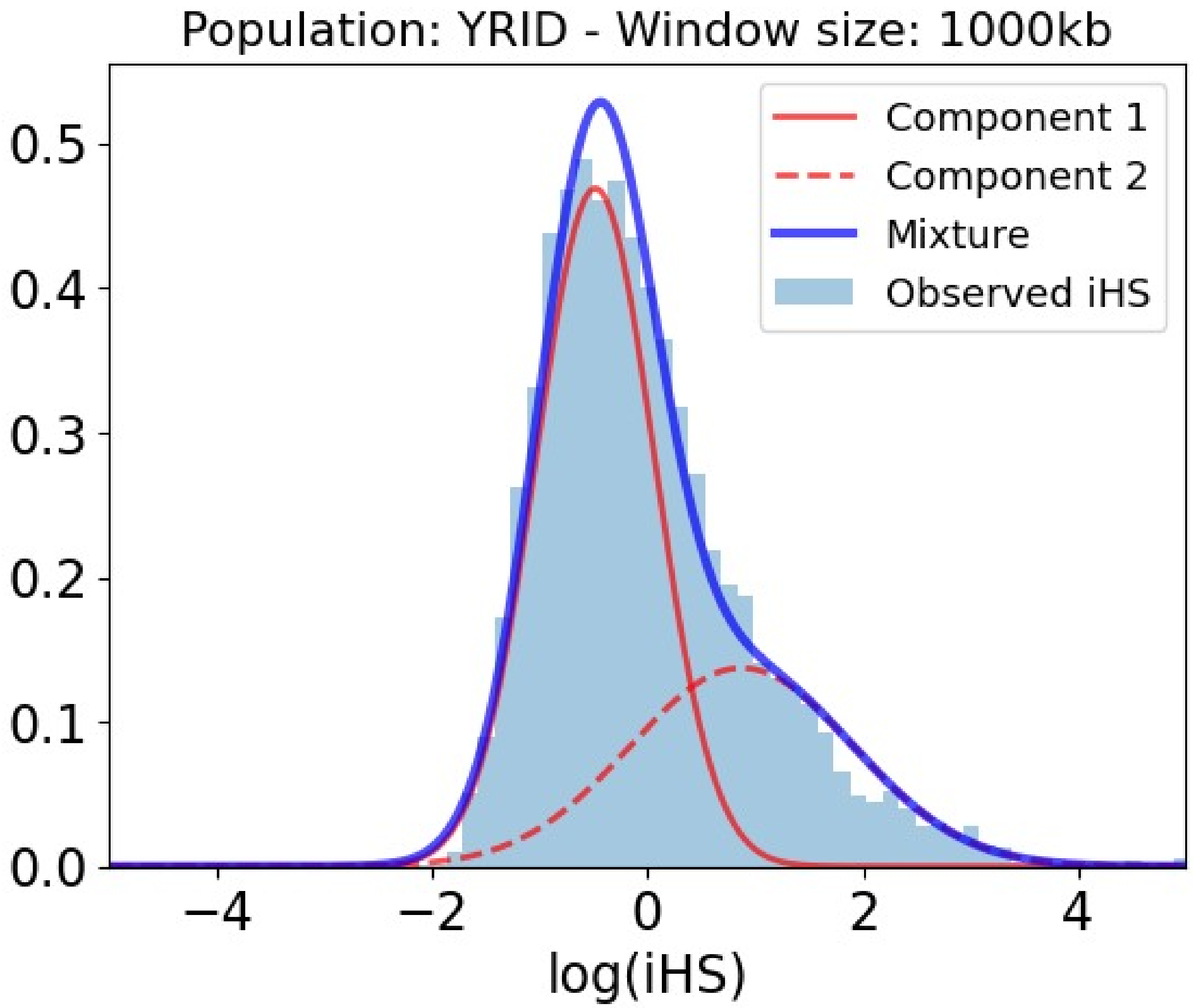

Table S4: Slopes and p-values of the association between iHS and genomic factors for the Yoruba population in 1000kb within the selection-enriched component.

**Covariate**	**Slope**	**P-value**
Intercept	-0.759	0.000E+00
Number iHS data points	-0.513	0.000E+00
Regulatory density (ChIP-seq)	-0.273	2.761E-03
Regulatory density in immune cells (ChIP-seq)	0.348	1.221E-05
Recombination rate around regulatory elements in testis (ChIP-seq)	-0.145	5.775E-07
Number of recombination rate data points around regulatory elements in testis (ChIP-seq)	-1.123	0.000E+00
Regulatory density in testis (ChIP-seq)	0.063	3.198E-01
Coding density	0.389	6.015E-06
Density of conserved elements	0.194	2.905E-06

Covariate	Slope	P-value
Gene expression	-0.056	3.396E-01
GC-content	-0.206	2.687E-02
Gene length	-0.075	2.275E-02
Gene number	0.008	9.198E-01
Gene expression in immune cells	0.149	5.850E-03
Number PPIs	-0.087	4.562E-03
Regulatory density (DNaseI)	0.115	2.891E-01
Gene expression in testis	0.065	1.136E-01
Distance to VIPs	-0.254	9.681E-14

Yoruba 1000kb: Recombination around testis regulatory elements and original recombination

Figure S10: Mixture of Gaussian distributions fitting observed iHS (1000kb windows) for Yoruba. The figure shows the two Gaussian distributions, component 1 and 2 of iHS, being the latter enriched in positive selection. In that component, iHS linearly depends on the genomic factors considered. The figure shows iHS after log transformation and scaling (see Methods). Legend: Light blue = Observed iHS; Dark blue = Mixture model; Full red curve = Component 1 of the mixture model; Dashed red curve = Component 2 of the mixture model enriched in positive selection.

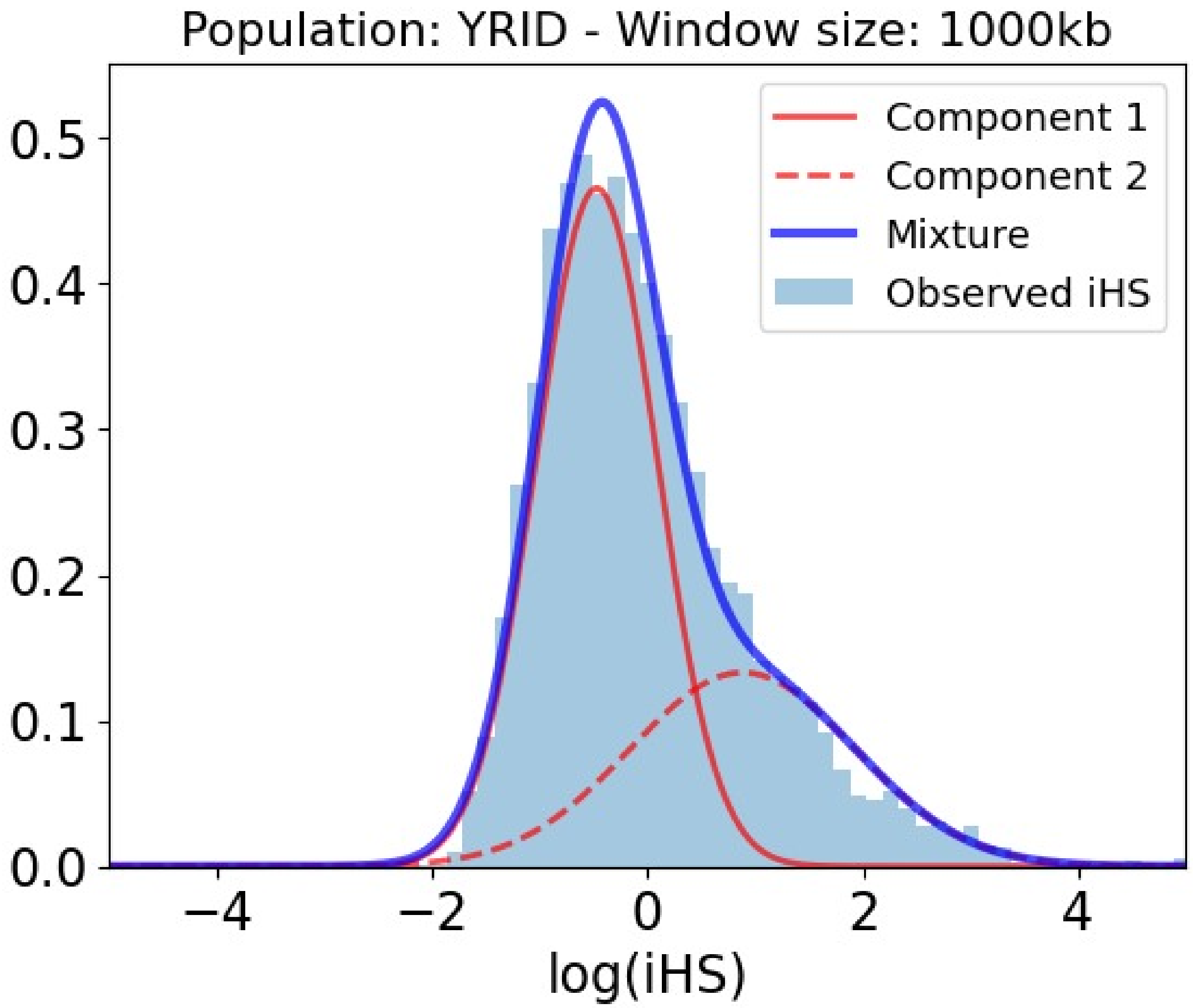

Table S5: Slopes and p-values of the association between iHS and genomic factors for the Yoruba population in 1000kb within the selection-enriched component.

**Covariate**	**Slope**	**P-value**
Intercept	-1.276	0.000E+00
Number iHS data points	0.026	4.333E-01
Regulatory density (ChIP-seq)	-0.188	1.065E-01
Regulatory density in immune cells (ChIP-seq)	0.132	1.644E-01
Recombination rate around regulatory elements in testis (ChIP-seq)	0.242	3.020E-08
Number of recombination rate data points around regulatory elements in testis (ChIP-seq)	-0.497	1.040E-11
Regulatory density in testis (ChIP-seq)	-0.550	1.548E-13
Coding density	0.195	4.638E-02
Density of conserved elements	0.179	2.236E-04

Covariate	Slope	P-value
Gene expression	-0.092	1.810E-01
GC-content	0.402	4.419E-04
Gene length	-0.067	7.885E-02
Gene number	-0.071	4.578E-01
Gene expression in immune cells	0.178	4.423E-03
Number PPIs	-0.071	4.914E-02
Recombination rate	-2.266	0.000E+00
Regulatory density (DNaseI)	0.176	2.050E-01
Gene expression in testis	0.016	7.354E-01
Distance to VIPs	-0.210	4.949E-08

Yoruba 1000kb: Recombination around testis regulatory elements and original recombination in high recombination regions

Figure S11: Mixture of Gaussian distributions fitting observed iHS (1000kb windows) for Yoruba. The figure shows the two Gaussian distributions, component 1 and 2 of iHS, being the latter enriched in positive selection. In that component, iHS linearly depends on the genomic factors considered. The figure shows iHS after log transformation and scaling (see Methods). Legend: Light blue = Observed iHS; Dark blue = Mixture model; Full red curve = Component 1 of the mixture model; Dashed red curve = Component 2 of the mixture model enriched in positive selection.

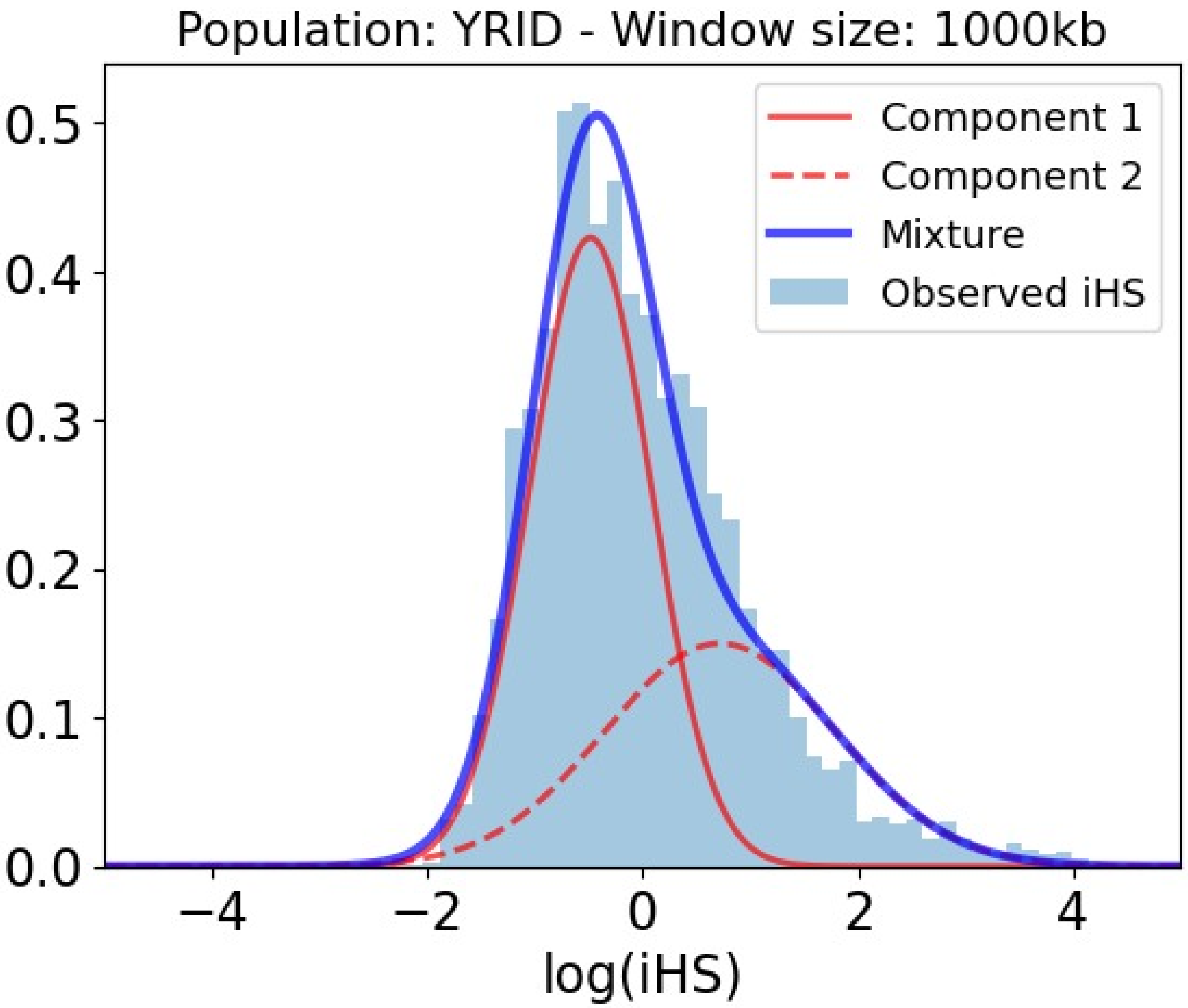

Table S6: Slopes and p-values of the association between iHS and genomic factors for the Yoruba population in 1000kb within the selection-enriched component.

**Covariate**	**Slope**	**P-value**
Intercept	-0.483	0.000E+00
Number iHS data points	-0.003	9.615E-01
Regulatory density (ChIP-seq)	0.427	2.304E-03
Regulatory density in immune cells (ChIP-seq)	0.366	5.361E-03
Recombination rate around regulatory elements in testis (ChIP-seq)	0.369	1.524E-10
Number of recombination rate data points around regulatory elements in testis (ChIP-seq)	-1.246	0.000E+00
Regulatory density in testis (ChIP-seq)	0.585	4.983E-05
Coding density	-0.295	9.542E-02
Density of conserved elements	0.038	6.080E-01

Covariate	Slope	P-value
Gene expression	-0.208	4.748E-02
GC-content	0.689	2.563E-05
Gene length	0.071	2.858E-01
Gene number	0.178	2.776E-01
Gene expression in immune cells	0.098	3.054E-01
Number PPIs	0.018	7.504E-01
Recombination rate	-0.854	0.000E+00
Regulatory density (DNaseI)	-0.548	1.262E-03
Gene expression in testis	0.046	5.309E-01
Distance to VIPs	-0.261	1.734E-04

Yoruba 1000kb: Recombination around regulatory elements without original recombination

Figure S12: Mixture of Gaussian distributions fitting observed iHS (1000kb windows) for Yoruba. The figure shows the two Gaussian distributions, component 1 and 2 of iHS, being the latter enriched in positive selection. In that component, iHS linearly depends on the genomic factors considered. The figure shows iHS after log transformation and scaling (see Methods). Legend: Light blue = Observed iHS; Dark blue = Mixture model; Full red curve = Component 1 of the mixture model; Dashed red curve = Component 2 of the mixture model enriched in positive selection.

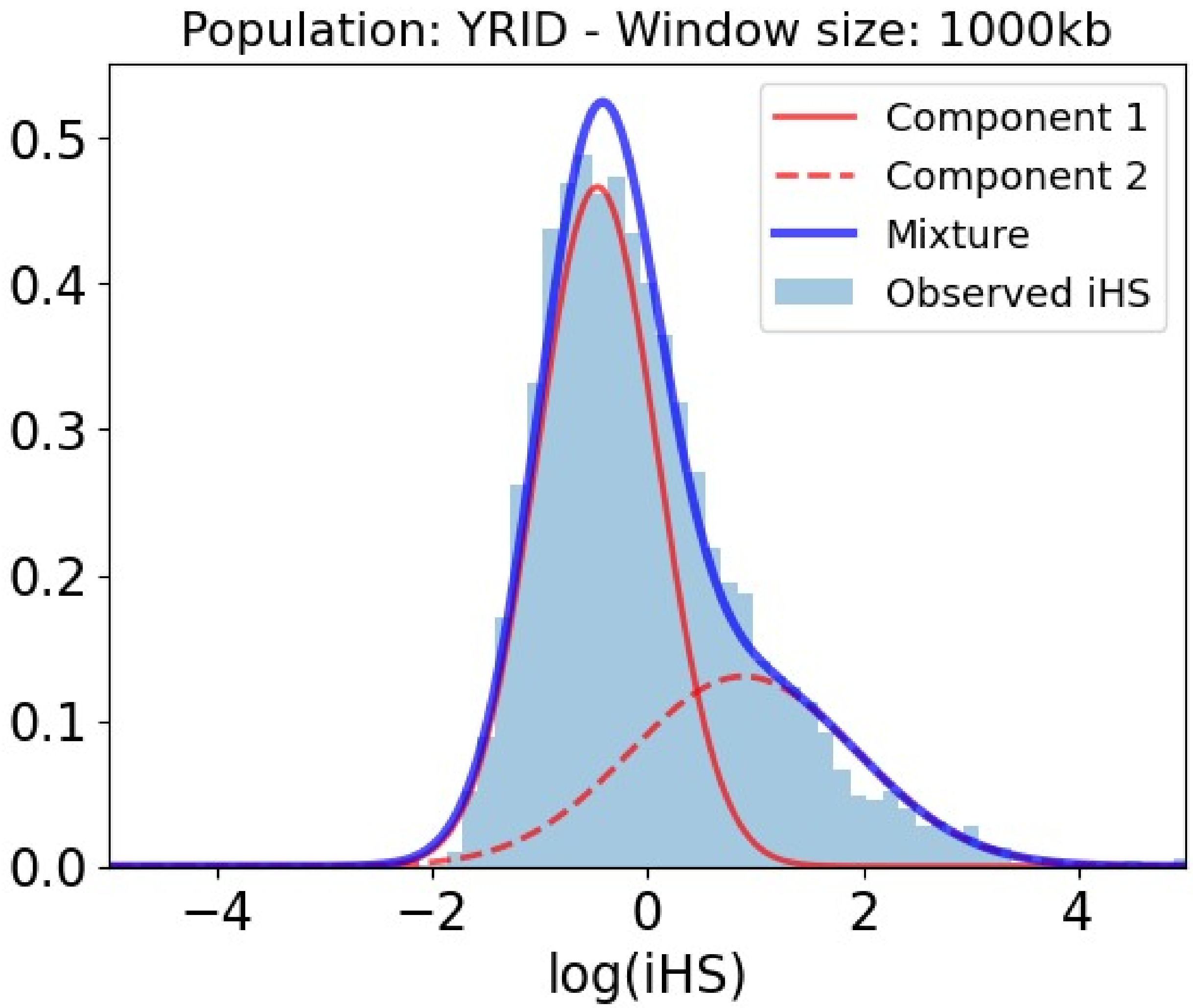

Table S7: Slopes and p-values of the association between iHS and genomic factors for the Yoruba population in 1000kb within the selection-enriched component.

**Covariate**	**Slope**	**P-value**
Intercept	-1.078	0.000E+00
Number iHS data points	0.044	1.602E-01
Regulatory density (ChIP-seq)	-1.955	0.000E+00
Regulatory density in immune cells (ChIP-seq)	0.487	1.563E-07
Recombination rate around regulatory elements (ChIP-seq)	-0.690	0.000E+00
Number of recombination rate data points around regulatory elements (ChIP-seq)	-1.835	0.000E+00
Regulatory density in testis (ChIP-seq)	-0.659	0.000E+00
Coding density	-0.002	9.713E-01
Density of conserved elements	0.382	3.519E-14
Gene expression	0.038	5.961E-01

Covariate	Slope	P-value
GC-content	0.238	5.356E-02
Gene length	-0.049	2.358E-01
Gene number	0.212	3.837E-02
Gene expression in immune cells	0.052	4.408E-01
Number PPIs	-0.054	1.461E-01
Regulatory density (DNaseI)	-0.242	7.496E-02
Gene expression in testis	0.002	9.728E-01
Distance to VIPs	-0.238	8.612E-08

Yoruba 1000kb: Recombination around regulatory elements and original recombination

Figure S13: Mixture of Gaussian distributions fitting observed iHS (1000kb windows) for Yoruba. The figure shows the two Gaussian distributions, component 1 and 2 of iHS, being the latter enriched in positive selection. In that component, iHS linearly depends on the genomic factors considered. The figure shows iHS after log transformation and scaling (see Methods). Legend: Light blue = Observed iHS; Dark blue = Mixture model; Full red curve = Component 1 of the mixture model; Dashed red curve = Component 2 of the mixture model enriched in positive selection.

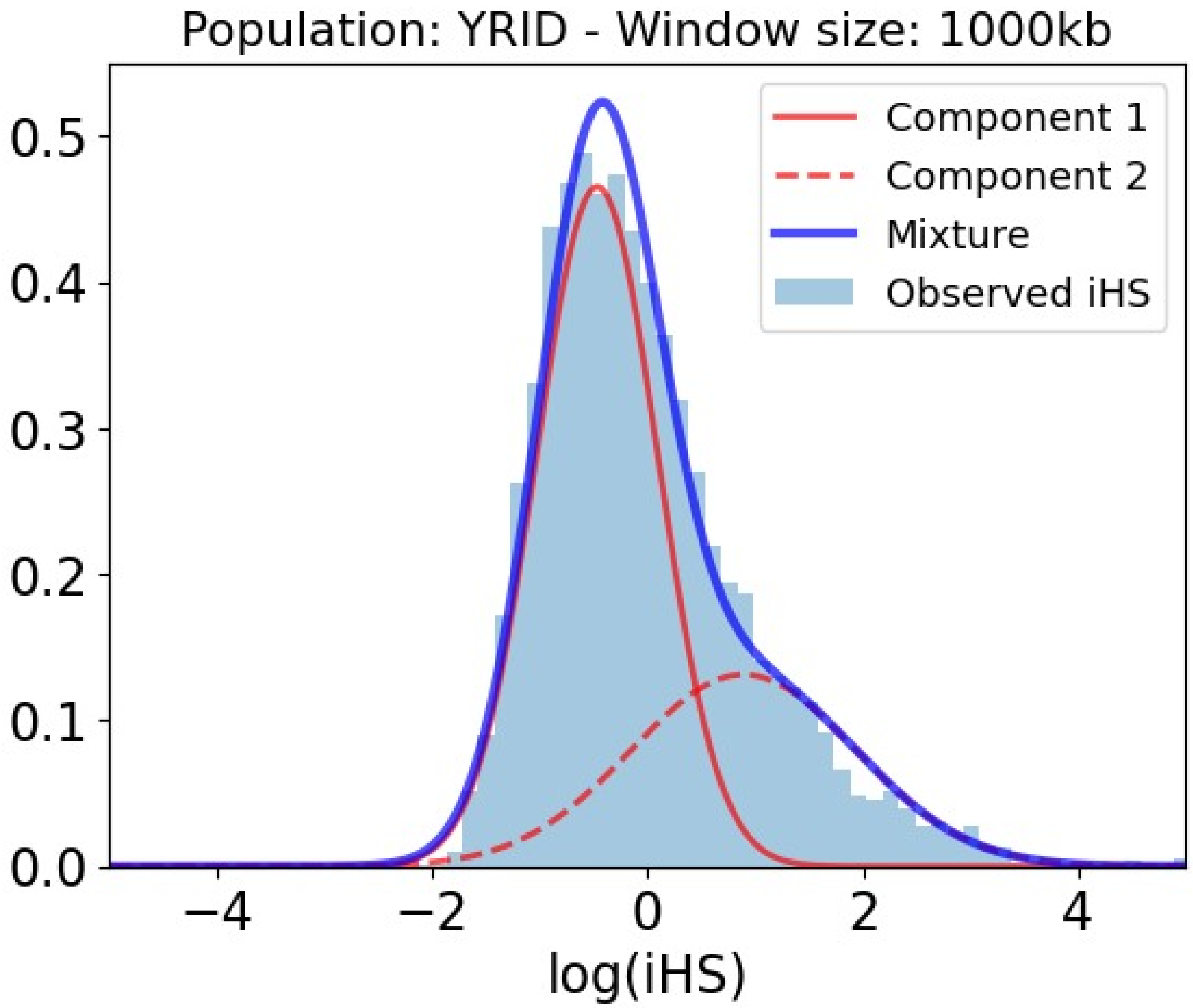

Table S8: Slopes and p-values of the association between iHS and genomic factors for the Yoruba population in 1000kb within the selection-enriched component.

**Covariate**	**Slope**	**P-value**
Intercept	-1.253	0.000E+00
Number iHS data points	0.161	1.396E-06
Regulatory density (ChIP-seq)	-1.322	0.000E+00
Regulatory density in immune cells (ChIP-seq)	0.188	5.430E-02
Recombination rate around regulatory elements (ChIP-seq)	0.044	5.366E-01
Number of recombination rate data points around regulatory elements (ChIP-seq)	-1.435	0.000E+00
Regulatory density in testis (ChIP-seq)	-0.801	0.000E+00
Coding density	-0.038	6.941E-01
Density of conserved elements	0.340	1.331E-10
Gene expression	0.006	9.245E-01

Covariate	Slope	P-value
GC-content	0.305	1.703E-02
Gene length	-0.053	2.168E-01
Gene number	0.173	9.452E-02
Gene expression in immune cells	0.068	3.229E-01
Number PPIs	-0.056	1.532E-01
Recombination rate	-1.515	0.000E+00
Regulatory density (DNaseI)	0.007	9.572E-01
Gene expression in testis	-0.014	7.941E-01
Distance to VIPs	-0.226	4.450E-07

Yoruba 1000kb: Recombination around testis regulatory elements and original recombination in high recombination regions

Figure S14: Mixture of Gaussian distributions fitting observed iHS (1000kb windows) for Yoruba. The figure shows the two Gaussian distributions, component 1 and 2 of iHS, being the latter enriched in positive selection. In that component, iHS linearly depends on the genomic factors considered. The figure shows iHS after log transformation and scaling (see Methods). Legend: Light blue = Observed iHS; Dark blue = Mixture model; Full red curve = Component 1 of the mixture model; Dashed red curve = Component 2 of the mixture model enriched in positive selection.

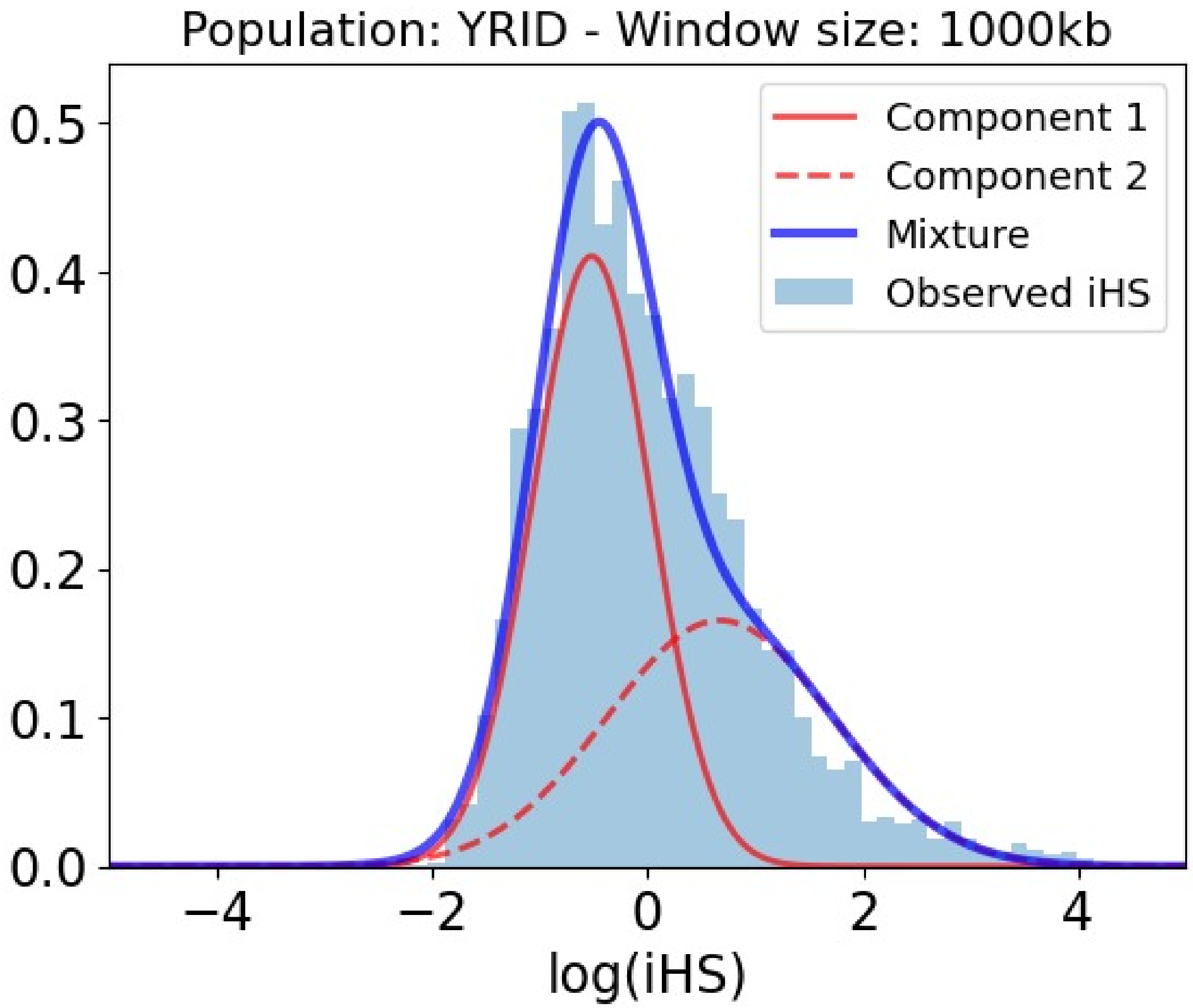

Table S9: Slopes and p-values of the association between iHS and genomic factors for the Yoruba population in 1000kb within the selection-enriched component.

**Covariate**	**Slope**	**P-value**
Intercept	-0.337	3.131E-11
Number iHS data points	-0.045	4.329E-01
Regulatory density (ChIP-seq)	-0.392	9.898E-03
Regulatory density in immune cells (ChIP-seq)	0.417	1.759E-03
Recombination rate around regulatory elements (ChIP-seq)	0.178	1.230E-02
Number of recombination rate data points around regulatory elements (ChIP-seq)	-0.921	0.000E+00
Regulatory density in testis (ChIP-seq)	-0.344	3.891E-04
Coding density	0.053	7.621E-01
Density of conserved elements	-0.050	4.960E-01
Gene expression	-0.246	1.793E-02

Covariate	Slope	P-value
GC-content	0.819	1.352E-06
Gene length	0.138	3.260E-02
Gene number	0.004	9.817E-01
Gene expression in immune cells	0.102	2.749E-01
Number PPIs	0.038	4.942E-01
Recombination rate	-0.735	0.000E+00
Regulatory density (DNaseI)	-0.878	1.660E-05
Gene expression in testis	0.068	3.611E-01
Distance to VIPs	-0.134	4.261E-02

Supplemental Results S5: Results using non-overlapping gene windows

We ran again the MDR model on the 5 studied populations but using this time only 1000kb gene windows that were not physically overlapped. In order to create this subset of genes, we randomly selected 1 gene window and then started an iterative process where a random gene window was selected and included in the subset if it was not physically overlapped with any of the 1000kb gene windows already included in such subset. This generated a subset of 1680 genes without physical overlapping between them that was then analyzed using the MDR approach as previously described. This new analysis provides evidence about the impact of non-independence between gene windows that are physically close.

Table S1: Slopes and p-values of the association between iHS and genomic factors for the five studied in 1,000 kb windows within the selection-enriched component. These results were generated using a subset of gene windows that are not physically overlapped.

Covariate	Yoruba		Utah residents		Toscani		Han Chinese		Peruvians	
	Slope	P-value	Slope	P-value	Slope	P-value	Slope	P-value	Slope	P-value
Intercept	-1.906	1.77E-12	-2.509	0.00E+00	-2.565	0.00E+00	-2.075	0.00E+00	-1.964	6.44E-15
Number iHS data points	-0.084	5.42E-01	0.125	2.50E-01	0.136	2.66E-01	0.029	7.31E-01	0.090	5.03E-01
Density of conserved elements	-0.133	4.01E-01	0.291	8.80E-02	0.083	6.01E-01	0.095	4.85E-01	0.062	6.91E-01
Recombination rate	-3.160	0.00E+00	-3.338	0.00E+00	-3.061	0.00E+00	-2.420	0.00E+00	-1.886	0.00E+00
Number PPIs	-0.007	9.59E-01	-0.074	6.03E-01	-0.041	7.55E-01	-0.037	7.67E-01	-0.026	8.38E-01
Regulatory density (ChIP-seq)	-0.350	3.96E-01	0.345	4.48E-01	0.289	5.65E-01	-0.005	9.89E-01	0.362	3.34E-01
Distance to VIPs	-0.217	1.79E-01	-0.480	3.19E-02	-0.416	4.92E-02	-0.269	1.70E-01	-0.564	4.55E-03
Gene number	-0.229	6.33E-01	-0.333	4.42E-01	-0.117	8.45E-01	-0.009	9.78E-01	-0.627	1.51E-01
Coding density	0.527	4.88E-01	0.506	3.32E-01	0.375	7.62E-01	0.004	9.89E-01	0.685	7.16E-02
Gene length	-0.123	4.25E-01	0.053	7.80E-01	0.130	4.62E-01	0.135	3.62E-01	0.025	8.79E-01
Regulatory density in immune cells (ChIP-seq)	0.478	1.41E-01	0.087	7.98E-01	0.327	2.97E-01	0.445	1.71E-01	1.146	2.37E-03
Gene expression	-0.079	7.40E-01	-0.391	1.11E-01	-0.430	6.99E-02	-0.420	5.76E-02	-0.179	4.33E-01
Gene expression in testis	0.124	4.72E-01	0.072	7.30E-01	0.183	3.32E-01	0.304	4.85E-02	0.289	8.43E-02
Gene expression in immune cells	0.134	5.59E-01	0.539	2.27E-02	0.405	8.28E-02	0.215	3.33E-01	-0.079	7.40E-01
Regulatory density in testis (ChIP-seq)	-0.604	1.25E-02	-0.654	6.52E-03	-0.620	1.06E-02	-0.450	4.18E-02	-0.736	3.39E-04
Regulatory density (DNaseI)	0.189	7.04E-01	0.001	1.00E+00	-0.069	8.99E-01	0.177	7.18E-01	-0.472	3.14E-01
GC-content	0.362	3.84E-01	0.481	2.69E-01	0.420	3.04E-01	0.518	1.84E-01	0.209	5.91E-01

Supplemental Results S6: Results of population simulations

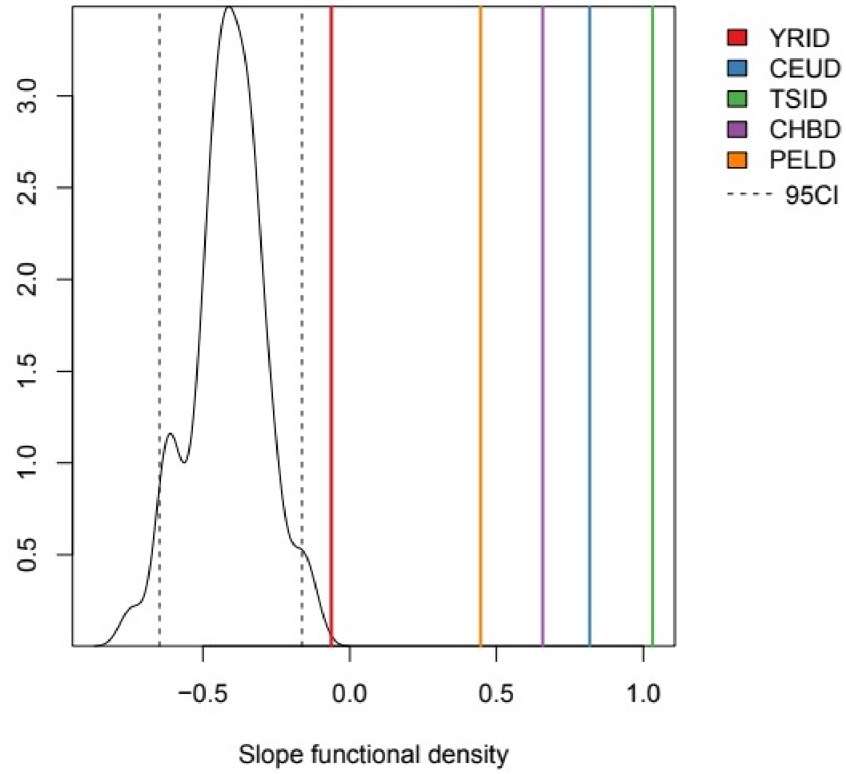

Figure S1: Distribution of the slope of functional (coding + regulatory) density across 100 neutral simulations (i.e., only neutral mutations). The 95% confidence interval of this slope is shown with dashed lines. Moreover, the slope of functional density observed in the actual populations is represented with different vertical lines. These slopes are calculated using the original set of predictors in each population but summing coding and regulatory density into a single predictor. Abbreviations: YRID = Yoruba; CEUD = Utah residents; TSID = Toscani; CHBD = Han Chinse; PELD = Peruvians.

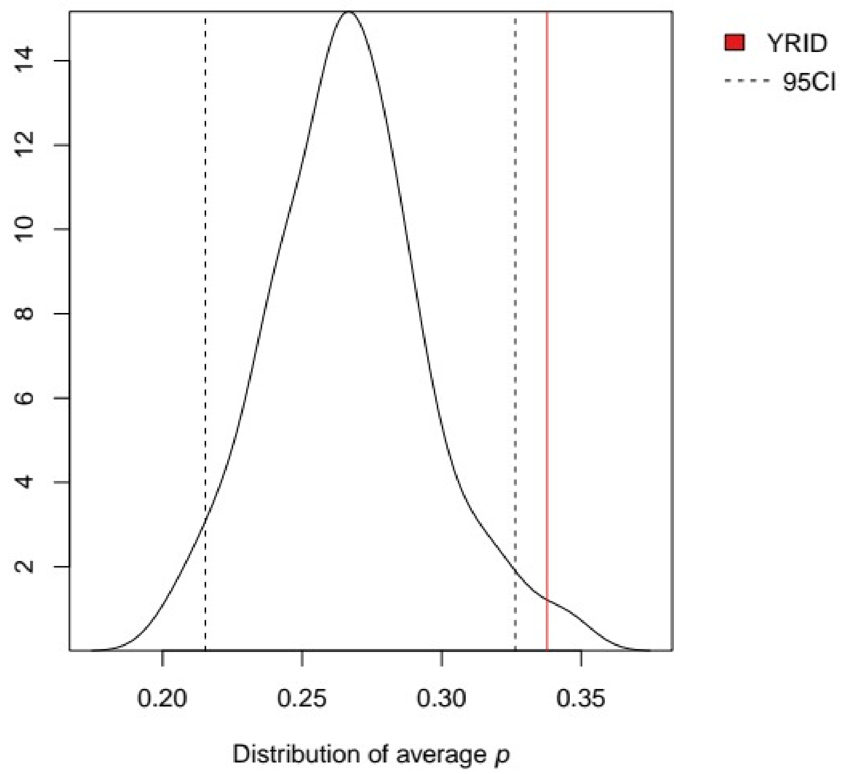

Figure S2: Distribution of average *p* across neutral simulations. The parameter *p* can be regarded as the cumulative effect of all genomic factors on the probability of the second component of iHS (see Methods). In each neutral simulation, we calculated the average of *p* across all gene windows, obtaining a total of 100 values. We have used this value as a metric for the magnitude of the second component of iHS. The 95% confidence interval of this parameter is shown with dashed lines. Moreover, the value observed for this parameter in Yoruba is represented with a red solid line. Abbreviations: YRID = Yoruba.

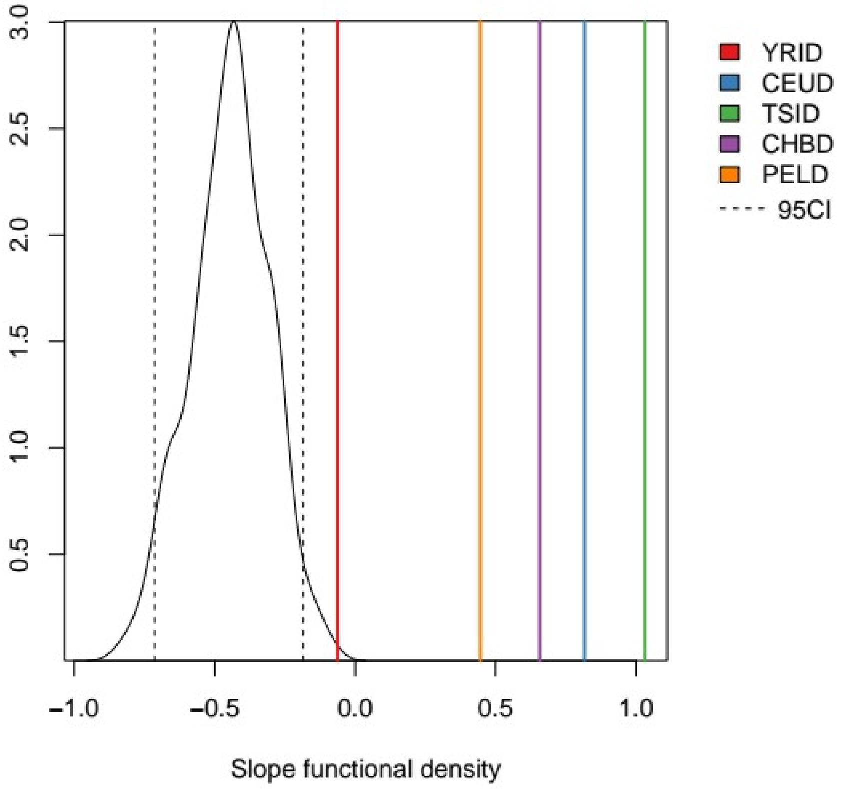

Figure S3: Distribution of the slope of functional (coding + regulatory) density across 100 simulations that included both neutral and deleterious mutations (i.e., negative and background selection). The 95% confidence interval of this slope is shown with dashed lines. Moreover, the slope of functional density observed in the actual populations is represented with different vertical lines. These slopes are calculated using the original set of predictors in each population but summing coding and regulatory density into a single predictor. Abbreviations: YRID = Yoruba; CEUD = Utah residents; TSID = Toscani; CHBD = Han Chinse; PELD = Peruvians.

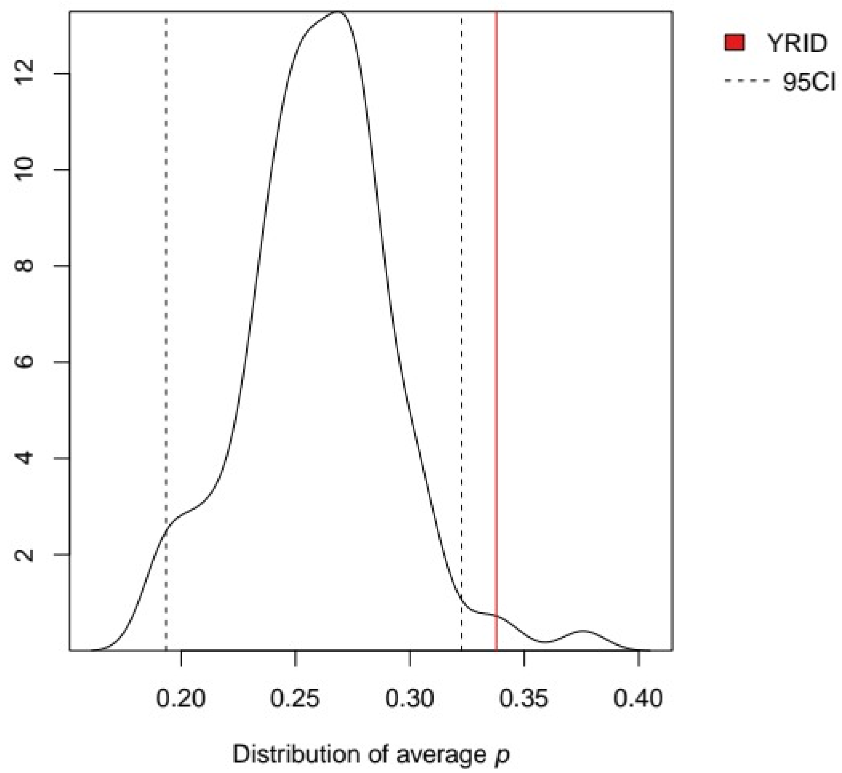

Figure S4: Distribution of average p across simulations that included neutral and deleterious mutations. The parameter p can be regarded as the cumulative effect of all genomic factors on the probability of the second component of iHS (see Methods). In each simulation, we calculated the average of p across all gene windows, obtaining a total of 100 values. We have used this value as a metric for the magnitude of the second component of iHS. The 95% confidence interval of this parameter is shown with dashed lines. Moreover, the value observed for this parameter in Yoruba is represented with a red solid line. Abbreviations: YRID = Yoruba.

Supplemental Results S7: Results of outlier approaches

We analyzed our data using two approaches based on outlier detection, i.e., we defined a threshold of iHS in order to split 1000kb gene windows between outliers and non-outliers. In one case, we used as threshold the iHS value corresponding with the peak of the selection-enriched component of a given population (MDR outlier approach hereafter). In the other case, we used the 95th percentile of iHS across the gene windows of the same population (95% outlier approach hereafter). Therefore, the former approach takes advantage of the MDRs's ability to prioritize regions enriched in positive selection signals while avoiding the use of arbitrary thresholds. We then calculated the average of each genomic factor within outliers and non-outliers. We tested whether the distribution of each factor was significantly greater in the group (outliers or non-outliers) with the greater average by using the Mann-Whitney U test. For example, we found that average recombination rate is higher in non-outliers, thus we tested whether the distribution of this factor was significantly greater in non-outliers compared to outliers. Using this approach, we analyzed all genomic factors measured in 1000kb windows across the 5 studied populations.

Table S1: Results of the MDR outlier approach. For each population, we used the iHS value at the peak of the selection-enriched component to divide gene windows between outliers and non-outliers. The table shows differences in the average of each genomic factor between outliers and non-outliers (avg outliers – avg non-outliers; "Avg diff"), along with a p-value for the existence of significant differences between the distribution of the factor in outliers compared to non-outliers.

Covariate	Yoruba		Utah residents		Toscani		Han Chinese		Peruvians	
	Avg diff	P-value	Avg diff	P-value	Avg diff	P-value	Avg diff	P-value	Avg diff	P-value
Number iHS data points	-0.452	1.63E-206	-0.170	8.42E-128	-0.040	6.37E-76	-0.159	3.51E-141	-0.140	4.06E-103
Density of conserved elements	0.163	1.52E-16	0.266	2.44E-40	0.249	3.95E-29	0.340	1.00E-57	0.411	3.11E-75
Recombination rate	-0.759	0.00E+00	-0.790	0.00E+00	-0.773	0.00E+00	-0.722	2.70E-304	-0.687	2.27E-269
Number PPIs	-0.016	3.15E-01	-0.002	5.62E-01	-0.008	4.35E-01	0.001	5.04E-01	0.008	1.81E-01
Regulatory density (ChIP-seq)	-0.172	1.10E-11	-0.123	3.09E-06	-0.102	8.61E-05	0.047	1.08E-02	0.148	1.49E-11
Distance to VIPs	-0.120	5.52E-07	-0.107	5.62E-04	-0.094	1.37E-02	-0.127	1.37E-09	-0.153	1.84E-13
Gene number	0.050	7.19E-11	0.055	5.30E-06	0.119	3.92E-09	0.190	2.87E-19	0.393	7.47E-48
Coding density	0.100	1.10E-24	0.128	2.86E-19	0.200	5.63E-24	0.253	6.16E-36	0.428	1.15E-76
Gene length	-0.053	4.99E-01	-0.055	4.35E-01	-0.051	3.45E-02	-0.057	1.43E-03	-0.110	4.26E-09
Regulatory density in immune cells (ChIP-seq)	-0.064	1.70E-01	0.019	4.09E-01	0.043	1.16E-01	0.172	1.63E-12	0.304	2.38E-27
Gene expression	0.052	3.79E-03	0.071	3.88E-05	0.035	4.74E-03	0.114	1.45E-09	0.067	3.88E-05
Gene expression in testis	0.057	3.28E-05	0.090	9.06E-10	0.054	3.70E-05	0.149	4.72E-16	0.101	5.97E-09
Gene expression in immune cells	0.129	2.04E-08	0.167	1.54E-14	0.122	6.38E-09	0.189	1.66E-17	0.144	1.10E-10
Regulatory density in testis (ChIP-seq)	-0.096	2.85E-02	-0.069	2.04E-02	-0.052	7.98E-02	0.139	1.37E-12	0.304	2.87E-18
Regulatory density (DNaseI)	-0.203	6.31E-16	-0.203	7.94E-15	-0.215	4.29E-18	-0.013	6.07E-01	0.019	1.40E-02
GC-content	-0.161	6.86E-10	-0.163	4.45E-10	-0.178	3.43E-13	0.033	3.69E-02	0.064	4.35E-06

Table S2: Results of the 95% outlier approach. For each population, we used the 95th percentile of iHS across gene windows to divide these windows between outliers and non-outliers. The table shows differences in the average of each genomic factor between outliers and non-outliers (avg outliers – avg non-outliers; "Avg diff"), along with a p-value for the existence of significant differences between the distribution of the factor in outliers compared to non-outliers.

**Covariate**	**Yoruba**		**Utah residents**		**Toscani**		**Han Chinese**		**Peruvians**	
	**Avg diff**	**P-value**	**Avg diff**	**P-value**	**Avg diff**	**P-value**	**Avg diff**	**P-value**	**Avg diff**	**P-value**
Number iHS data points	-0.268	6.01E-75	0.304	1.00E+00	0.289	1.00E+00	0.387	1.00E+00	0.238	1.00E+00
Density of conserved elements	0.158	2.06E-04	0.242	1.65E-13	0.317	2.43E-18	0.329	2.95E-24	0.508	1.37E-39
Recombination rate	-0.842	8.82E-173	-0.807	1.06E-151	-0.809	8.41E-151	-0.806	8.19E-155	-0.706	1.92E-122
Number PPIs	-0.070	6.16E-02	-0.010	4.78E-01	0.006	4.40E-01	-0.016	4.44E-01	0.037	2.23E-01
Regulatory density (ChIP-seq)	-0.222	3.19E-08	-0.128	7.13E-04	-0.052	1.51E-01	-0.041	1.23E-01	0.294	6.19E-11
Distance to VIPs	-0.148	3.00E-04	-0.146	1.85E-03	-0.180	4.42E-05	-0.136	9.50E-04	-0.201	6.43E-09
Gene number	0.128	2.51E-08	0.237	7.46E-07	0.395	8.27E-18	0.306	1.06E-10	0.478	1.46E-24
Coding density	0.176	6.64E-18	0.304	5.27E-14	0.440	2.75E-30	0.418	1.90E-23	0.621	4.84E-45
Gene length	-0.099	1.23E-03	-0.056	2.61E-03	-0.098	2.21E-06	-0.068	2.12E-05	-0.096	1.40E-05
Regulatory density in immune cells (ChIP-seq)	-0.030	2.47E-01	0.106	9.01E-02	0.153	2.59E-03	0.193	1.54E-03	0.557	1.31E-19
Gene expression	0.032	8.54E-02	0.077	1.70E-03	0.049	5.19E-03	0.052	5.22E-03	0.149	8.43E-07
Gene expression in testis	0.052	1.35E-02	0.080	2.10E-04	0.075	1.58E-05	0.033	1.15E-02	0.211	5.07E-11
Gene expression in immune cells	0.149	1.16E-04	0.209	2.51E-09	0.194	3.94E-08	0.156	6.47E-07	0.240	3.53E-11
Regulatory density in testis (ChIP-seq)	-0.067	9.58E-02	-0.024	9.84E-03	0.047	3.34E-01	0.120	2.35E-02	0.670	6.62E-16
Regulatory density (DNaseI)	-0.242	1.01E-09	-0.263	7.87E-11	-0.170	6.33E-05	-0.143	1.89E-03	0.143	4.57E-05
GC-content	-0.111	1.83E-02	-0.127	5.24E-03	-0.048	3.48E-01	-0.020	7.37E-01	0.217	5.11E-12

Table S3: Results of the original MDR model (no outlier detection) for comparison. Slopes and p-values of the association between iHS and genomic factors for the five studied populations in 1,000 kb windows within the selection-enriched component. Note that this analysis considered the covariation of genomic factors, as they were simultaneously considered in each model.

Covariate	Yoruba		Utah residents		Toscani		Han Chinese		Peruvians	
	Slope	P-value	Slope	P-value	Slope	P-value	Slope	P-value	Slope	P-value
Number iHS data points	-0.007	8.19E-01	0.315	0.00E+00	0.330	0.00E+00	0.157	7.29E-08	0.081	1.51E-03
Density of conserved elements	0.159	6.40E-04	0.379	7.74E-13	0.337	2.70E-11	0.311	4.20E-10	0.057	2.43E-01
Recombination rate	-2.435	0.00E+00	-2.798	0.00E+00	-2.696	0.00E+00	-2.510	0.00E+00	-1.738	0.00E+00
Number PPIs	-0.070	4.25E-02	-0.050	1.95E-01	-0.041	2.67E-01	-0.088	1.92E-02	-0.037	3.00E-01
Regulatory density (ChIP-seq)	-0.147	2.37E-01	0.593	3.45E-06	0.742	3.06E-10	0.490	3.92E-05	0.327	7.64E-03
Distance to VIPs	-0.170	4.16E-06	-0.337	8.45E-14	-0.266	1.92E-10	-0.142	5.35E-04	-0.178	2.77E-05
Gene number	-0.086	3.46E-01	-0.521	1.28E-06	-0.397	5.91E-05	-0.460	6.23E-04	-0.021	7.96E-01
Coding density	0.159	8.41E-02	0.535	8.72E-06	0.586	3.77E-08	0.396	1.93E-03	0.202	2.73E-02
Gene length	-0.059	1.12E-01	-0.047	2.84E-01	-0.024	5.59E-01	0.004	9.13E-01	0.023	5.46E-01
Regulatory density in immune cells (ChIP-seq)	0.047	6.24E-01	-0.051	6.01E-01	-0.079	3.94E-01	-0.160	1.26E-01	0.146	1.32E-01
Gene expression	-0.139	3.77E-02	-0.335	9.70E-06	-0.253	4.92E-04	-0.245	1.14E-03	-0.261	2.56E-04
Gene expression in testis	0.001	9.90E-01	-0.031	5.45E-01	-0.040	4.21E-01	0.065	2.06E-01	0.053	2.61E-01
Gene expression in immune cells	0.265	1.51E-05	0.408	6.87E-09	0.366	5.34E-08	0.307	9.51E-06	0.251	1.56E-04
Regulatory density in testis (ChIP-seq)	-0.795	0.00E+00	-1.031	0.00E+00	-0.861	0.00E+00	-0.393	1.65E-09	-0.107	4.76E-02
Regulatory density (DNaseI)	-0.047	7.15E-01	-0.609	5.45E-05	-0.635	8.24E-06	-0.838	1.66E-08	-0.551	8.27E-05
GC-content	0.550	1.34E-06	0.745	1.35E-08	0.516	2.47E-05	1.171	0.00E+00	0.351	2.63E-03

Table S4: Results of the MDR and 95% outlier approaches along with results of the original MDR model (i.e., no outlier detection). In the first two cases, we used an iHS threshold (iHS at the peak of the selection-enriched component and iHS 95th percentile, respectively) to divide outliers and non-outliers, then testing for significant differences in the distribution of each factor between both groups. In the last case, the table shows results about the association between each factor and iHS according to the MDR approach. In all cases, the p-value is shown for each genomic factor, while cells are colored based on the sense of the association: i) Red: the distribution of the genomic factor is significantly greater in outliers (outlier approaches) or the factor shows a positive and significant association with iHS (MDR approach); ii) Blue: the distribution of the genomic factor is significantly less in outliers or the factor shows a negative and significant association with iHS; Gray: there is no significant enrichment/depletion of the factor in outliers nor it is significantly associated with iHS.

Covariate	MDR threshold					95% threshold					Original MDR model				
	Yoruba	Utah residents	Toscani	Han Chinese	Peruvians	Yoruba	Utah residents	Toscani	Han Chinese	Peruvians	Yoruba	Utah residents	Toscani	Han Chinese	Peruvians
Number iHS data points	1.63E-206	8.42E-128	6.37E-76	3.51E-141	4.06E-103	6.01E-75	1.00E+00	1.00E+00	1.00E+00	1.00E+00	8.19E-01	0.00E+00	0.00E+00	7.29E-08	1.51E-03
Density of conserved elements	1.52E-16	2.44E-40	3.95E-29	1.00E-57	3.11E-75	2.06E-04	1.65E-13	2.43E-18	2.95E-24	1.37E-39	6.40E-04	7.74E-13	2.70E-11	4.20E-10	2.43E-01
Recombination rate	0.00E+00	0.00E+00	0.00E+00	2.70E-304	2.27E-269	8.82E-173	1.06E-151	8.41E-151	8.19E-155	1.92E-122	0.00E+00	0.00E+00	0.00E+00	0.00E+00	0.00E+00
Number PPIs	3.15E-01	5.62E-01	4.35E-01	5.04E-01	1.81E-01	6.16E-02	4.78E-01	4.40E-01	4.44E-01	2.23E-01	4.25E-02	1.95E-01	2.67E-01	1.92E-02	3.00E-01
Regulatory density (ChIP-seq)	1.10E-11	3.09E-06	8.61E-05	1.08E-02	1.49E-11	3.19E-08	7.13E-04	1.51E-01	1.23E-01	6.19E-11	2.37E-01	3.45E-06	3.06E-10	3.92E-05	7.64E-03
Distance to VIPs	5.52E-07	5.62E-04	1.37E-02	1.37E-09	1.84E-13	3.00E-04	1.85E-03	4.42E-05	9.50E-04	6.43E-09	4.16E-06	8.45E-14	1.92E-10	5.35E-04	2.77E-05
Gene number	7.19E-11	5.30E-06	3.92E-09	2.87E-19	7.47E-48	2.51E-08	7.46E-07	8.27E-18	1.06E-10	1.46E-24	3.46E-01	1.28E-06	5.91E-05	6.23E-04	7.96E-01
Coding density	1.10E-24	2.86E-19	5.63E-24	6.16E-36	1.15E-76	6.64E-18	5.27E-14	2.75E-30	1.90E-23	4.84E-45	8.41E-02	8.72E-06	3.77E-08	1.93E-03	2.73E-02
Gene length	4.99E-01	4.35E-01	3.45E-02	1.43E-03	4.26E-09	1.23E-03	2.61E-03	2.21E-06	2.12E-05	1.40E-05	1.12E-01	2.84E-01	5.59E-01	9.13E-01	5.46E-01
Immune regulatory density	1.70E-01	4.09E-01	1.16E-01	1.63E-12	2.38E-27	2.47E-01	9.01E-02	2.59E-03	1.54E-03	1.31E-19	6.24E-01	6.01E-01	3.94E-01	1.26E-01	1.32E-01
Gene expression	3.79E-03	3.88E-05	4.74E-03	1.45E-09	3.88E-05	8.54E-02	1.70E-03	5.19E-03	5.22E-03	8.43E-07	3.77E-02	9.70E-06	4.92E-04	1.14E-03	2.56E-04
Testis gene expression	3.28E-05	9.06E-10	3.70E-05	4.72E-16	5.97E-09	1.35E-02	2.10E-04	1.58E-05	1.15E-02	5.07E-11	9.90E-01	5.45E-01	4.21E-01	2.06E-01	2.61E-01
Immune gene expression	2.04E-08	1.54E-14	6.38E-09	1.66E-17	1.10E-10	1.16E-04	2.51E-09	3.94E-08	6.47E-07	3.53E-11	1.51E-05	6.87E-09	5.34E-08	9.51E-06	1.56E-04
Testis regulatory density	2.85E-02	2.04E-02	7.98E-02	1.37E-12	2.87E-18	9.58E-02	9.84E-03	3.34E-01	2.35E-02	6.62E-16	0.00E+00	0.00E+00	0.00E+00	1.65E-09	4.76E-02
Regulatory density (DNaseI)	6.31E-16	7.94E-15	4.29E-18	6.07E-01	1.40E-02	1.01E-09	7.87E-11	6.33E-05	1.89E-03	4.57E-05	7.15E-01	5.45E-05	8.24E-06	1.66E-08	8.27E-05
GC-content	6.86E-10	4.45E-10	3.43E-13	3.69E-02	4.35E-06	1.83E-02	5.24E-03	3.48E-01	7.37E-01	5.11E-12	1.34E-06	1.35E-08	2.47E-05	0.00E+00	2.63E-03
